# A modular route to boron doped PAHs by combining borylative cyclisation and electrophilic C–H borylation†
†Electronic supplementary information (ESI) available: Experimental procedures, compound characterisation data, copies of NMR spectra, CV, absorption and emission data and crystallographic data. CCDC 1537995, 1538224, 1538258, 1538386, 1546328, 1546329, 1553723, 1553725 and 1555383. For ESI and crystallographic data in CIF or other electronic format see DOI: 10.1039/c7sc02793a


**DOI:** 10.1039/c7sc02793a

**Published:** 2017-09-28

**Authors:** D. L. Crossley, R. J. Kahan, S. Endres, A. J. Warner, R. A. Smith, J. Cid, J. J. Dunsford, J. E. Jones, I. Vitorica-Yrezabal, M. J. Ingleson

**Affiliations:** a School of Chemistry , University of Manchester , Manchester , M13 9PL , UK . Email: Michael.ingleson@manchester.ac.uk

## Abstract

Heteroatom doping into polyaromatic hydrocarbons (PAHs) is a powerful approach for modifying key physical properties, however, there are extremely few modular routes that enable facile formation of B-, B_2_- and B,N-(specifically not containing direct B–N bonds) doped PAHs despite the growing importance of these materials. Sequential, one pot borylative cyclisation/intramolecular electrophilic C–H borylation of naphthyl-alkynes provides a simple new route to access novel B-, B,N- and B_2_-doped (PAHs). The initial products, dihydronaphthalene/dihydroquinoline B-mesityl PAHs, were reacted with [Ph_3_C][BF_4_]/pyridyl base to form the oxidised B-, and B,N-doped PAHs. However, for B-triisopropylphenyl (Trip) PAH congeners oxidation has to be performed prior to Trip installation due to preferential oxidation of an isopropylaryl moiety to the styrene. This alternative sequence enables access to Trip-B-PAHs and to structurally constrained B and B_2_-PAHs. Analysis of the solid state structures and optoelectronic properties of these PAHs confirm that frontier orbital energies, extended packing structures, Stokes shift and quantum yields all can be rationally modified using this methodology. The simplicity of this synthetic approach makes it a powerful tool for rapidly generating novel bench stable boron doped PAHs, which is important for facilitating further structure–property relationship studies and the wider utilisation of these materials in optoelectronic applications.

## Abstract 2

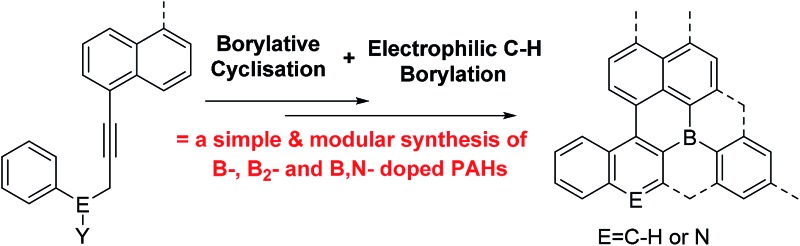
Starting from simple alkynes, sequential borylative cyclisation/electrophilic C–H borylation represents a simple modular route to novel B-doped PAHs.

## Introduction

**Fig. 1:**
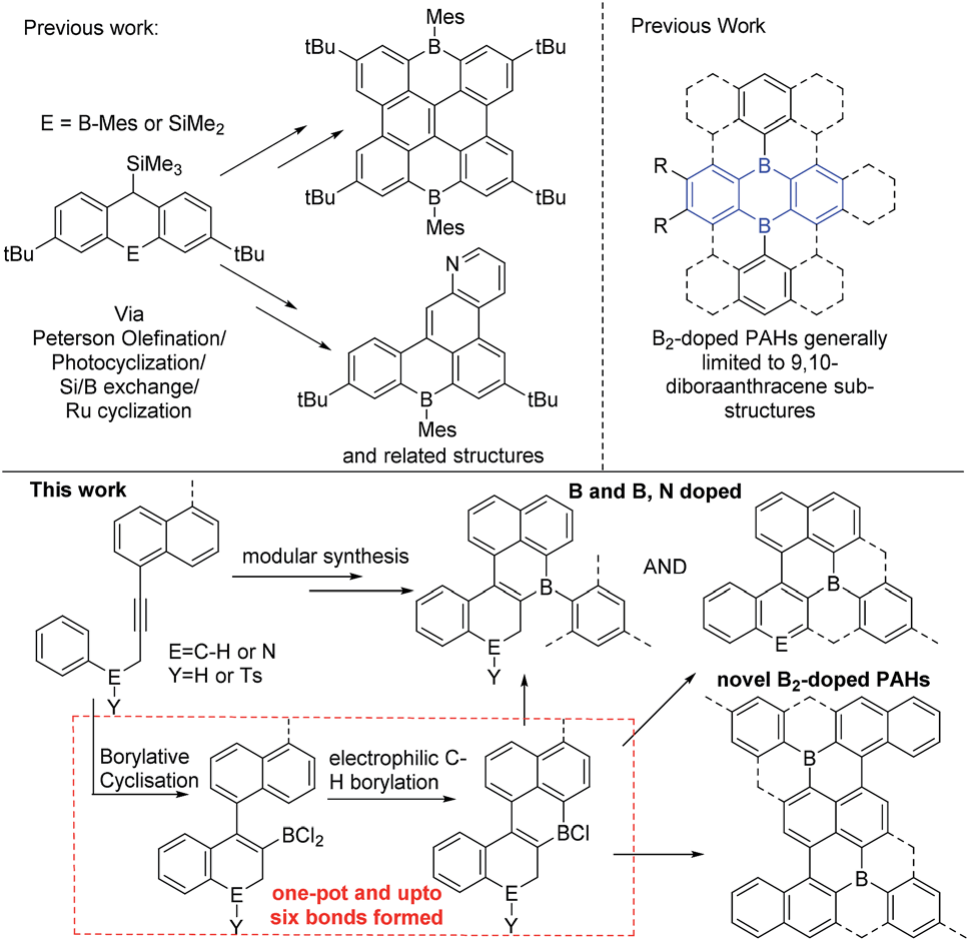
Top left, a current modular route to B doped PAHs. Top right, 9,10-dibora-anthracene based B_2_-PAHs. Bottom the modular route to novel B, B_2_ and B,N-doped PAHs reported herein.

Polyaromatic hydrocarbons (PAHs) are of significant topical interest as their attractive properties have resulted in a range of applications in the optoelectronic arena.^1^ Many key properties of PAHs are controlled by their frontier molecular orbitals, which in turn can be modulated by: (i) varying the size and shape of the PAH and, (ii) incorporating dopant (non-carbon) main group atoms.^2^ The introduction of electron deficient three coordinate boron atoms into PAHs is an attractive strategy to reduce the LUMO energy.^3^ Materials with low lying LUMO energy levels are highly desirable, for example as n-type or ambipolar semi-conductor materials.^4^ While there are many established routes to form all carbon PAHs, and PAHs doped with electron rich atoms,^2^ in contrast methods to form boron doped PAHs are relatively limited.^4^ While some notable progress has been made in this area this has principally generated mono-B and B–E (containing direct B–E bond(s), E = N or O) doped PAHs.^3^,^5^,^6^ This limitation significantly restricts the accessible compound space. Therefore the development of a simple and modular methodology that provides access to mono-B doped, and more importantly, to multiply B-doped and B, E doped (where there is no B–E bond) PAHs is highly desirable to facilitate fuller exploitation of these materials and enable greater understanding of structure property relationships. To the best of our knowledge there is only one modular route able to generate B, B_2_ and B, E (where there is no B–E bond) doped PAHs (Fig. 1, top left).^7^ This route requires the formation of 9-sila-anthracene (or 9-bora-anthracene) and then uses Peterson olefination, photocyclization, or ruthenium catalyzed cyclisation of aryl ene-ynes, and Si/B exchange reactions to form the B-PAHs.^7^ Therefore, new modular routes that are complementary to this work and ideally start from simpler precursors are required as a key enabler for this field.

We envisaged combining alkyne borylative cyclisation^8^ and intramolecular electrophilic C–H borylation^9^ to generate a versatile method to form B, B_2_ and B,N doped PAHs in one pot (Fig. 1, bottom). Naphthalene functionalised alkynes were selected as: (i) they are available in one step from commercial materials (*e.g.* aryl bromides and terminal alkynes), (ii) post borylative cyclisation they are correctly arranged to form six membered boracycles by intramolecular electrophilic borylation,^10^ and (iii) non-planar PAHs will be formed (due to the presence of cove regions) enhancing solubility.^11^ Herein we report that this single methodology facilely leads to B-, B_2_- and B,N-doped PAHs all from simple alkyne precursors. This represents a facile modular route to generate PAH materials containing novel dopant atom distributions. This approach also increases the diversity of B_2_-doped PAHs by accessing structures that are not based on 9,10-dibora-anthracene units (Fig. 1, top right, blue substructure), with the latter ubiquitous in the B_2_-PAHs reported to date.^3^,^12^

## Results and discussion

### Synthesis of mono-boron-PAHs

**Scheme 1:**
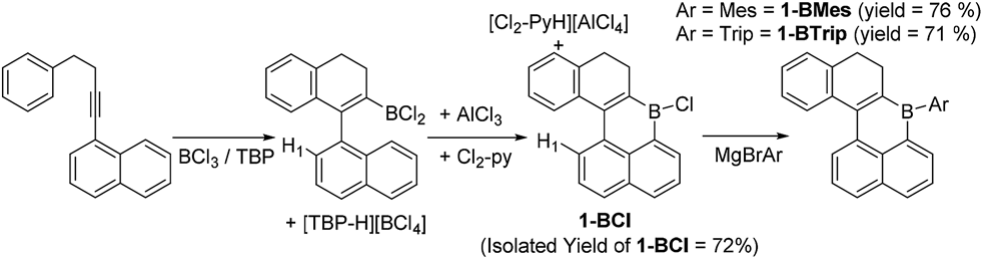
Sequential borylative cyclisation/C–H borylation to form boracycles.

**Fig. 2:**
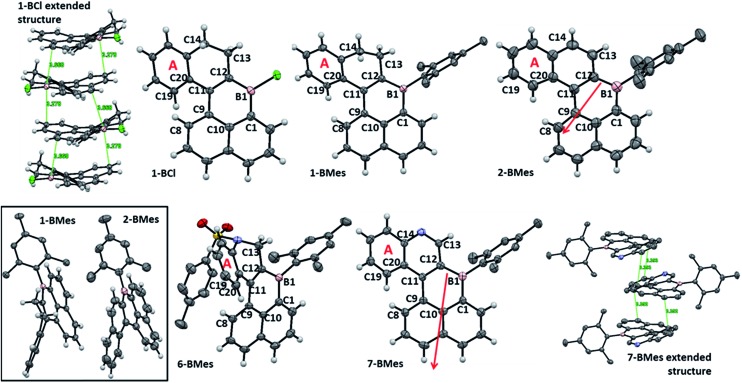
Solid state structures of B-PAHs. Ellipsoids at the 50% probability level, hydrogens on mesityl and tosyl groups are omitted for clarity. Top left and bottom right, extended structures for **1-BCl** and **7-BMes**. Red arrows indicate the dipole moment from the calculated structures of **2-BMes** and **7-BMes** at the M06-2X/6311G(d,p) level.

1-(4-phenylbut-1-yn-1-yl)-naphthalene has been previously demonstrated to undergo borylative cyclisation using BCl_3_/2,4,6-tritertbutyl pyridine (TBP) to form a vinyl BCl_2_ species.^8^ In this work instead of pinacol protection, the vinyl-BCl_2_ species is converted by intramolecular electrophilic C–H borylation to **1-BCl**. This is achieved by addition of stoichiometric quantities of AlCl_3_ and 2,6-dichloropyridine (Cl_2_-py).^13^ Compound **1-BCl** was isolated in 72% yield (Scheme 1) with no five membered boracycle derived from substitution of H1 observed. As an alternative to isolating **1-BCl**, the crude reaction mixture containing **1-BCl** and protonated pyridine by-products from S_E_Ar was reacted directly with MgBrMes to generate **1-BMes** (76% yield) or with MgBrTrip (Trip = triisopropylbenzene) to produce **1-BTrip** (71% yield). These are excellent yields for a three step one pot process forming four bonds direct from the alkyne. While **1-BCl** proved sensitive to moisture both **1-BMes** and **1-BTrip** were stable to non-purified solvents and to silica gel column chromatography. The formulation as **1-BCl**, **1-BMes** and **1-BTrip** was supported by multinuclear NMR spectroscopy (*δ*_11B_ = 51, 58 and 60 ppm, respectively) and mass spectrometry for the latter two species. Furthermore, X-ray diffraction studies were performed on single crystals of **1-BCl** and **1-BMes** (Fig. 2).

**Scheme 2:**
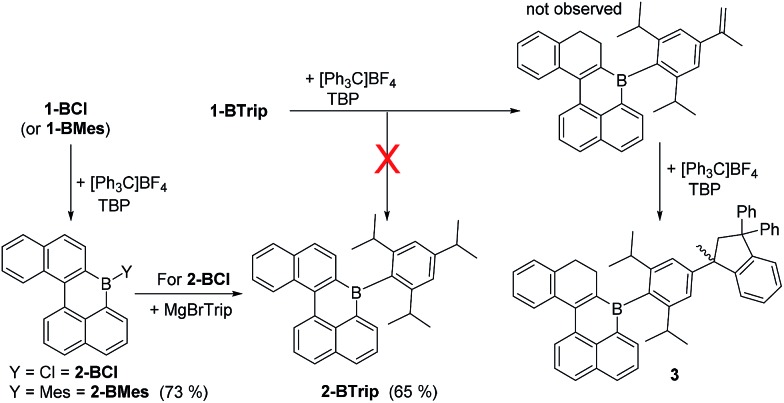
Oxidation of dihydronaphthalene B-PAHs.

The oxidation of BPin substituted dihydronaphthalenes to the naphthalene congeners using [Ph_3_C][BF_4_]/TBP in 1,2-dichloroethane (DCE) has been previously reported.^8^ This reagent combination was used to oxidise **1-BCl** to **2-BCl** and **1-BMes** to **2-BMes** (Scheme 2), with the latter amenable to recrystallization and X-ray diffraction studies. Notably, the oxidation procedure does not convert **1-BTrip** to **2-BTrip** and instead leads to a complex ^1^H NMR spectrum. This spectrum contained 1H integral diastereotopic resonances (at 3.66 and 3.20 ppm, ^2^*J*_HH_ = 13.1 Hz) with the two triplets for the CH_2_ moieties of the dihydronaphthalene persisting suggesting that the locus of reactivity is at an isopropyl group. This was supported by reacting isopropylbenzene with [Ph_3_C][BF_4_]/TBP which led to similar diastereotopic resonances in the ^1^H NMR spectrum to that observed using **1-BTrip**. [Ph_3_C][BF_4_] and α-methylstyrene are reported to undergo a formal [3 + 2] *cyclo*-addition reaction to form 1-methyl-1,3,3-triphenylindane,^14^ and this indane is the product formed from the reaction of isopropylbenzene with [Ph_3_C][BF_4_]/TBP. Thus on heating, [Ph_3_C][BF_4_]/TBP oxidises isopropylbenzenes to the respective styrene, which then undergoes a rapid reaction with a further equivalent of [Ph_3_C]^+^ to generate the indane. Therefore the product from the reaction of **1-BTrip** with [Ph_3_C][BF_4_]/TBP is the indane **3** (Scheme 2, bottom right), fully consistent with multinuclear NMR spectroscopy and mass spectrometry (and **3** is isolated in 52% yield). To the best of our knowledge this frustrated Lewis pair type oxidation of isopropylbenzenes is unprecedented.^15^ Using an alternative sequence **2-BTrip** was accessible in good yield (65%) from **1-BCl** by first oxidising to form **2-BCl** using [Ph_3_C][BF_4_]/TBP, and then *in situ* addition of MgBrTrip to form **2-BTrip**. **2-BTrip** was not amenable to crystallization in our hands, although the NMR data for **2-BMes** and **2-BTrip** are comparable (excluding the Mes/Trip resonances) and both have broad ^11^B resonances centered at 61 ppm.

**Scheme 3:**
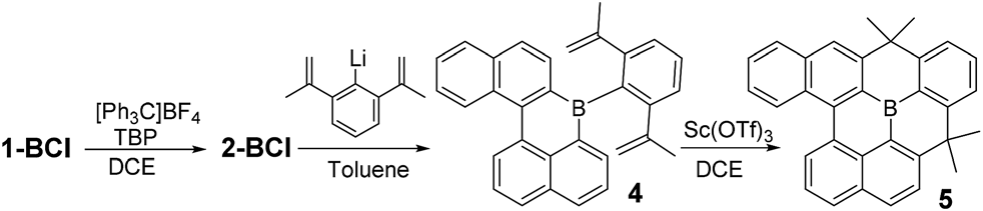
Synthesis of the structurally constrained B-PAH **5**.

With kinetic stabilization post borylative cyclisation/S_E_Ar using Mes and Trip groups demonstrated, the ability to kinetically protect the boron centre using the principle of structural constraint was explored.^16^ Structural constraint provides stability to ambient conditions by the rigid fused structure disfavouring pyramidalisation at boron. A previous example comparing the effects of protecting boron with mesityl groups *versus* structural constraint has indicated that there is minimal impact on frontier molecule orbital energies and character.^17^ However, there are dramatic effects on the extended solid state structures, thus both protection methods are important as they will lead to distinct bulk properties that are complementary for application purposes. Compound **1-BCl** was oxidized to **2-BCl** and then addition of (2,6-di(prop-1-en-2-yl)phenyl)lithium led to **4** (Scheme 3). In our hands **4** could not be isolated (and decomposed during column chromatography, consistent with previous reports on related compounds).^17^ Therefore, the crude reaction mixture derived from **1-BCl** was reacted with Sc(OTf)_3_ at 75 °C for 16 h to effect double Friedel Crafts cyclisation. The resultant fully fused B-PAH **5** proved stable to ambient conditions and could be isolated by column chromatography in 26% yield directly from **1-BCl** (the yield is only moderate but this is for 4 bond forming steps without any intermediate purification steps). While suitable single crystals were not obtained NMR and mass spectrometry were consistent with this formulation. Notably, the *δ*_11B_ for **5** at 45 ppm is shifted upfield from that for **2-BMes**/**2-BTrip** (*δ*_11B_ 61 ppm) consistent with a planarised boracycle structure.^17^

**Scheme 4:**
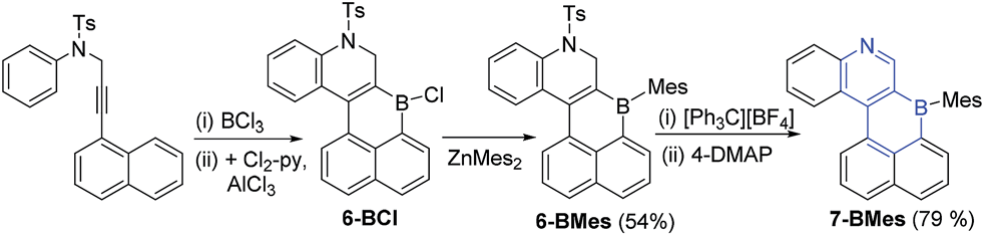
Borylative cyclisation/S_E_Ar to form **6-** and **7-BMes** after oxidation.

With the methodology demonstrated for B-doped PAHs we next extended it to form B,N-doped PAHs. The borylative cyclisation/electrophilic borylation one pot methodology generates a B,N-doped PAH from 4-methyl-*N*-(3-(naphthalen-1-yl)prop-2-yn-1-yl)-*N*-phenylbenzene-sulfonamide (this alkyne is accessible in one step from commercial materials). Compound **6-BCl** (Scheme 4) is generated in good conversion provided ≥ 2 eq. of AlCl_3_ are utilised, presumably the first equivalent of AlCl_3_ interacts preferentially with Lewis basic sites in the N-Ts moiety preventing activation of the vinylBCl_2_ until a second equivalent of AlCl_3_ is introduced. Post extraction of the crude mixture into toluene, compound **6-BCl** can be protected with ZnMes_2_ to afford **6-BMes**. MesMgBr was not effective in forming **6-BMes** as this reagent led to competitive *N*-tosyl deprotection^18^ and complex intractable mixtures. The dihydroquinoline moiety in **6-BMes** can be oxidised to furnish the quinoline congener, **7-BMes**, using [Ph_3_C][BF_4_], with tosyl removal then achieved by addition of 4-DMAP. Compound **7-BMes** was isolated in good yield (79%) with its formulation confirmed by multinuclear NMR spectroscopy (including a *δ*_11B_ = 60 ppm), mass spectrometry and X-ray diffraction studies. Pyridyl containing B-doped PAHs (such as **7-BMes**) are extremely rare, particularly those without B–N bonds.7*b* Indeed, the bicyclic B,N substructure in **7-BMes**, (a borinino[2,3-*c*]pyridine, highlighted in blue, Scheme 4) is to the best of our knowledge the first reported example of this substructure.

### Solid state structures of mono-boron PAHs

The solid state structures (Fig. 2) of **1-BCl**, **1-BMes**, **2-BMes**, **6-BMes** and **7-BMes** all contain trigonal planar boron centres and effectively planar boracycles. The mesityl substituents approach an orthogonal orientation to the polycyclic system in the latter four compounds. The short vinylic C11–C12 distances in **1-BCl**, **1-BMes** and **6-BMes** (1.377(3), 1.374(3), and 1.366(2) Å, respectively) and long vinylC-B distances (shortest B1–C12 = 1.522(3) Å in **1-BCl** which is comparable to the naphthylC-B distance in **1-BCl**, B1–C1 = 1.525(3) Å) indicate the structure is dominated by the C=C/vinylC-B resonance form. On oxidation to **2-BMes** and **7-BMes** the C11–C12 bond distances increase to 1.412(3) and 1.407(4) Å, respectively, as expected on transforming a vinyl into an aromatic system. Irrespective of oxidation level the C9–C11 bond distances are long (1.476–1.486 Å) indicating minimal aromaticity in the boracycle.

Despite containing essentially planar boracycles there is considerable deviation from planarity in the fused pentacyclic structures due to steric crowding in the cove region. The dihedral angles C19–C20–C9–C8 are 45.8°, 48.0° and 44.2° for **1-BCl**, **1-BMes** and **6-BMes**, respectively, which decrease to 36.9° in **2-BMes** and 32.8° in **7-BMes**. The greater deviation from planarity in **1-BCl**, **1-BMes** and **6-BMes** partly arises from hinging of the partially saturated ring (Fig. 2, bottom left) which leads to angles of 33.4°, 36.3° and 35.4°, respectively, between the mean plane of the boracycle and the mean plane of the six atoms in ring A. For **2-BMes** and **7-BMes** the greater rigidity arising from oxidation leads to a smaller angle between the mean plane of the boracycle and the mean plane of the six atoms in ring A (21.6° in **2-BMes** and 21.1° in **7-BMes**) as expected. A comparison of structural constraint *versus* orthogonal B-aryl groups is provided for the B_2_-PAHs where both have been characterised by X-ray diffraction.^17^

**Fig. 3:**
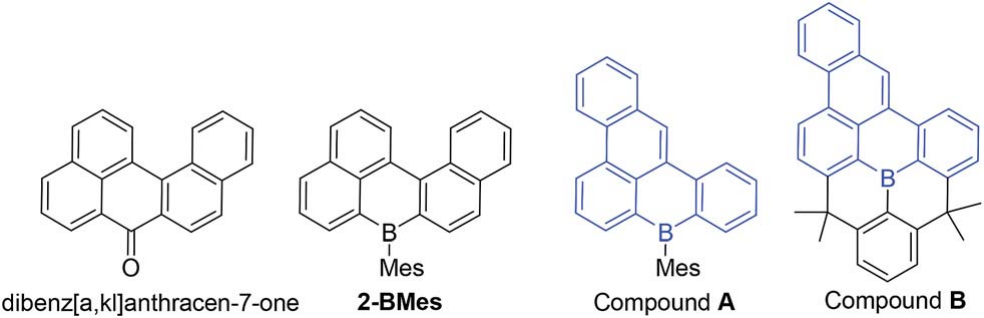
Isoelectronic (to **1-BCl**) and isomeric structures of **2-BMes** and **5**.

Examination of the extended structures reveal that **1-BCl** contains close packed 1D columnar formations (Fig. 2, top left) as expected as it does not contain any orthogonal aryl groups. While there is no significant differences in the structural metrics of the isoelectronic compounds **2-BMes** and **7-BMes**, comparison of the extended structures revealed notable differences. The former contains no close intermolecular contacts involving the pentacyclic core; however, **7-BMes** forms 1D columns with short intermolecular C–C contacts of 3.39 Å between pentacyclic cores of adjacent molecules (Fig. 2, bottom right). This disparity can be explained by the permanent dipole moment which is greater and has a different orientation in **7-BMes** relative to **2-BMes** (calculated dipole for **2-BMes** = 2.05 D and for **7-BMes** = 5.13 D at the M06-2X/6311G(d,p) level, see red arrows Fig. 2). Thus inclusion of an additional heteroatom dramatically alters the physical properties as expected by increasing the polarization within the PAH. This polarization favours forming a head to tail stacking to correctly align adjacent dipoles. A related outcome has been recently observed on incorporation of a B–C=C–N moiety to generate an indole derived-PAH.^10^ Comparison of **2-BMes** and **7-BMes** with dibenz[*a*,*kl*]anthracen-7-one (which is isoelectronic to **2-BCl**, Fig. 3, left) reveals a similar distortion from planarity in the pentacyclic structure with a C19–C20–C9–C8 angle of 33.4° in this fused cyclic-ketone.^19^ Compound **2-BMes** is an isomer of compound **A** and comparison reveals minimal differences in the B–C bond metrics with **2-BMes** and **A** having similar C–B–C angles and B–C distances. However, the different benzannulation pattern in **A** leads to an effectively planar structure due to the absence of cove regions in **A** (and in the structurally constrained isomer **B** which can be viewed as an isomer of **5**).^17^

### Synthesis of di-boron-PAHs

**Fig. 4:**
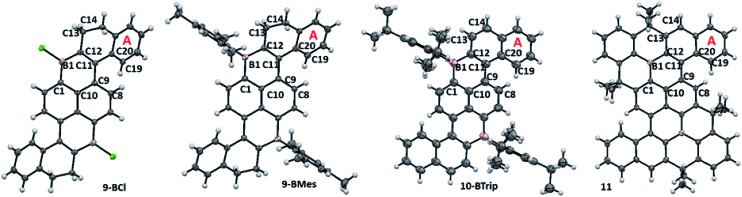
Solid state structures of B_2_-PAHs. Ellipsoids are drawn at the 50% probability level.

To further demonstrate the utility of sequential borylative-cyclisation/intramolecular C–H borylation an extended PAH containing two boron centres was targeted. Diyne **8** was synthesized in one step from 1,5-dibromonaphthalene and the commercially available terminal alkyne 3-butyn-1-yl-benzene. Notably, cyclisation of **8** will lead to a diboron-doped PAH containing a different arrangement of the two boron centres relative to that found in 9,10-dibora-anthracenes. Diyne **8** was converted to the double boracycle compound **9-BCl** in one pot using identical conditions to that successful for the mono-B-PAHs. Specifically, BCl_3_/TBP was used to achieve borylative cyclisation which then was followed by addition of AlCl_3_/Cl_2_-py to effect intramolecular electrophilic C–H borylation. Compound **9-BCl** is poorly soluble in chlorinated organic solvents and precipitated from solution on standing. This enabled its isolation in excellent yield (86% from **8**) for a one pot reaction forming four B–C bonds and two C–C bonds. The identification of **9-BCl** was based on ^1^H NMR spectroscopy, elemental analysis and an X-ray diffraction study (Fig. 4). Compound **9-BCl** can be converted to **9-BMes** by addition of MgBrMes.

Notably, **9-BMes** has lower stability towards moisture than **1-BMes**, although sufficient **9-BMes** was obtained for characterisation by multinuclear NMR spectroscopy and for an X-ray diffraction study. In contrast, **9-BTrip** was stable to ambient atmosphere and could be isolated post column chromatography as a red powder in 70% yield. Application of the oxidation conditions used for **1-BCl** ([Ph_3_C][BF_4_]/TBP in DCE) resulted in conversion of **9-BCl** to **10-BCl**. Compound **10-BCl** was protected *in situ* using MgBrTrip to produce **10-BTrip** in 45% isolated yield post chromatography. The formation of **10-BTrip** was confirmed by multinuclear NMR, mass spectrometry and an X-ray diffraction study. The ^11^B NMR chemical shifts for **9/10-BTrip** are centred at 62 ppm as expected for boron containing PAHs. Compound **9-BCl** also was converted through to the fully fused PAH **11** in one pot *via***10-BCl** in 11% isolated yield (while low this is an overall yield for oxidation/arylation/two Friedel Crafts cyclisations for each boracycle with no intermediate purifications performed, so is for 8 bond forming steps in one pot at *ca.* 57% yield per bond forming step). Compound **11** is a large PAH containing fourteen annulated rings that is stable to column chromatography and ambient conditions with an X-ray diffraction study confirming its successful formation.

**Scheme 5:**
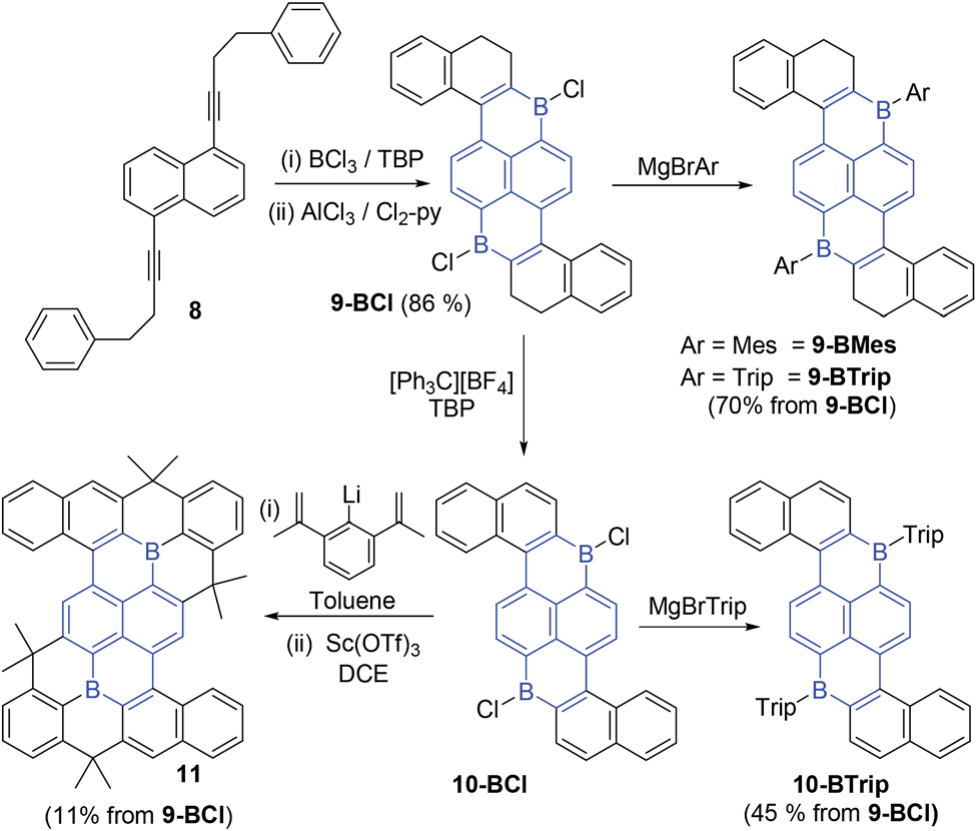
Synthesis of B_2_-doped PAHs, with the 1,6-diborapyrene core highlighted.

The combination of borylative cyclisation/electrophilic C–H borylation enables facile access to **9/10-BAryl** which contain a previously unknown arrangement of two boron atoms for a B_2_-PAH, (highlighted in blue in Scheme 5, while aza-borapyrenes have been previously reported,^20^ no diborapyrene based PAHs have been previously reported to the best of our knowledge). Overall the 1,6-di-borapyrene unit is accessed rapidly *via* a short sequence of linear steps (only two isolation steps required to form **9-BCl** starting from commercial materials). B_2_-PAHs that are not based on 9,10-dibora-anthracene units are extremely rare and those that have been reported to date are synthesised *via* relatively laborious methodologies (*e.g.* halogenation/lithiation/borylation) starting from complex precursors (*e.g.* 1,5-dihalo-2,6-ditriflato-naphthalenes),^21^ disfavouring the wider utilisation of these molecules.

### Solid state structures of B_2_-PAHs

The solid state structures of **9-BCl**, **9-BMes** and **10-BTrip** contain approximately planar B_2_C_14_ diborapyrene cores. Compounds **9-BCl**, **9-BMes** and **10-BTrip** have geometric trends similar to that discussed for the **1/2-B** series. For example, oxidation reduces the angle between the mean planes of ring A relative to that of the boracycle B1–C1–C10–C9–C11–C12 (for **9-BCl** and **9-BMes** = 39.2 and 34.8°, respectively, and for **10-BTrip** = 23.1°). Displacement distances for the B_2_C_14_ atoms from the mean plane of the diborapyrene unit are all < 0.15 Å for **9-BCl** and **9-BMes**, but they are larger for **10-BTrip** (all < 0.24 Å). The greater displacement distances of atoms from the diborapyrene mean plane in **10-BTrip** (despite a lower angle between the planes of the proximal boracycle and **A)** is presumably due to structural distortions to reduce steric crowding in the cove region as oxidation prevents the terminal bicyclic unit hinging to alleviate sterics (as observed in **9-BCl** and **9-BMes** analogous to **1-BMes**).

Close comparison of **11** with **10-BTrip** is noteworthy and revealed that **11** is less distorted from planarity (*e.g.* the angle between the mean plane of the boracycle B1–C1–C10–C9–C11–C12 and the mean plane of ring *A* = 20.8°). This is due to the more rigid structure formed on full fusion around boron which results in the linking of the naphthalene unit containing ring A to the diborapyrene core by three C–C bonds (in **11**) instead of two (in **10-BTrip**). The diborapyrene cores are generally comparable in metrics (excluding the shorter B–C distances in **11** analogous to that observed comparing **A** and **B** (Fig. 3))^17^ but in **11** it is more planar (deviations of atoms from the mean plane of the B_2_C_14_ core are all < 0.20 Å for **11**) than **10-BTrip**. Therefore in this case the protection at boron using the structural constraint approach increases the rigidity of the overall molecule and thus makes it a more planar PAH compared to protection using bulky aryl groups. The extended structures of **11** and **10-BTrip** are also notably different with **10-BTrip** containing no close intermolecular contacts involving the diborapyrene core and in fact only exhibiting one close face to face π–π contact involving the extended PAH system (through ring A, with one ring A approaching a ring A of an adjacent molecule above and below the plane with an intermolecular C–C distance of 3.348 Å, see Fig. S16†). In contrast, the extended structure of **11** contains multiple short intermolecular face to face π–π contacts, including involving the planarised diborapyrene cores of adjacent molecules (intermolecular C–C and B–C contacts = 3.351 and 3.395 Å see Fig. S17†). This results in a 1D columnar extended structure.

Notably, two symmetry related orthogonally orientated columnar structures are present in the extended structure which is attractive for providing charge mobility in two dimensions. This again highlights the ability of the structural constraint approach to engender more face to face π stacking interactions, as previously noted by Wagner and Yamaguchi.^17^

### Computational studies

**Fig. 5:**
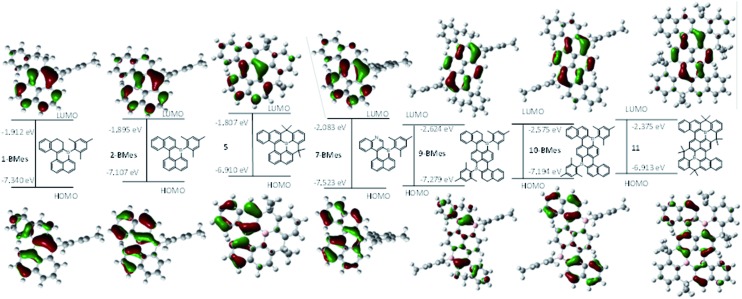
Molecular orbital energy levels and molecular orbital contours (isovalue = 0.04) of the HOMO and LUMO of the B, B,N and B_2_-doped PAHs calculated at the M06-2X/6311G(d,p) level with PCM DCM.

With eight B-doped PAHs in hand, structure–property relationships were investigated (**10-BTrip** was simplified to **10-BMes**). The calculated structures have similar metrics and trends to those observed in the solid state. **1-BMes**/**2-BMes** have similar energy and character LUMOs with significant boron character in both (Fig. 5). However, the HOMOs were more disparate with that of **2-BMes** being 0.233 eV higher in energy than for **1-BMes**. This is attributed to a greater degree of orbital delocalisation due to a more planar PAH structure post oxidation and a larger contribution to the HOMO from the additional C=C bond. Comparison of the frontier orbitals of **9-BMes** and **10-BMes** also revealed closely comparable energy and character LUMOs which are delocalised throughout the central diborapyrene unit with significant contribution from boron in both cases. The HOMO of the oxidised congener **10-BMes**, is higher in energy, which is attributed to the greater delocalisation of the HOMO on oxidation as discussed above for **2-BMes**. On comparison of the B- and B_2_-PAHs (*e.g.***2-BMes***vs.***10-BMes**) the most dramatic difference is in the energy of the LUMO which is calculated to be ∼0.7 eV lower in energy for the significantly more extended B_2_-PAH.

Comparison of **2-BMes**, **5** and **7-BMes** reveals minimal change in the distribution of the LUMO throughout the series. Whereas increasing the degree of planarization results in less boron character in the HOMO. The most notable change in this series is in the frontier orbital energies, with incorporation of the stronger acceptor quinoline moiety (relative to naphthalene) resulting in a significant reduction in the energy of the HOMO and LUMO relative to **2-BMes**. In contrast, fully fused **5** has higher HOMO and LUMO energies relative to **2-BMes**, which is consistent to that previously observed by Wagner and Yamaguchi for differentially protected 8H-8-borabenzo[*gh*]tetraphenes (compounds **A** and **B**, Fig. 3).^17^ This change in energy has been attributed previously to the inductive effect of the CMe_2_ substituents.

Analogous trends in frontier orbital energies are observed for **9-BMes**, **10-BMes** and **11**. However, a notable disparity between compounds **11** and **10-BMes** is that the HOMO is more delocalised in the former with significant character on the diborapyrene core. This is distinct to what was observed for compounds **A** and **B** where the frontier orbitals are effectively identical irrespective of the protecting strategy (mesityl *vs.* structural constraint). This disparity is presumably due to the fact that **A** and **B** both have fully planar PAH cores due to the absence of cove/bay regions, thus there are no geometric differences in the pentacycle units between compounds **A** and **B**. However, as discussed above, **11** is more planar than **10-BTrip** which will favour greater delocalisation of molecular orbitals, as observed in the HOMO for **11** compared to **10-BMes**. Therefore the different protecting methods available to stabilise boron doped PAHs, specifically structural constraint and orthogonal bulky aryls, can result in sufficient changes in geometry to impact the electronic structure of the boron doped PAHs.

Combined, the calculations/solid state structures indicate that this modular methodology to B-doped PAHs when coupled with the two different protecting strategies enables significant modification of (i) the frontier orbital energies (*e.g.***2-BMes***vs.***7-BMes**), (ii) the frontier orbital distribution (**1-BMes***vs.***5**) and, (iii) the extended structures (*e.g.***10-BMes***vs.***11**). These are all key variables that need to be controlled when optimising performance in organoelectronic devices.

### Optoelectronic properties

Cyclic voltammetry confirmed the calculated trends in the LUMO energies with the diboron PAHs **9-BTrip** and **10-BTrip** being more readily reduced by ∼0.55 eV than **1-BMes** and **2-BMes**. It is also notable that **9-BTrip** and **10-BTrip** display two reversible reduction waves; in contrast, **1-BMes** and **2-BMes** exhibit a second reduction wave at a much more negative potential that is irreversible for a range of scan rates. For both the B- and B_2_-PAHs the fully fused congeners have slightly more negative reduction potentials than the non fused derivatives in line with calculations and previous observations.^17^ Furthermore, the reduction waves are non-reversible for **5** and **11**. On replacing naphthalene for quinoline the reduction process occurs at a less negative potential (by 0.2 V for **7-BMes***vs.***2-BMes**), confirming that the stronger acceptor moiety results in a notable reduction of the LUMO energy.

Analysis of the photophysical data is also informative and supports the general trends derived from calculations (see ESI† for all the absorption and emission spectra). For example, the largest optical band gap is found for **7-BMes**, while the smallest is found for **10-BTrip** and **11** consistent with the calculated frontier orbital energies. The structurally constrained compounds **5** and **11** are significantly more emissive than their non-fused analogues **2-BMes** and **10-BTrip**, respectively (*Φ*_PL_ values 24 *vs.* 42% and 23 *vs.* 66%, respectively) and that the emission of **5** and **11** is blue shifted relative to **2-BMes** and **10-BTrip**, respectively. Again this is in contrast to compounds **A** and **B** which have closely comparable photophysical properties (see Table 1), but a related increase in *Φ*_PL_ has been observed on structurally constraining 9,10-dibora-anthracenes.16a,^22^ The smaller Stokes shift and higher quantum yields for **5** and **11** can be attributed to their more rigid structures (relative to **2-BMes** and **10-BTrip**), which can be expected to result in less structural reorganisation on excitation, and fewer rotational and vibrational degrees of freedom (reducing non-radiative decay pathways).^23^ Finally, compound **9-BTrip** is effectively non emissive, and has a low energy emission band. This compound shows concentration dependence of emission intensity with the lowest energy emission bands centred at 628 and 650 nm increasing in intensity (relative to that at 524 nm) on increasing the concentration (see Fig. S4†). This is attributed to excimer formation, which despite the presence of the Trip substituents is possible through intermolecular interactions involving the terminal naphthalene units as observed in the solid state structure of **10-BTrip** (which has face to face π stacking through ring A, Fig. S16†). In contrast, **10-BTrip** shows no evidence for concentration dependence in relative emission intensity.

**Table 1:** Electrochemical and photophysical data for the B-, B_2_- and B,N doped PAHs

Compound	1st reduction wave^*a*^			2nd reduction wave^*a*^						
	*E*_peak_ (V)	*E*_1/2_ (V)	LUMO (eV)	*E*_peak_ (V)	*E*_1/2_ (V)	*λ*max_abs_ (nm)^*b*^	*ε ×* 10^3^ (M^–1^ cm^–1^)	*λ*max_em_ (nm)^*b*^	Δ*E*_opt_ (eV)^*b*^	*Φ*_PL_ (%)^*c*^
**1-BMes**	–2.00	–1.92	–2.96	–2.72	—	351, 367, 420	16.1, 12.9, 4.1	516^*d*^	2.61	16^*d*^
**2-BMes**	–2.00	–1.92	–2.95	–2.65	—	336, 370, 428	15.8, 8.7, 7.2	512^*e*^	2.59	24^*e*^
**5**	–2.10	—	–2.87	—	—	310, 348, 431	11.6, 6.2, 3.3	486^*f*^	2.62	42^*f*^
**6-BMes**	–1.84	–1.77	–3.10	–2.60	—	418s, 378, 359	5.1, 14.7, 17.1	411, 433, 536^*g*^	2.54	7^*g*^
**7-BMes**	–1.80	–1.73	–3.18	–2.50	—	402, 374, 338	9.8, 10.2, 14.2	410, 478^*h*^	2.76	19^*h*^
**9-BTrip**	–1.40	–1.33	–3.53	–2.00	–1.93	320, 396, 413, 446, 476	51.0, 53.0, 49.4, 31.0, 28.5	524, 628, 650^*i*^	2.49	1^*i*^
**10-BTrip**	–1.46	–1.39	–3.48	–1.93	–1.86	318, 353, 487	46.5, 12.5, 21.5	592^*j*^	2.31	23^*j*^
**11**	–1.56	—	–3.38	–1.89	—	326, 375, 411, 483, 511	28.4, 9.7, 4.9, 15.1, 17.2	553^*k*^	2.30	66^*k*^
Compound A^*l*^	—	–2.05	—	—	—	311, 382, 400	22.4, 21.5, 30.9	411, 433, 460	3.02	85
Compound B^*l*^	—	–2.14	—	—	—	330, 379, 400	11.4, 22.3, 36.4	408, 430, 458	3.03	89

^*a*^Measured in THF (1 mM) with [^*n*^Bu_4_N][PF_6_] (0.1 M) as the supporting electrolyte at a scan rate of 50 mV s^–1^, potentials are given relative to Fc/Fc^+^ redox couple which is taken to be 4.80 eV below vacuum. LUMO energies calculated using the *E*_onset_ values (see Table S2).

^*b*^Measured at 1 × 10^–5^ M in toluene, optical band gap from the onset of absorption.

^*c*^absolute quantum yield values measured using an integrating sphere at 0.2 × 10^–5^ M in toluene (estimated error ± 10%), s = shoulder, *λ*_ex_ (nm).

^*d*^351.

^*e*^336.

^*f*^348.

^*g*^359.

^*h*^338.

^*i*^396.

^*j*^487.

^*k*^483.

^*l*^From ref. 17.

## Conclusions

In summary, the combination of borylative cyclisation and intramolecular electrophilic C–H borylation represents a facile route to synthesize a range of boron doped PAHs. The modularity has been proven by forming mono-B doped, doubly B-doped and B,N doped PAHs; with a number of these PAHs containing arrangements of dopant atoms that have not been reported previously. Structure property relationship studies on this series have highlighted a number of approaches to control key properties, such as frontier orbital energies (readily modified for example, by exchanging naphthalene for quinoline). This methodology is particularly attractive as the alkyne precursors are available in one step from commercial materials and thus this approach will enable access to many other novel B_*n*_-doped PAHs starting from suitably halogenated aromatic precursors (*e.g.*, 1,4-dibromonaphthalene, dibromoanthracenes *etc.*). Therefore the facile, modular synthesis disclosed herein will facilitate the elucidation of structure property relationships and the fuller exploitation of these materials across a range of optoelectronic applications.

## Conflicts of interest

There are no conflicts of interest to declare.

## Supplementary Material

Supplementary information

Crystal structure data
