# Supplementary material for: A modular route to boron doped PAHs by combining borylative cyclisation and electrophilic C–H borylation
Source: Chem Sci. 2017 Sep 28;8(12):7969–77. doi: 10.1039/c7sc02793a (PMC5853289; doi:10.1039/c7sc02793a)
Supplement: Supplementary file 1 [file SC-008-C7SC02793A-s001.pdf]

Electronic Supplementary Material (ESI) for Chemical Science.
This journal is © The Royal Society of Chemistry 2017

A Modular Route to Boron Doped PAHs by Combining Borylative Cyclization and Electrophilic C-H Borylation.

Daniel L. Crossley, Sebastian Endres, Rachel Kahan, Robert A. Smith, Andrew J. Warner, Inigo Vitorica-Yrezabal, Jessica Cid, Jay J. Dunsford, Jennifer Jones, Michael J. Ingleson*

School of Chemistry, University of Manchester, Manchester, M13 9PL, UK.

Table of Content

General Considerations S2
Synthetic Procedures S4
NMR Spectra S21
Photophysical Properties S45
Electrochemical Properties S51
Crystallographic Details and extended Packing Structures S55
DFT Calculations S65
Optimised Structure Coordinates S69
References S94

General Considerations

Unless otherwise indicated all reagents were purchased from commercial sources and were used without further purification. **1**[S1], MgBrTrip (0.72 M in THF)[S2] and 2-bromo-1,3-di(prop-1-en-2-yl)benzene[S3] were prepared according to modified literature procedures. All appropriate manipulations were performed using standard Schlenk techniques or in an argon-filled MBraun glovebox (O_2 levels below 0.5 ppm). Glassware was dried in a hot oven overnight and heated under vacuum before use. Solvents were distilled from NaK, CaH_2, or K and degassed prior to use. Dichloromethane and THF were stored over activated 3 Å molecular sieves while toluene was stored over a potassium mirror.

NMR spectra were recorded using a Bruker AV-400 spectrometer (400 MHz ^{1}H, 101 MHz $^{13}C\{^{1}H\}$, 128 MHz ^{11}B, 376 MHz $^{19}F\{^{1}H\}$). The chemical shift values of the ^{1}H NMR and $^{13}C\{^{1}H\}$ NMR spectra are reported in ppm relative to residual protio solvent (e.g. $CHCl_3$ in $CDCl_3$ $\delta H = 7.27$ or $\delta C = 77.2$) as internal standards. The $^{19}F\{^{1}H\}$ NMR spectra were referenced to C_6F_6, ^{11}B NMR spectra were referenced to external $BF_3{:}Et_2O$. Unless otherwise stated all NMR spectra are recorded at 293 K. Broad features in the ^{11}B NMR spectra are due to boron present in borosilicate glass. Carbon atoms directly bonded to boron are not always observed in the $^{13}C\{^{1}H\}$ NMR spectra due to quadrupolar relaxation leading to signal broadening. Coupling constants *J* are given in Hertz (Hz) as positive values regardless of their real individual signs. The multiplicity of the signals are indicated as “s”, “d”, “t”, “q”, “quin”, “sept” or “m” for singlet, doublet, triplet, quartet, quintet, septet, or multiplet, respectively. (br.) denotes a broad signal.

Matrix assisted laser desorption/ionization time of flight (MALDI-TOF), atmospheric pressure chemical ionization (APCI) and Electrospray ionization (ESI) measurements was performed by the Mass Spectrometry Service, School of Chemistry, University of Manchester. MALDI-TOF analyses were performed using a Shimadzu Axima Confidence spectrometer using a 4k PPG as a calibration reference. 1 µL of a solution of dopant NaI in THF (10 mg mL^{-1}) was spotted onto a well of the MALDI plate and the solvent left to evaporate. Solutions were made up to 10 mg mL^{-1} in DCM. A solution of matrix dithranol was made up to 10 mg mL^{-1} in THF. 2 µL of sample solution and 20 µL of matrix solution were thoroughly mixed and 1 µL of this solution was spotted onto a well with no dopant and 1 µL spotted by

a layered method with the NaI. The solvent was allowed to evaporate before being placed in the spectrometer. Samples were run in positive polarity mode in either linear or reflection mode. High resolution mass spectra (HRMS) were recorded on a Waters QTOF mass spectrometer. Microanalysis was performed by Mr Stephen Boyer at the London Metropolitan University microanalytical service.

All UV-vis absorption spectra were recorded on a Varian Cary 5000 UV-vis-NIR spectrometer at room temperature in spectroscopic grade solvents. Emission spectra were recorded on a Varian Cary Eclipse Fluorimeter at room temperature in spectroscopic grade solvents, the solutions were excited at their relative absorbance maxima. Absolute quantum yield values were recorded on an Edinburgh Instruments FP920 Phosphorescence Lifetime Spectrometer equipped with a 5 watt microsecond pulsed xenon flashlamp (with single 300 mm focal length excitation and emission monochromators in Czerny Turner configuration) and a red sensitive photomultiplier in peltier (air cooled) housing and determined using a calibrated Edinburgh Instruments integrating sphere.

Cyclic voltammetry was performed using a CH-Instrument 1110C Electrochemical/Analyzer potentiostat under a nitrogen flow. Measurements were made using a 1 mM analyte solution (compound **9** ~0.5 mM) with 0.1 M tetra^{n}butylammonium hexafluorophosphate (Fluka ≥99.0 %) as the supporting electrolyte in THF that had been degassed prior to use and obtained from a dry solvent system. A glassy carbon electrode served as the working electrode and a platinum wire as the counter electrode. An $Ag/AgNO_3$ non-aqueous reference electrode was used. All scans were calibrated against the ferrocene/ferrocenium (Fc/Fc^+) redox couple, which in this work is taken to be 4.8 eV below vacuum.[S4] The half-wave potential of the ferrocene/ferrocenium (Fc/Fc^+) redox couple ($E_{1/2,\ Fc,Fc+}$) was estimated from $E_{1/2,\ Fc,Fc+} = (E_{ap} + E_{cp})/2$, where E_{ap} and E_{cp} are the anodic and cathodic peak potentials, respectively.

Calculations were performed using the Gaussian09 suite of programmes.[S5] Structures were optimised with DFT method at the M06-2X/6-311G(d,p) level[S6] with PCM (Dichloromethane) solvation.[S7] In all cases, structures were confirmed as minima by frequency analysis and the absence of imaginary frequencies (tighter convergence criteria were required for 10-BMes, optimised using opt=tight

int=unltrafine)). Full Cartesian coordinates of the optimised geometries are provided below.

Synthetic Details

Synthesis of **1-BCl**

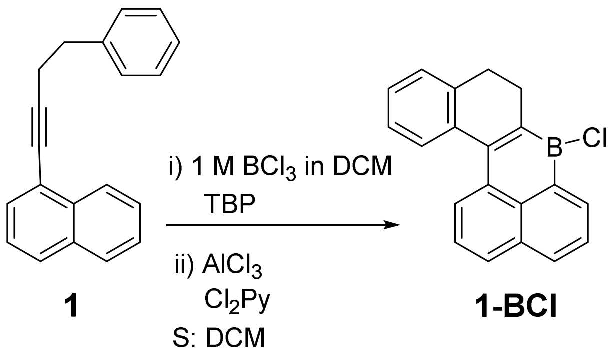

BCl_3 (1M in DCM) (2.0 mL, 2.0 mmol) was added to a solution of **1** (213 mg, 0.83 mmol) and 2,4,6-tri-tert-butylpyridine (TBP) (208 mg, 0.83 mmol) in DCM (10 mL). After 3 hours the solvent and volatiles (e.g. BCl_3) were removed under reduced pressure and the resulting residue was extracted with pentane (10 mL). The pentane was removed in *vacuo* and the resulting residue was redissolved in DCM (10 mL). 2,6-Dichloropyridine (208 mg, 0.83 mmol) and $AlCl_3$ (110 mg, 0.83 mmol) was added to the reaction mixture which underwent a colour change from yellow to green. The reaction mixture was then stirred for 20 minutes and the solvent was removed under reduced pressure to give a yellow residue. This residue was then extracted with a mixture of benzene:pentane (9:1) (10 mL) and evaporated to dryness to give the desired product as a yellow solid. Yield (178 mg, 72 %). Single crystals suitable for X-Ray diffraction were obtained by cooling a 1:1 DCM:pentane solution of **1-BCl** to -30°C.

^{1}H NMR (400 MHz, $CDCl_3$): δ= 8.81 (d, *J*=7.0 Hz, 1 H), 8.32 (d, *J*=7.8 Hz, 2 H), 8.10 (d, *J*=8.0 Hz, 1 H), 7.84 (t, *J*=7.5 Hz, 1 H), 7.68 (t, *J*=7.8 Hz, 1 H), 7.58 - 7.52 (m, 1 H), 7.45 - 7.33 (m, 3 H), 2.93 - 2.79 (m, 4 H);

^{13}C{^{1}H} NMR (101 MHz, $CDCl_3$): δ= 153.5, 141.8, 139.5, 136.3, 133.8, 133.5, 132.1, 131.8, 131.0, 130.7, 130.6, 128.4, 127.7, 126.3, 125.8, 125.4, 28.9, 26.2;

^{11}B NMR (160 MHz, $CDCl_3$): δ= 51;

Elemental Analysis = Calculated: **C** = 79.92%, **H** = 4.69%; Found: **C** = 79.77%, **H** = 4.55%

Synthesis of **1-BMes**

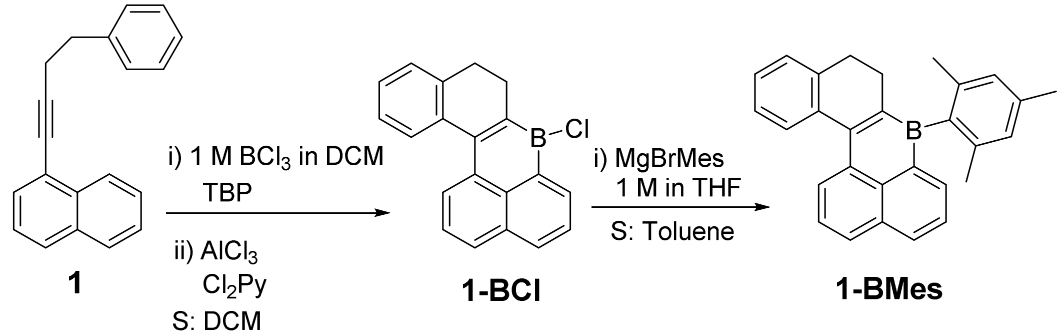

BCl_3 (1M in DCM) (7.8 mL, 7.80 mmol) was added to a solution of **A1** (800 mg, 3.12 mmol) and 2,4,6-tri-tert-butylpyridine (TBP) (780 mg, 3.12 mmol) in DCM (20 mL). After 3 hours the solvent and volatiles (e.g. BCl_3) were removed under reduced pressure and the resulting residue was redissolved in DCM (20 mL). $AlCl_3$ (416 mg, 3.12 mmol) was added to the reaction mixture which changed colour from pale yellow to orange/red. After 20 minutes the solvent and volatiles were then removed under reduced pressure (to remove the generated BCl_3) and the resulting residue was redissolved in DCM (8 mL). 2,6-Dichloropyridine (416 mg, 3.12 mmol) and $AlCl_3$ (416 mg, 3.12 mmol) was added to the reaction mixture which underwent a colour change from orange to dark green. The reaction mixture was then stirred for 20 minutes and the solvent was removed under reduced pressure to give a yellow residue. The residue was then suspended in toluene (20 mL). MgBrMes (1M in THF) (18 mL, 18 mmol) was added to the solution dropwise at ambient temperature. The reaction mixture was then stirred for 12 hours at ambient temperature were upon a colour change to pale green had occurred and all the solids had dissolved. The excess MgBrMes was quenched with a mixture of IPA: toluene (2:8) (60 mL). The reaction mixture was then passed through a short plug of silica gel and the resulting solution was evaporated to dryness. The resulting residue was purified via silica gel chromatography [eluent: DCM:Petroleum ether (2:8)]. The yellow fractions isolated contained a small amount of TBP so this mixture was further purified via silica gel chromatography [eluent: toluene:Petroleum ether (1:19)]. The desired product was isolated as a yellow solid. Yield 0.918 mg, 76 %. Single crystals suitable for X-Ray diffraction were obtained by slow evaporation from pentane.

^{1}H NMR (400 MHz, $CDCl_3$): δ= 8.46 (d, *J*=8.3 Hz, 1 H), 8.33 (dd, *J*=8.0, 1.3 Hz, 1 H), 8.20 (dd, *J*=6.8, 1.5 Hz, 1 H), 8.14 (d, *J*=7.5 Hz, 1 H), 7.67 - 7.79 (m, 3 H), 7.49 - 7.39 (m, 3 H), 7.01 (s, 2 H), 2.81 (t, *J*=7.0 Hz, 2 H), 2.59 (t, *J*=6.9 Hz, 2 H), 2.48 (s, 3 H), 2.12 (s, 6 H);
^{13}C{^{1}H} NMR (101 MHz, $CDCl_3$): δ= 151.3, 142.1, 142.0, 138.5, 136.4, 135.8, 134.3, 132.7, 132.2, 131.5, 131.2, 130.9, 130.8, 127.9, 127.6, 126.8, 126.3, 125.7, 125.2, 29.3, 27.9, 23.1, 21;
^{11}B NMR (128 MHz, $CDCl_3$): δ= 58 (br.);
HR-MS (HESI mode: positive): *m/z* calculated for $[C_{29}H_{25}B + H]^+$ 385.2128, found 385.2133.

Synthesis of **1-BTrip**

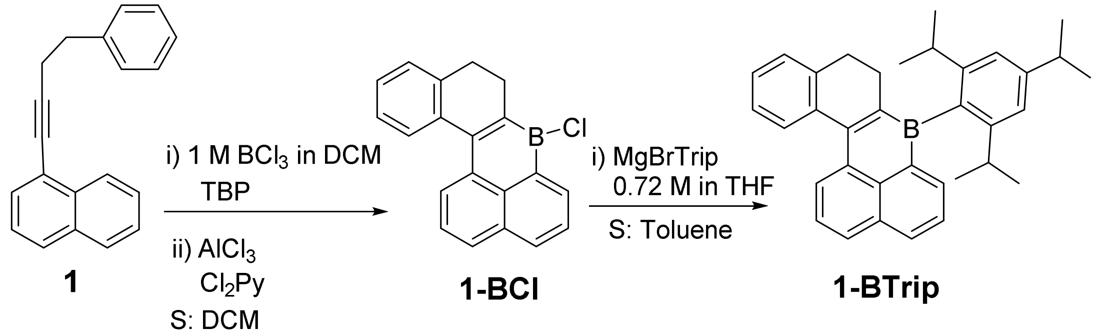

1 (205 mg, 0.8 mmol) and 2,4,6-tri-tert-butylpyridine (TBP) (198 mg, 0.8 mmol) were dissolved in DCM (10 mL) and BCl_3 (1M in DCM) (1.76 mL, 1.76 mmol) was added. The colour changed instantly from slightly yellow to bronze. After 5 minutes of stirring $AlCl_3$ (107 mg, 0.8 mmol) was added and the mixture stirred fora further 15 min and then all volatile components were removed *in vacuo*. The residue was redissolved in DCM (10 mL) and 2,6-dichloropyridine (118 mg, 0.800 mmol) and $AlCl_3$ (107 mg, 0.800 mmol) were added. The mixture was stirred for 2 hours, then (2,4,6-tri-isopropylphenyl)magnesium bromide (0.72 M in THF)(6.7 mL, 4.8 mmol) was slowly added and the reaction mixture was then stirred for 18 h. The remaining Grignard was quenched with 2-propanol (1 mL, 13.1 mmol) and after 10 min of stirring all volatile components were removed under reduced pressure. The residue was redissolved in DCM and filtered through a layer of silica and eluated with DCM until the filtrate was colourless. The solvent was removed under reduced pressure and the remaining yellow liquid was purified by filtration through a layer of silica gel (eluent = pentane followed by DCM) to remove the 2,4,6-tri-iso-propylbenzene. The crude product was further purified via silica gel column chromatography (eluent = hexane:DCM (4:1)). The desired product was isolated as a yellow solid. Yield 267 mg, 71 %.

^{1}H NMR (400 MHz, $CDCl_3$) = 8.40 (dd, *J*=7.5, 1.0 Hz, 1 H), 8.30 (dd, *J*=8.0, 1.5 Hz, 1 H), 8.18 - 8.06 (m, 2 H), 7.77 - 7.63 (m, 3 H), 7.44 - 7.33 (m, 3 H), 7.11 (s, 2 H), 3.00 (sept, *J*=6.9 Hz, 1 H), 2.75 (t, *J*=7.0, 2 H), 2.55 (t, *J*=6.8, 2 H), 2.49 (sept, *J*=6.8 Hz, 2 H), 1.37 (d, *J*=7.0 Hz, 6 H), 1.15 (d, *J*=6.8 Hz, 6 H), 0.99 (d, *J*=6.5 Hz, 6 H);
^{13}C{^{1}H} NMR (101 MHz, $CDCl_3$): δ= 150.9, 150.7, 148.7, 146.4 (br.), 143.0, 142.7, 139.5 (br.), 137.0 (br.), 136.2, 134.9, 133.1, 132.8, 132.0, 131.7, 131.4, 131.3, 128.4, 128.1, 126.7, 126.2, 125.8, 120.4, 35.8, 34.9, 29.7, 29.3, 24.6, 24.5;
^{11}B NMR (128 MHz, $CDCl_3$): δ= 60 (br.);
HR-MS (APCI mode: positive): *m/z* calculated for $[C_{35}H_{38}B]^+$ 469.3061, found 469.3061.

Synthesis of **3**

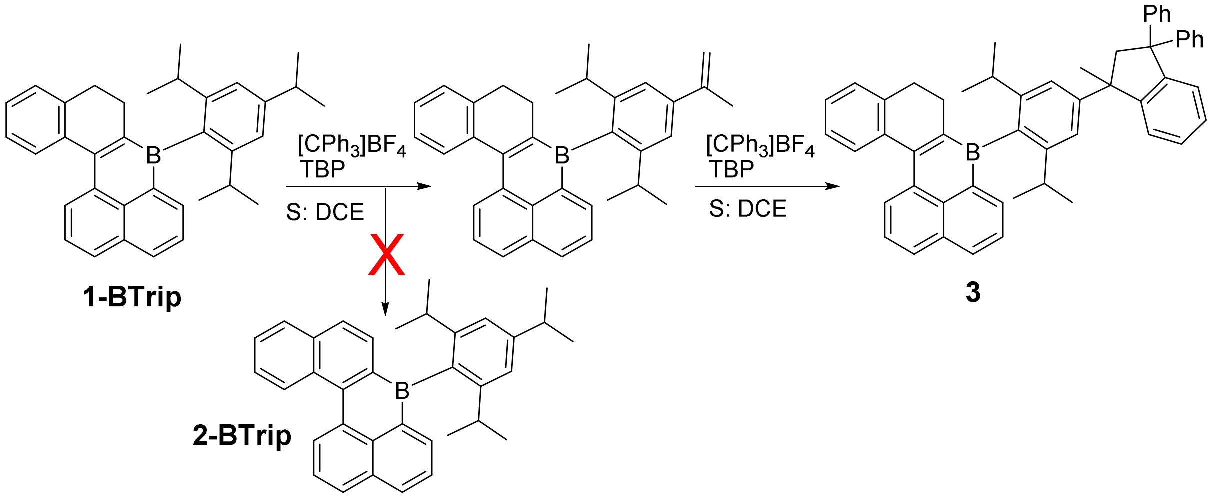

1-BTrip (100 mg, 0.21 mmol), $[Ph_3C]BF_4$ (78 mg, 0.23 mmol) and 2,4,6-tri-tert-butylpyridine (TBP) (54 mg, 0.22 mmol) were dissolved in DCE (3 mL) and the reaction mixture was stirred at overnight at 70°C. After cooling to room temperature the crude reaction mixture was purified via silica gel column chromatography (eluent = hexane:DCM (85:15)). Compound **3** was isolated as a yellow solid. Yield 42 mg, 52%.

^{1}H NMR (400 MHz, CD_2Cl_2) = 8.36 (d, *J*=7.3 Hz, 1 H), 8.30 (dd, *J*=8.0, 1.3, 1 H), 8.10 (d, *J*=8.0 Hz, 1 H), 7.93 (dd, *J*=7.0, 1.3 Hz, 1 H), 7.79 - 7.73 (m, 1 H), 7.70 (t, *J*=7.8 Hz, 1 H), 7.68 - 7.62 (m, 1 H), 7.46 - 7.04 (m, 17 H), 6.89 (d, *J*=1.5 Hz, 1 H), 6.77 - 6.68 (m, 1 H), 3.66 (d, *J*=13.1 Hz, 1 H), 3.20 (d, *J*=13.1 Hz, 1 H), 2.73 (t, *J*=6.9 Hz, 2 H), 2.42 (t, *J*=6.8, 2 H), 2.30 (sept, *J*= 6.6 Hz, 2 H), 1.79 (s, 3 H), 0.98 (d, *J*=6.8 Hz, 3 H), 0.93 (d, *J*=6.8 Hz, 3 H), 0.84 (d, *J*=6.8 Hz, 3 H), 0.73 (d, *J*=6.8 Hz, 3 H);
^{13}C{^{1}H} NMR (101 MHz, $CDCl_3$): δ= 150.7, 150.6, 150.0, 149.8, 149.7, 149.7, 148.3, 146.8, 146.5 (br.), 143.1, 142.7, 138.9 (br.), 136.9 (br.), 136.1, 134.8, 133.0, 132.7, 131.9, 131.6, 131.3, 131.3, 129.5, 129.2, 128.4, 128.1, 128.1, 127.9, 127.6, 127.2, 126.5, 126.4, 126.2, 126.0, 125.8, 120.9, 120.9, 61.2, 61.2, 52.0, 35.7, 35.6, 31.0, 29.7, 29.3, 24.5, 24.5, 24.3;
^{11}B NMR (128 MHz, $CDCl_3$): δ= 60 (br.);
HR-MS (APCI mode: positive): *m/z* calculated for $[C_{54}H_{49}B]^+$ 708.3922, found 708.3928.

Synthesis of **2-BMes**

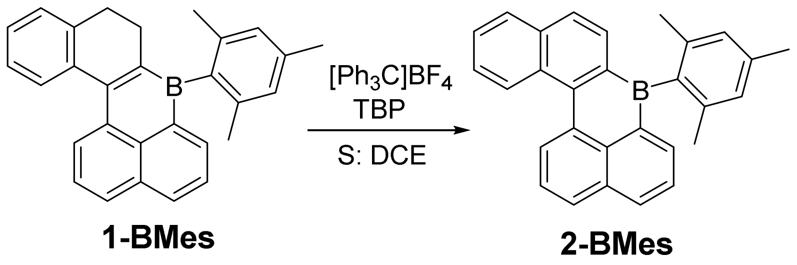

1-BMes (512 mg, 1.33 mmol), 2,4,6-tri-tert-butylpyridine (TBP) (333 mg, 1.33 mmol) and $[Ph_3C]BF_4$ (443 mg, 1.46 mmol) were dissolved in DCE (10 mL) the reaction mixture was stirred for 6 hours at room temperature. TLC examination showed the reaction mixture had not gone to completion. The reaction mixture was then heated overnight at 75°C. Upon cooling the reaction mixture was passed through a plug of silica gel using 20:80 DCM:hexane as eluent and the yellow fractions were collected. The solvent was removed under reduced pressure and the resulting residue was recrystallised from hot methanol and washed with methanol followed by cold pentane to give the desired product as a dark yellow solid. Yield 371 mg, 73 %. Single crystals suitable for X-Ray diffraction were obtained by slow evaporation from acetone.

^{1}H NMR (400 MHz, $CDCl_3$) = 8.84 - 8.74 (m, 1 H), 8.71 (d, *J*=7.3 Hz, 1 H), 8.40 - 8.30 (m, 1 H), 8.30 - 8.21 (m, 1 H), 8.15 (d, *J*=8.0 Hz, 1 H), 7.97 - 8.06 (m, 1 H), 7.93 - 7.79 (m, 3 H), 7.76 (t, *J*=7.4 Hz, 1 H), 7.72 - 7.61 (m, 2 H), 7.05 (s, 2 H), 2.50 (s, 3 H), 2.07 (s, 6 H);
^{13}C{^{1}H} NMR (128 MHz, $CDCl_3$) 143.7, 141.5, 140.2 (br.), 138.9, 137.9, 136.7, 136.0, 134.2 (br.), 133.6, 132.7, 132.6, 132.5, 131.4, 130.6, 130.4, 130.3, 128.3, 127.1, 126.9, 126.7, 126.2, 125.6, 125.1, 23.3, 21.3;
^{11}B NMR (160 MHz, $CDCl_3$): δ= 61 (br.);
HR-MS (HESI mode: positive): *m/z* calculated for $[C_{29}H_{23}B]^+$ 382.1893, found 382.1878.

Synthesis of **2-BTrip**

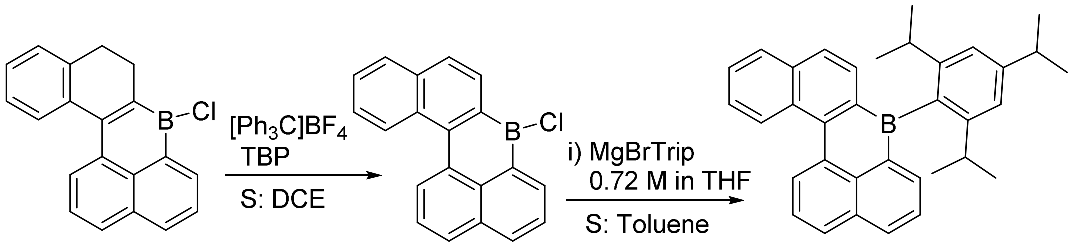

1-BCl **2-BCl** **2-BTrip**

1-BCl (30 mg, 0.1 mmol), $[Ph_3C]BF_4$ (72 mg, 0.2 mmol) and 2,4,6-tri-tert-butylpyridine (TBP) (55 mg, 0.2 mmol) were dissolved in DCE (3 mL) and the reaction mixture was heated for 72 hours at 60°C. Upon cooling to room temperature the solvent was removed under reduced pressure and the resulting residue was suspended in toluene (5 mL). (2,4,6-tri-isopropylphenyl)magnesium bromide (0.72 M in THF) (1.1 mL, 0.8 mmol) was slowly added and the reaction mixture was then stirred for 18 h. The remaining Grignard was quenched with 2-propanol (1 mL, 13.1 mmol) and after 10 min of stirring all volatile components were removed under reduced pressure. The residue was dissolved in pentane and filtered through a layer of silica and eluated with pentane followed by pentane:DCM (1:9) and the yellow fractions were collected. The solvent was removed under reduced pressure and the remaining yellow solid was purified via silica gel column chromatography (eluent = Petroleum ether). The desired product was isolated as a yellow solid. Yield 30 mg, 65 %.

^{1}H NMR (400 MHz, $CDCl_3$) = 8.80 - 8.70 (m, 1 H), 8.67 (d, *J*=7.5 Hz, 1 H), 8.35 (dd, *J*=8.0, 1.0 Hz, 1 H), 8.19 (dd, *J*=6.8, 1.3 Hz, 1 H), 8.15 (d, *J*=7.5 Hz, 1 H), 8.01 -7.93 (m, 1 H), 7.87 - 7.78 (m, 3 H), 7.75 (dd, *J*=7.9, 6.9 Hz, 1 H), 7.68 - 7.58 (m, 2 H), 7.13 (s, 2 H), 3.02 (sept, *J*=6.9 Hz, 1 H), 2.36 (sept, *J*=6.7 Hz, 2 H), 1.38 (d, *J*=7.0 Hz, 6 H), 0.97 (d, *J*=6.5 Hz, 6 H), 0.99 (d, *J*=6.8 Hz, 6 H);

^{13}C{^{1}H} NMR (101 MHz, $CDCl_3$): δ = 151.0, 149.1, 143.6, 142.3, 138.4, 136.4, 134.1, 133.8, 133.2, 132.9, 131.8, 131.1, 130.9, 130.9, 128.9, 127.7, 127.0, 126.6, 126.3, 125.8, 120.5, 36.1, 35.0, 24.6, 24.4, 24.3;

^{11}B NMR (128 MHz, $CDCl_3$): δ= 61 (br.);

HR-MS (APCI mode: positive): *m/z* calculated for $[C_{35}H_{35}B]^{+}$ 466.2826, found 466.2829.

Synthesis of **1-methyl-1,3,3-triphenyl-2,3-dihydro-1H-indene**

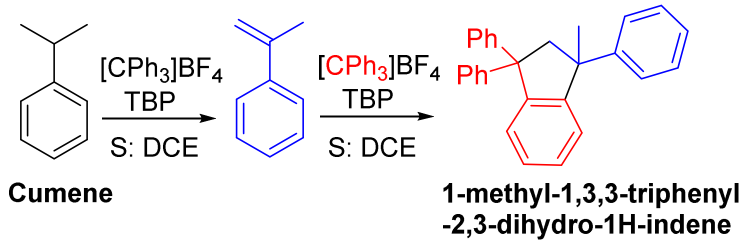

Cumene (12 mg, 0.1 mmol), $[CPh_3]BF_4$ (132 mg, 0.4 mmol) and 2,4,6-tri-tert-butylpyridine (98 mg, 0.4 mmol) was dissolved in DCE (0.8 mL) and was heated at 75°C for 72 hours. After cooling to room temperature the reaction mixture was filtered through a short plug of silica gel and eluted with DCM. The solvent was removed under reduced pressure and the resulting residue was purified by preparative TLC using DCM:Hexane (1:9) as eluent. The desired product was isolated as a white solid. Yield 14 mg, 39 %.

NMR data corresponds with previously published data.[S8]

Synthesis of **6**

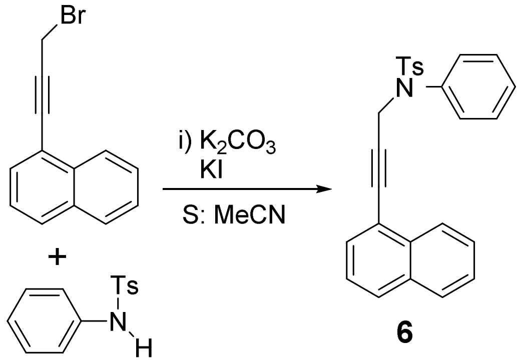

6

6 was prepared according to a modified literature procedure.[S9]

4-Methyl-*N*-phenylbenzenesulfonamide (1.00 g, 4.04 mmol), 1-(3-bromoprop-1-yn-1-yl)naphthalene (991 mg, 4.04 mmol), potassium carbonate (559 mg, 4.04 mmol) and potassium iodide (262 mg, 1.58 mmol) were dissolved in acetonitrile (30 mL). The mixture was refluxed at 90 °C for 16 hours. Water was added and the resulting mixture was extracted with ethyl acetate. The combined organics were dried over sodium sulphate then concentrated *in vacuo* to afford **6** (4-methyl-*N*-(3-(naphthalen-1-yl)prop-2-yn-1-yl)-*N*-phenylbenzenesulfonamide) (1.36 g, 3.30 mmol, 82%) as an orange solid.

^{1}H NMR (400 MHz, CD_2Cl_2): δ= 7.86 – 7.77 (m, 3H), 7.60 (d, *J* = 8.1 Hz, 2H), 7.54 – 7.31 (m, 9H), 7.20 (d, *J* = 8.1 Hz, 2H), 4.82 (s, 2H), 2.32 (s, 3H);

^{13}C{^{1}H} NMR (101 MHz, CD_2Cl_2): δ= 144.5, 140.3, 136.2, 133.6, 131.0, 130.0, 129.6, 129.5, 129.1, 128.7, 128.4, 127.3, 127.0, 126.3, 125.6, 120.4, 88.9, 84.2, 42.7, 21.8;

HR-MS (HESI mode: positive): *m/z* calculated for $[C_{26}H_{21}NO_2S + H]^+$ 412.1366, found 412.1363.

Synthesis of 6-BMes

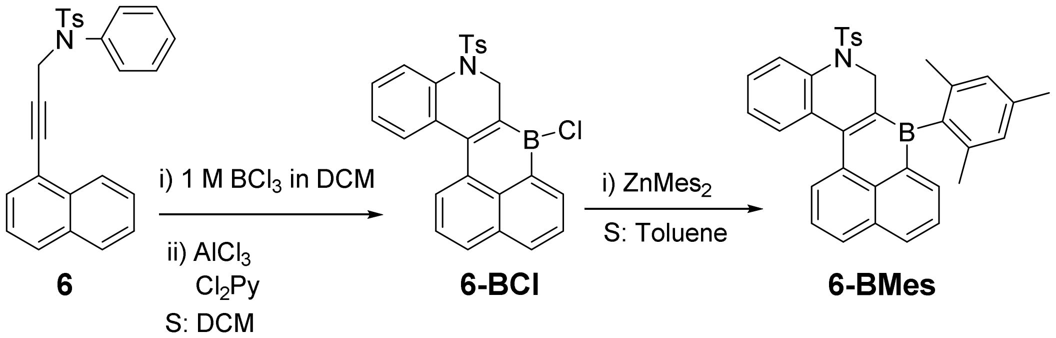

To a solution of **6** (109 mg, 0.265 mmol) in dichloromethane (5 mL) was added boron trichloride solution (1.0 M in dichloromethane, 0.29 mL, 0.29 mmol) and the mixture was left to stir for 10 minutes. The reaction mixture was dried *in vacuo* then dissolved in dichloromethane (5 mL). 2,6-Dichloropyridine (88 mg, 0.595 mmol, 2.2 eq.) and aluminium trichloride (81 mg, 0.608 mmol, 2.3 eq.) were added to the solution and the reaction mixture was stirred at 60 °C for 50 minutes. The mixture dried *in vacuo* then extracted in toluene (4 x 8 mL) and filtered to give an orange solution. To this was added dimesitylzinc (89 mg, 0.29 mmol) and the reaction mixture was left to stir at ambient temperature for three hours. The mixture was filtered through a silica plug and washed with toluene. The product was purified by flash column chromatography using ethyl acetate: hexane (20:80) eluent to yield the product at Rf = 0.41. Crystals were obtained by slow evaporation of a saturated hexane solution at ambient temperature (76.9 mg, 0.143 mmol, 54%).

^{1}H NMR (CD_2Cl_2, 400 MHz) δ: 8.28 (dd, *J*=8.0, 1.5 Hz, 1H), 8.11 – 8.03 (m, 2H), 7.88 – 7.82 (m, 1H), 7.76 – 7.65 (m, 2H), 7.63 – 7.56 (m, 1H), 7.56 – 7.50 (m, 2H), 7.42 (ddd, 7.4, 6.8, 1.4 Hz, 1H), 7.13 (d, 8.2 Hz, 2H), 6.95 (s, 2H), 6.54 (d, 8.0 Hz, 2H), 4.58 (s, br, $\nu_{1/2}$ = 144 Hz, 2H), 2.72 (s, 3H), 2.04 (s, br, $\nu_{1/2}$ = 15.7 Hz, 6H), 1.81 (s, 3H).

^{11}B{^{1}H} NMR (CD_2Cl_2, 128 MHz) δ**:** 55.0.

^{13}C{^{1}H} NMR (CD_2Cl_2, 101MHz) δ: 148.9, 143.6, 143.0, 140.1, 138.9, 137.6, 136.8, 136.3, 134.1, 132.6, 132.5, 132.5, 131.5, 131.1, 129.8, 129.8, 129.1, 128.6, 128.0, 127.7, 127.0, 126.8, 125.4, 51.1, 23.4, 21.6, 21.2.

HR-MS (HESI mode: positive): *m/z* calculated for $[C_{35}H_{30}BNO_2S+H]^+$ 540.2163, found 540.2162.

Synthesis of 7-BMes

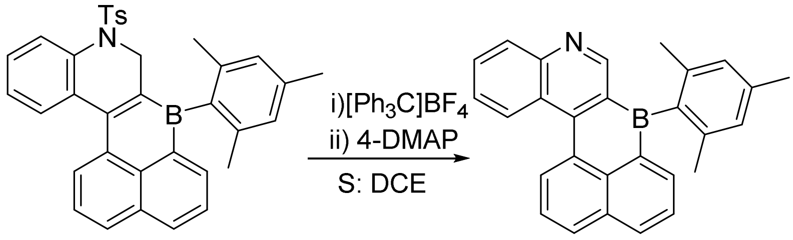

6-BMes **7-BMes**

6-BMes (42.2 mg, 7.82 x 10^{-5} mol) and $[Ph_3C][BF_4]$ (35 mg, 1.1 x 10^{-4} mol, 1.4 eq.) were dissolved in DCE (0.6 mL) to give a bright orange solution which was stirred at ambient temperature for 17 hours. To the solution was added 4-DMAP (18 mg, 1.5 x 10^{-5} mol, 1.9 eq.) and the mixture became bright yellow. All volatiles were removed *in vacuo* to give a yellow solid. The product was purified by flash column chromatography using acetone: hexane (15:85) eluent to yield the product at Rf = 0.34. The product was washed with methanol then pentane to furnish **7-BMes** as a yellow powder (23.6 mg, 6.16 x 10^{-5} mol, 79%). Crystals were obtained by slow evaporation of a saturated dichloromethane/ethyl acetate solution at ambient temperature.

^{1}H NMR (CD_2Cl_2, 400 MHz) δ: 9.06 (s, 1H), 8.80 (d, 7.4 Hz, 1H), 8.68 (d, 8.5 Hz, 1H), 8.39 (dd, 8.1, 1.2 Hz, 1H), 8.26 (dd, 8.0Hz, 1.2 Hz, 1H), 8.21 (m, 2H), 7.88 (dd, 7.8, 7.8 Hz, 1H), 7.84 (ddd, 8.3, 6.9, 1.2 Hz, 1H), 7.77 (dd, 8.0, 6.9 Hz, 1H), 7.71 (ddd, 8.3, 6.9, 1.3 Hz, 1H), 6.98 (s, 2H), 2.40 (s, 3H), 1.97 (s, 6H).

^{11}B{^{1}H} NMR (CD_2Cl_2, 128 MHz) δ: 59.5.

^{13}C{^{1}H} NMR (CD_2Cl_2, 101MHz) δ: 157.8, 152.4, 150.4, 142.7, 139.4, 137.8, 137.2, 135.6, 133.3, 133.2, 132.6, 130.9, 130.7, 130.6, 130.3, 127.6, 127.2, 126.9, 126.0, 125.7, 23.8, 21.6.

HR-MS (HESI mode: positive): *m/z* calculated for $[C_{28}H_{22}BN+H]^+$ 384.1918, found 384.1906.

Synthesis of **8**

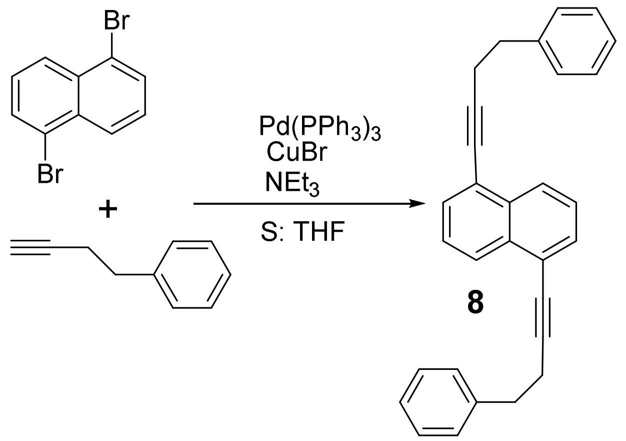

1,5-dibromonaphthalene (0.99 g, 3.46 mmol), 3-butyn-1-yl-benzene (1.02 g, 7.83 mmol) and NEt_3 (9 mL) was added to a solution of $Pd(PPh_3)_4$ (82 mg, 0.07 mmol) and CuBr (28 mg, 0.19 mmol) in THF (5 mL). The reaction mixture was stirred and heated at 60°C for 18 hours. After cooling to room temperature the reaction mixture was filtered through a short plug of silica topped with celite. The resulting black solution was evaporated to dryness and the purified via silca gel column chromatography (eluent = hexane:DCM 85:15) to give the desired product as colourless solid. Yield 0.91 mg, 68 %.

^{1}H NMR (400 MHz, $CDCl_3$): δ= 8.11 (d, *J*=8.6 Hz, 1 H), 7.61 (d, *J*=7.1 Hz, 1 H), 7.45 - 7.33 (m, 5 H), 7.32 - 7.27 (m, 1 H), 3.04 (t, *J*=7.3 Hz, 2 H), 2.89 (t, *J*=7.3 Hz, 2 H);
^{13}C{^{1}H} NMR (101 MHz, $CDCl_3$): δ= 140.6, 133.2, 130.3, 128.6, 128.4, 128.4, 126.4, 126.4, 125.7, 121.8, 94.6, 79.3, 35.1, 21.9;

HR-MS (APCI mode: positive): *m/z* calculated for $[C_{30}H_{24} + H]^+$ 385.1951, found 385.1942.

Synthesis of **9-BCl**

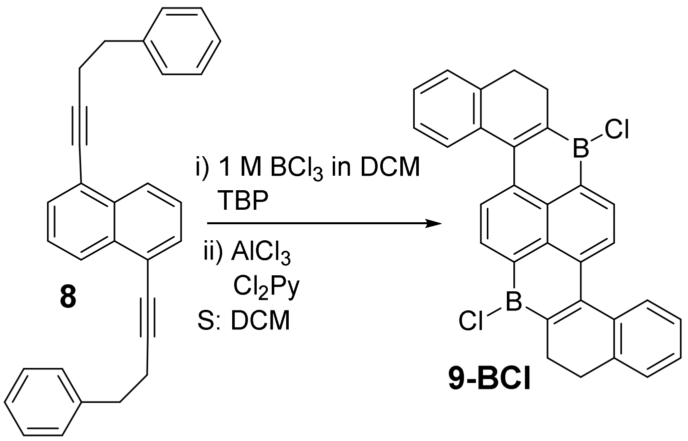

BCl_3 (1M in DCM) (2.30 mL, 2.30 mmol) was added to a solution of **8** (203 mg, 0.53 mmol) and 2,4,6-tri-tert-butylpyridine (262 mg, 1.06 mmol) in DCM (4 mL). The colour of the solution changed instantly from pale yellow to bronze. After 5 min of stirring $AlCl_3$ (155 mg, 1.16 mmol) was added to the reaction mixture. After stirring for 15 minutes a colour change from bronze to purple was observed and the reaction mixture was evaporated to dryness under reduced pressure (to remove BCl_3) and the resulting residue was redissolved in DCM (3 mL). 2,6-dichloropyridine (157 mg, 1.06 mmol) and $AlCl_3$ (155 mg, 1.16 mmol) were then added to the reaction mixture. The reaction mixture was then stirred until all solids had dissolved (~2 minutes) then the stirring was stopped. The product started to precipitate almost instantly and the mixture was allowed to stand for 18 hours. Single crystals suitable for X-Ray diffraction were obtained directly from this precipitation. The reaction mixture was filtered using a filter cannula and the remaining solid was washed two times with 2 mL dichloromethane. The solid was dried under reduced pressure to obtain the desired product as a purple powder. Yield 215 mg, 86%. The sample proved to be poorly soluble in benzene, DCM, ortho-dichlorobenzene.

^{1}H NMR (400 MHz, C_6D_6): δ = 8.41 (d, *J*=7.5 Hz, 2H), 7.90 (d, *J*=7.5 Hz, 2H), 7.41 - 7.34 (m, 2H), 7.22 - 7.08 (m, 6H under C_6D_5H signal), 2.67 (br., 4 H), 2.50 (t, *J*=7.0 Hz, 4H);
^{13}C{^{1}H} NMR (101 MHz, C_6D_6) could not be recorded due to the low solubility of the compound.
^{11}B NMR (128 MHz, C_6D_6) could not be recorded due to the low solubility of the compound.
Elemental Analysis = Calculated: **C** = 76.18%, **H** = 4.26%; Found: **C** = 75.71%, **H** = 4.28%

Synthesis of **9-BMes**

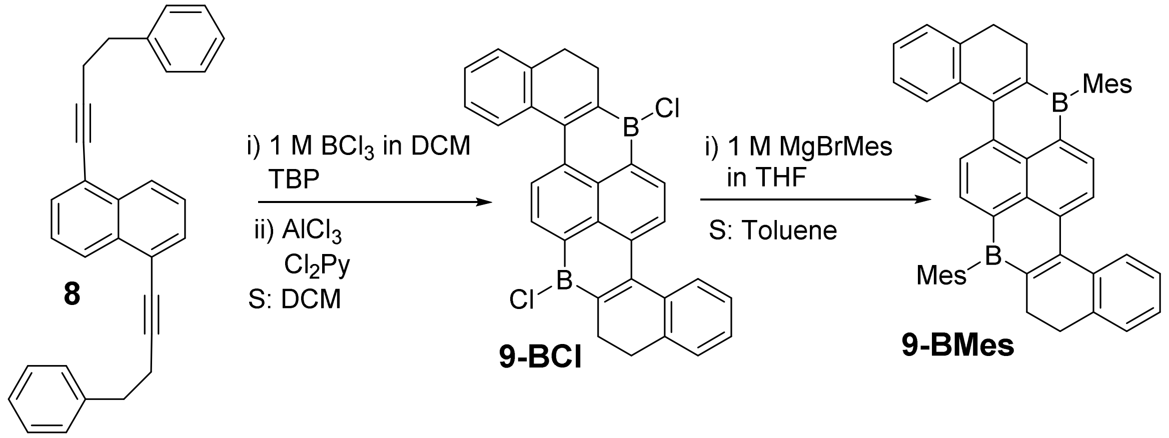

BCl_3 (1M in DCM) (1.21 mL, 1.21 mmol) was added to a solution of **8** (100 mg, 0.26 mmol) and 2,4,6-tri-tert-butylpyridine (129 mg, 0.52 mmol) in DCM (5 mL). The solution changed colour instantly from pale yellow to bronze. After 5 min of stirring $AlCl_3$ (69 mg, 52 mmol) was added and the reaction mixture stirred for 10 min. All volatiles were then removed under reduced pressure (to remove excess BCl_3) and the resulting residue was redissolved in DCM (10 mL). 2,6-Dichloropyridine (76 mg, 0.52 mmol) and $AlCl_3$ (69 mg, 0.52 mmol) were then added to the reaction mixture. The reaction mixture was stirred for 30 min and the reaction mixture was evaporated to dryness under reduced pressure and the resulting residue containing **9-BCl** was suspended in toluene. Mesitylmagnesium bromide (1 M in THF) (3.12 mL, 3.12 mmol) was then slowly added and the reaction mixture was stirred for 30 minutes. The excess MgBrMes was quenched with a mixture of IPA: toluene (1:9) (10 mL) and after 10 minutes of stirring all volatiles were removed under reduced pressure. The residue was redissolved in DCM, filtered through a plug of silica gel and eluted until the filtrate was colourless. The crude product was further purified by silica gel column chromatography (eluent = hexane:DCM 4:1). The desired product was isolated as a red solid. Slow evaporation of the chromatography fractions gave single crystals suitable for X-Ray diffraction. During column chromatography the compound undergoes protodeboronation resulting in an extremely low isolated yield. Furthermore, on standing in wet $CDCl_3$ **9-BMes** decomposes completely over 24 h.

^{1}H NMR (400 MHz, $CDCl_3$): δ= 8.11 (d, J=7.5 Hz, 2 H), 7.92 (d, J=7.3 Hz, 2 H), 7.67 - 7.56 (m, 2 H), 7.44 - 7.32 (m, 6 H), 6.96 (s, 4 H), 2.75 (t, J=7.0 Hz, 4 H), 2.50 - 2.35 (overlapped m and s, 10 (4+6) H), 2.15 (s, 12 H);
^{13}C{^{1}H} NMR (101 MHz, $CDCl_3$): δ= 153.0, 147.5, 141.7, 140.3, 138.9, 138.2, 136.7, 133.7, 131.8, 131.7, 129.8, 128.3, 127.6, 126.9, 125.6, 29.0, 27.5, 22.8, 21.3;
^{11}B NMR (128 MHz, $CDCl_3$): δ= 63 (br.);
HR-MS (APCI mode: positive): m/z calculated for $[C_{48}H_{42}B_2 + H]^+$ 641.3545, found 641.3534.

Synthesis of **9-BTrip**

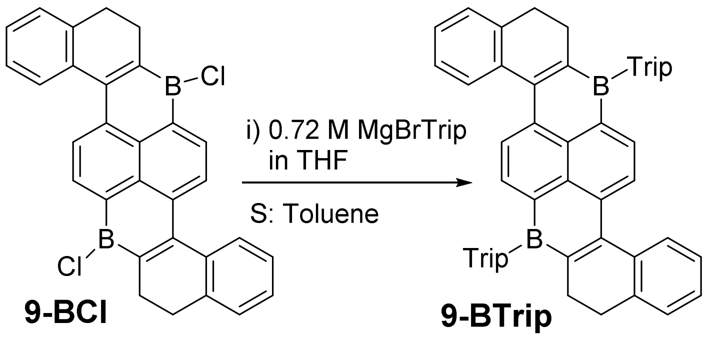

9-BCl (100 mg, 0.21 mmol) was suspended in DCM (2 mL) and (2,4,6-tri-isopropylphenyl)magnesium bromide (0.72 M in THF) (0.62 mL, 0.72 mmol) was added over a period of 5 min. The purple suspension was stirred for 16 hours and the solution changed colour to red. Additional (2,4,6-tri-isopropylphenyl)magnesium bromide (0.72 M in THF) (0.62 mL, 0.72 mmol) was slowly added and the suspension turned a dark red colour and the reaction mixture was stirred at room temperature for a further 6 hours. The excess MgBrTrip was then quenched with a mixture of IPA: toluene (1:9) (10 mL). The suspension was filtered through a plug of base treated (5% NEt_3 in hexane) silica gel and eluted with DCM until the filtrate was colourless. All volatiles were removed under reduced pressure giving a red powder. The crude product was purified using silica gel column chromatography (eluent = hexane:DCM 4:1). The desired product was obtained as a red powder. Yield 120 mg, 70 %.

^{1}H NMR (400 MHz, CD_2Cl_2): δ= 8.05 - 8.17 (m, *J*=7.5 Hz, 2 H), 7.93 - 7.83 (m, *J*=7.5 Hz, 2 H), 7.64 - 7.56 (m, 2 H), 7.40 - 7.29 (m, 6 H), 7.08 (s, 4 H), 2.96 (sept, *J*=6.9 2 H), 2.71 (t, *J*=7.1 Hz 4 H), 2.53 (sept, *J*=7.6 Hz, 4 H), 2.45 (t, *J*=6.7 Hz, 4 H), 1.33 (d, *J*=6.8 Hz, 12 H), 1.17 (d, *J*=6.8 Hz, 12 H), 1.05 (d, *J*=6.5 Hz, 12 H);
^{13}C{^{1}H} NMR (101 MHz, CD_2Cl_2): δ= 152.3, 150.6, 149.0, 142.5, 141.2, 139.1, 134.2, 132.1, 131.9, 130.4, 128.8, 128.1, 126.1, 120.5, 36.0, 34.9, 29.4, 28.9, 24.6, 24.6, 24.5;
^{11}B NMR (128 MHz, CD_2Cl_2): δ= 62 (br.);
HR-MS (APCI mode: positive): *m/z* calc. for $[C_{60}H_{66}B_2 + H]^+$ 809.5423, found 809.5429.

Synthesis of **9-BTrip**

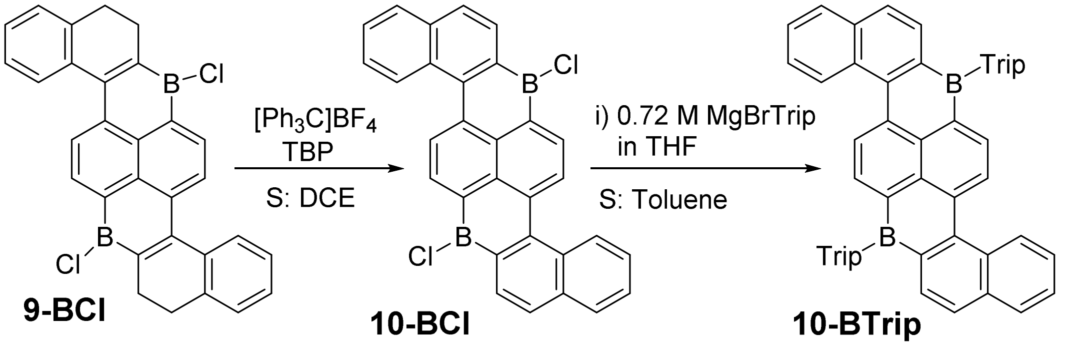

9-BCl (55 mg, 0.12 mmol), 2,4,6-tri-tert-butylpyridine (TBP) (117 mg, 0.47 mmol) and $[Ph_3C]BF_4$ (154 mg, 0.47 mmol) were dissolved in DCE (10 mL) and the reaction mixture was stirred for 120 hours at 75°C. Upon cooling the reaction mixture was evaporated to dryness under reduced pressure and the resulting residue was suspended in toluene (10 mL). (2,4,6-Tri-isopropylphenyl)magnesium bromide (0.72 M in THF) (1.22 mL, 0.88 mmol) then was added over a period of 5 minutes. The reaction mixture was then stirred overnight and the excess MgBrTrip was quenched with a mixture of IPA:toluene (1:9) (10 mL) and after 1 hour of stirring all volatiles were removed under reduced pressure. The residue was dissolved in DCM:hexane (2:8) and filtered through a layer of silica gel and eluted with hexane followed by DCM:hexane (2:8) retaining only the coloured fraction. The resulting solution was evaporated to dryness under reduced pressure and the resulting residue was purified by preparative TLC using pentane followed by pentane:acetone (98:2) as eluent collecting the orange/red band. The desired product was extracted from the silica using DCM and the resulting solution was evaporated to dryness under reduced pressure. The resulting red/orange residue was recrystallised from acetone. The resulting solid was then collected by filtration and washed with acetone followed by pentane to give the desired product as a red/orange powder. Yield 42 mg, 45%. Single crystals suitable for X-Ray diffraction were obtained by slow evaporation from DCM.

^{1}H NMR (400 MHz, $CDCl_3$): δ= 8.64 (d, *J*=8.3 Hz, 2 H), 8.40 (d, *J*=7.3 Hz, 2 H), 8.28 (d, *J*=7.3 Hz, 2 H), 7.96 - 7.91 (m, 2 H), 7.91 - 7.84 (m, *J*=8.0 Hz, 2 H), 7.75 - 7.84 (m, *J*=8.0 Hz, 2 H), 7.65 - 7.54 (m, 4 H), 7.16 (s, 4 H), 3.06 (sept, *J*=6.9 Hz, 2 H), 2.53 (sept, *J*=6.7 Hz, 4 H), 1.42 (d, *J*=7.0 Hz, 12 H), 1.08 (d, J=8.5 Hz, 12 H), 1.10 (d, *J*=8.5 Hz, 12 H);
^{13}C{^{1}H} NMR (101 MHz, $CDCl_3$): δ = 150.3, 148.5, 144.1, 140.0, 139.7, 138.3 (br.), 138.1, 137.6 (br.), 136.5 (br.), 133.6, 132.7, 132.0, 131.0, 129.5, 128.6, 127.5, 127.3, 126.0, 120.0, 35.6, 34.3, 24.3, 24.2, 24.2;
^{11}B NMR (128 MHz, $CDCl_3$): δ= 62 (br.);
MALDI-TOF: calc. for $C_{60}H_{62}B_2^+$ $[M]^+$ = 804.5, found 804.9

Synthesis of **5**

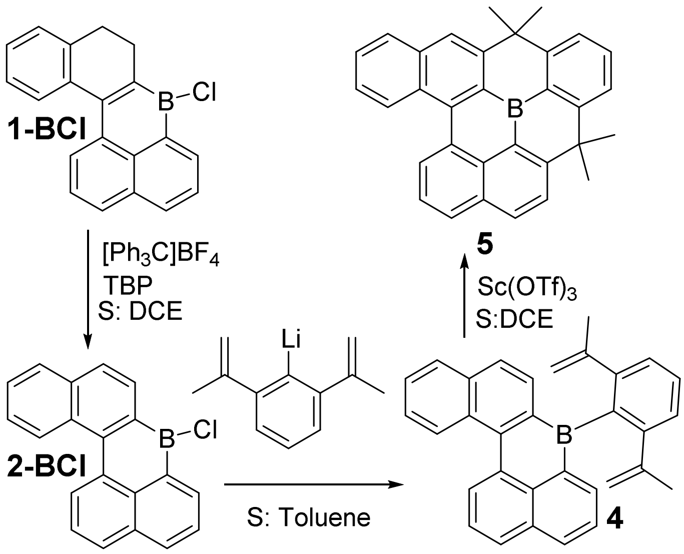

1-BCl (84 mg, 0.28 mmol), $[Ph_3C]BF_4$ (184 mg, 0.56 mmol) and 2,4,6-tri-tert-butylpyridine (TBP) (140 mg, 0.56 mmol) were dissolved in DCE (10 mL) and the reaction mixture was heated for 18 hours at 75°C to form **2-BCl**. Upon cooling to room temperature the solvent was removed under reduced pressure and the resulting residue was dissolved in toluene (10 mL). A solution of (2,6-di(prop-1-en-2-yl)phenyl)lithium was prepared by adding ^{n}BuLi (1.6 M in hexanes) (0.77 mL, 1.23 mmol) dropwise to stirred solution of 2-bromo-1,3-di(prop-1-en-2-yl)benzene (264 mg, 1.12 mmol) in toluene (3 mL) at 0°C, this solution was then stirred for 6 hours at room temperature. The solution of (2,6-di(prop-1-en-2-yl)phenyl)lithium in toluene was added dropwise to the stirred solution of **2-BCl** in toluene at room temperature and was stirred overnight. A solution of IPA:toluene (1:9) (10 mL) was added dropwise to the reaction mixture which was then evaporated to dryness under reduced pressure to give a yellow residue. Attempts to isolated **4** using silica gel chromatography were unsuccessful so the impure reaction residue was dissolved in DCE (3.5 mL) and $Sc(OTf)_3$ (117 mg, 0.24 mmol) (which had been heated under vacuum at 180°C for 10 min) was added. The reaction mixture was then heated at 75°C for 16 hours. Upon cooling to room temperature was passed through a short plug of silica gel and eluted with DCM. The solvent was removed under reduced pressure and the resulting residue was purified by base treated (5% NEt_3 in hexane) silica gel chromatography [eluent: DCM:Petroleum ether (1:9)]. The desired product was then isolated as a yellow powder. Yield 31 mg, 26%. In our hands, single crystals suitable for X-Ray diffraction could not be obtained.

^{1}H NMR (400 MHz, $CDCl_3$): δ= 8.82 (d, *J*=7.5, 1 H), 8.79 (d, *J*=7.5 Hz, 1 H), 8.40 (d, *J*=8.5 Hz, 1 H), 8.24 (s, 1 H), 8.16 - 8.03 (m, 3 H), 7.82 (t, *J*=7.8 Hz, 1 H), 7.78 - 7.71 (m, 3 H), 7.67 - 7.58 (m, 2 H), 1.95 (s, 6 H), 1.88 ppm (s, 6 H);
^{13}C{^{1}H} NMR (101 MHz, $CDCl_3$): δ= 159.8, 156.9, 156.4, 152.3, 142.2, 138.5, 135.2, 134.1, 133.1, 132.0, 131.3, 129.7, 129.6, 129.5, 129.3, 127.2, 126.0, 125.9, 125.7, 125.1, 124.8, 124.1, 44.0, 43.4, 35.3, 33.1;

^{11}B NMR (128 MHz, $CDCl_3$): δ= 45 (br.);
HR-MS (APCI mode: positive): *m/z* calc. for $[C_{32}H_{25}B + H]^+$ 421.2122, found 421.2120.

Synthesis of **11**

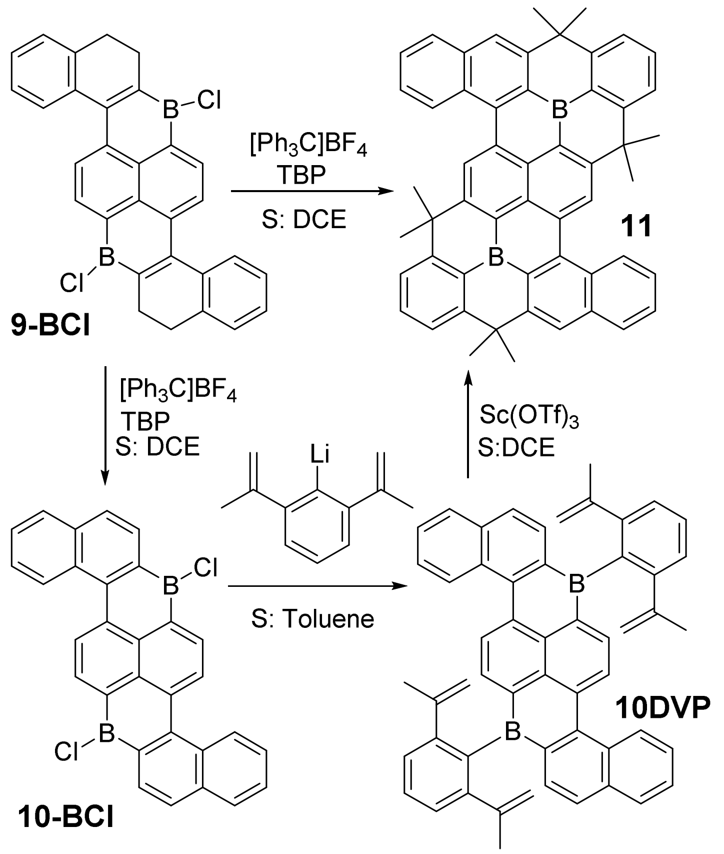

9-BCl (75 mg, 0.16 mmol), $[Ph_3C]BF_4$ (212 mg, 0.64 mmol) and 2,4,6-tri-tert-butylpyridine (TBP) (160 mg, 0.64 mmol) were dissolved in DCE (12 mL) and the reaction mixture was heated for 5 days at 75°C to form **10-BCl**. Upon cooling to room temperature the solvent was removed under reduced pressure and the resulting residue was suspended in toluene (15 mL). A solution of (2,6-di(prop-1-en-2-yl)phenyl)lithium was prepared by adding ^{n}BuLi (1.6 M in hexanes) (0.85 mL, 1.36 mmol) dropwise to stirred solution of 2-bromo-1,3-di(prop-1-en-2-yl)benzene (294 mg, 1.28 mmol) in toluene (3 mL) at 0°C, this solution was then stirred for 6 hours at room temperature. The solution of (2,6-di(prop-1-en-2-yl)phenyl)lithium in toluene was added dropwise to the stirred solution of **10-BCl** in toluene at room temperature and was stirred overnight. A solution of IPA:toluene (1:9) (10 mL) was added dropwise to the reaction mixture which was then evaporated to dryness under reduced pressure to give a yellow residue. Attempts to isolated **10DVP** using silica gel chromatography were unsuccessful in addition to **10DVP** decomposing on silica gel. The impure reaction residue was then dissolved in DCE (10 mL) and $Sc(OTf)_3$ (610 mg, 1.24 mmol) (which had been heated under vacuum at 180°C for 10 min) was added. The reaction mixture was then heated at 75°C for 16 hours. Upon cooling to room temperature was passed through a short plug of silica gel and eluted with DCM. The solvent was removed under reduced pressure and the resulting residue was purified by silica gel chromatography

[eluent: DCM:Petroleum ether (1:9)]. The desired product was then isolated as a red powder. Yield 12 mg, 11%. Slow evaporation of the chromatography fractions gave single crystals suitable for X-Ray diffraction. Compound **11** proved to be poorly soluble in a range on common organic solvents.

^{1}H NMR (400 MHz, $CDCl_3$): δ= 9.06 (s, 2 H), 8.90 - 8.82 (m, 2 H), 8.30 (s, 2 H), 8.16 - 8.09 (m, 2 H), 7.85 - 7.76 (m, 6 H), 7.74 - 7.66 (m, 4 H), 1.99 (d, *J*=5.0 Hz, 12 H), 1.98 ppm (d, *J*=5.0 Hz, 12 H);

^{13}C{^{1}H} NMR (101 MHz, C_6D_6) could not be recorded due to the low solubility of the compound.

^{11}B NMR (128 MHz, C_6D_6) could not be recorded due to the low solubility of the compound.

MALDI-TOF: calc. for $C_{54}H_{42}B_2^+$ $[M]^+$ = 712.3, found 712.6

NMR Spectra

1-BCl

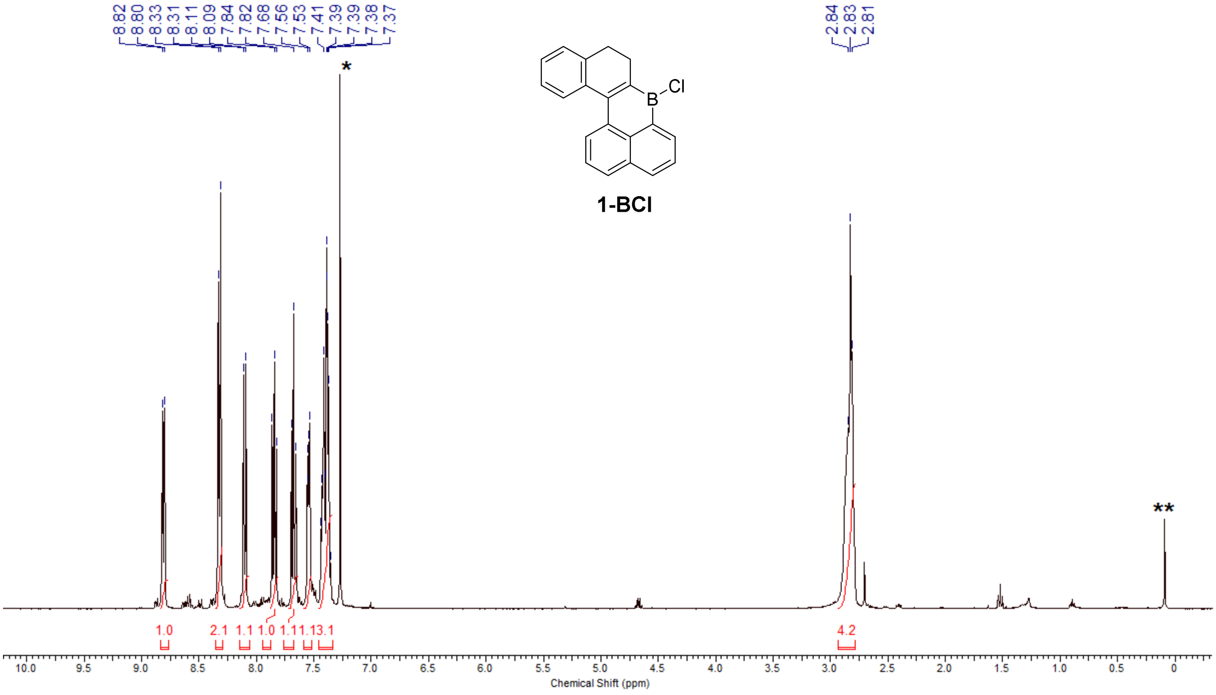

^{1}H NMR spectra of **1-BCl** in $CDCl_3$. * = $CHCl_3$, ** = silicon grease.

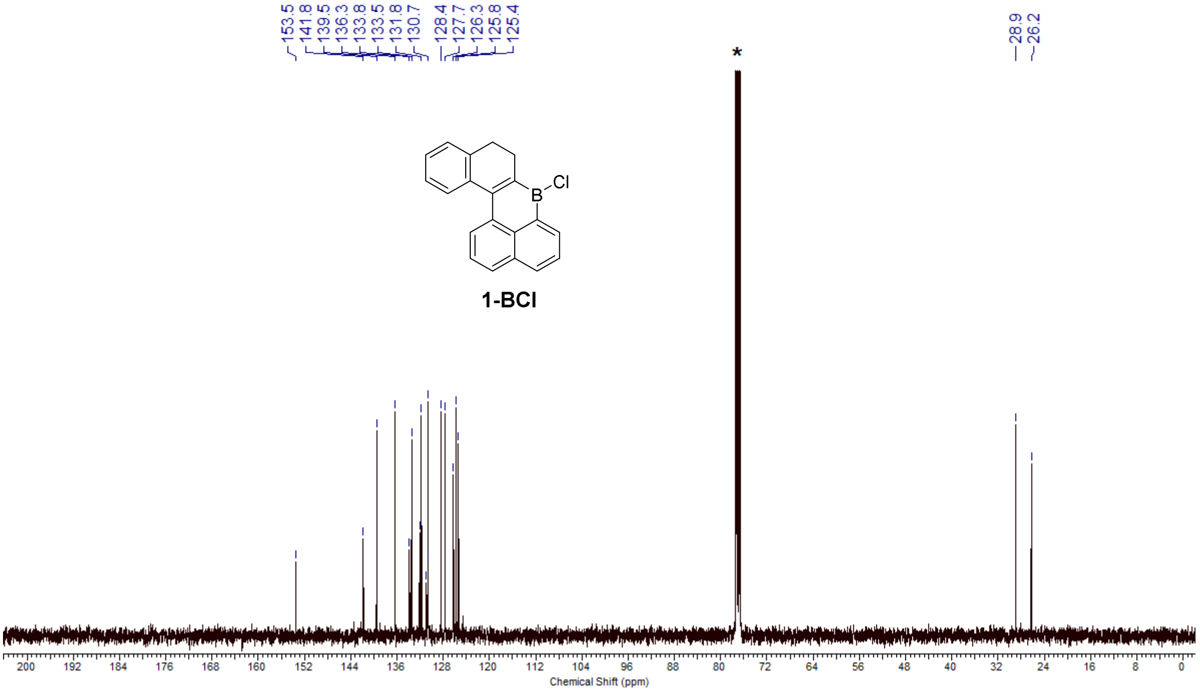

^{13}C{^{1}H} NMR spectra of **1-BCls** in $CDCl_3$. * = $CDCl_3$.

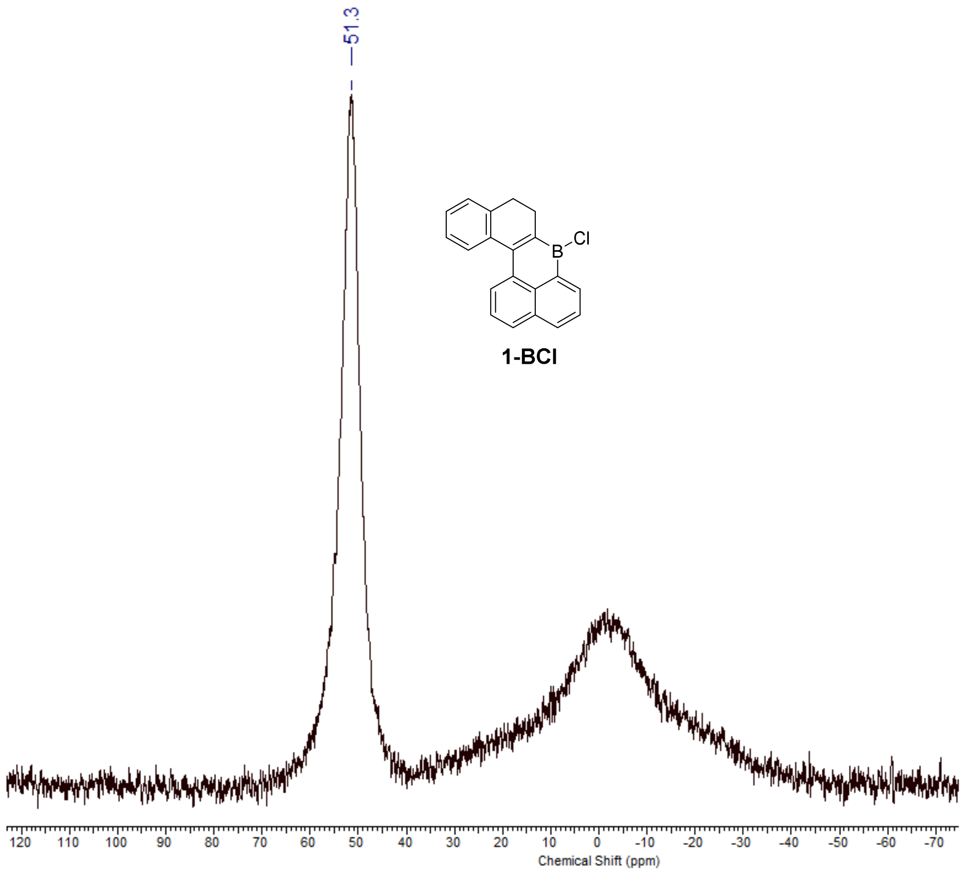

^{11}B NMR spectra of **1-BCl** in $CDCl_3$.

1-BMes

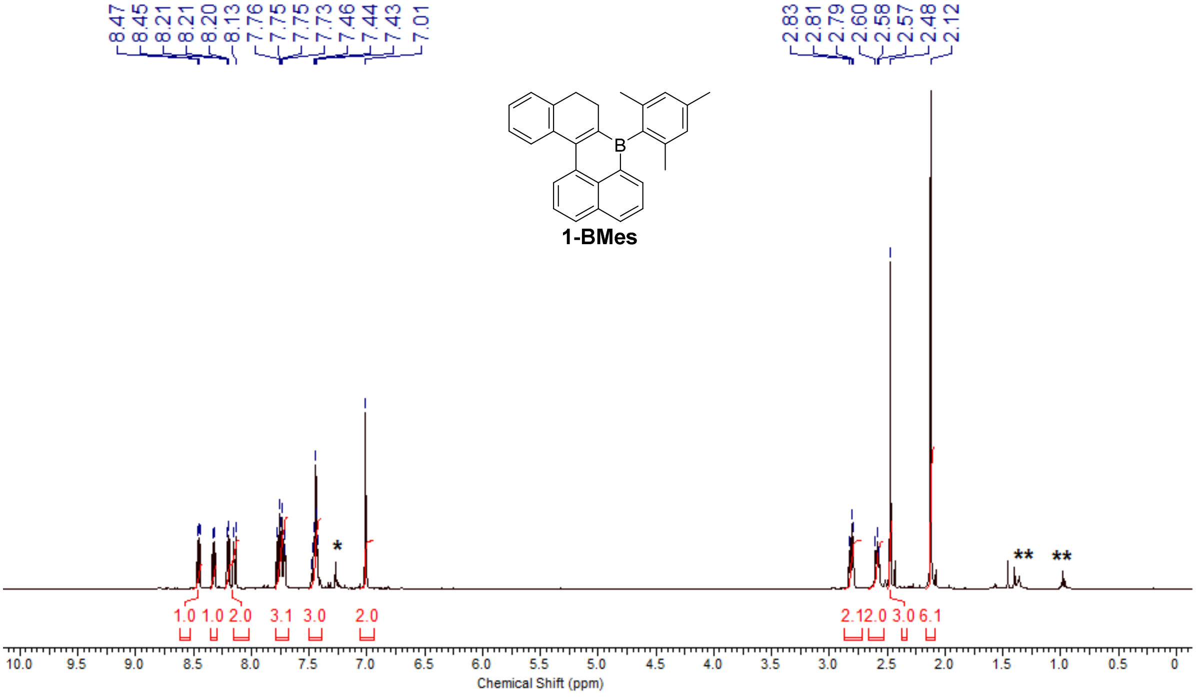

^{1}H NMR spectra of **1-BMes** in $CDCl_3$. * = $CHCl_3$, ** = pentane.

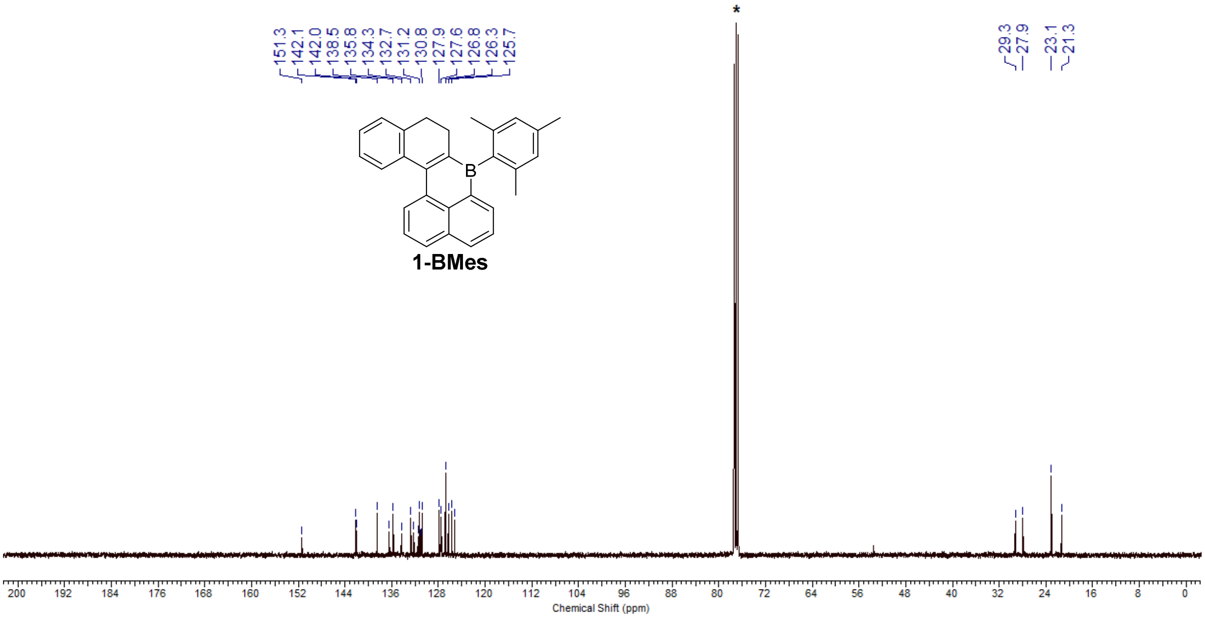

^{13}C{^{1}H} NMR spectra of **1-BMes** in $CDCl_3$. * = $CDCl_3$.

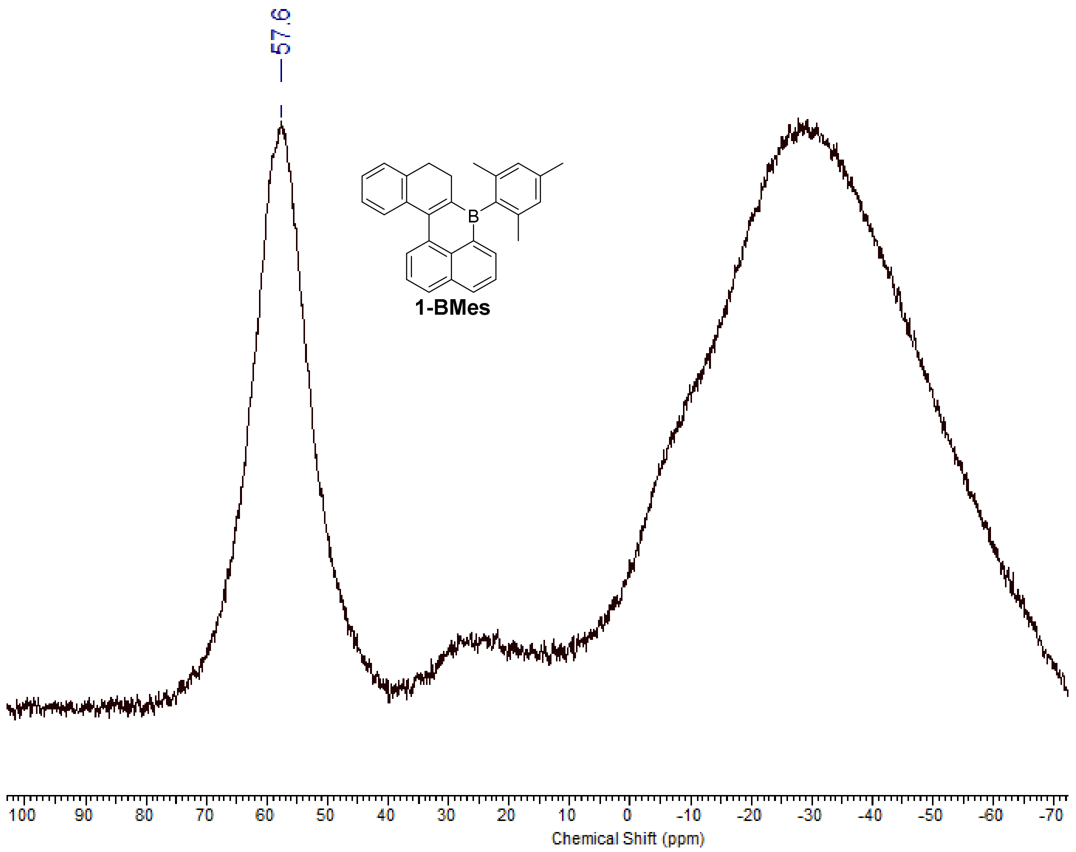

^{11}B NMR spectra of **1-BMes** in $CDCl_3$.

2-BMes

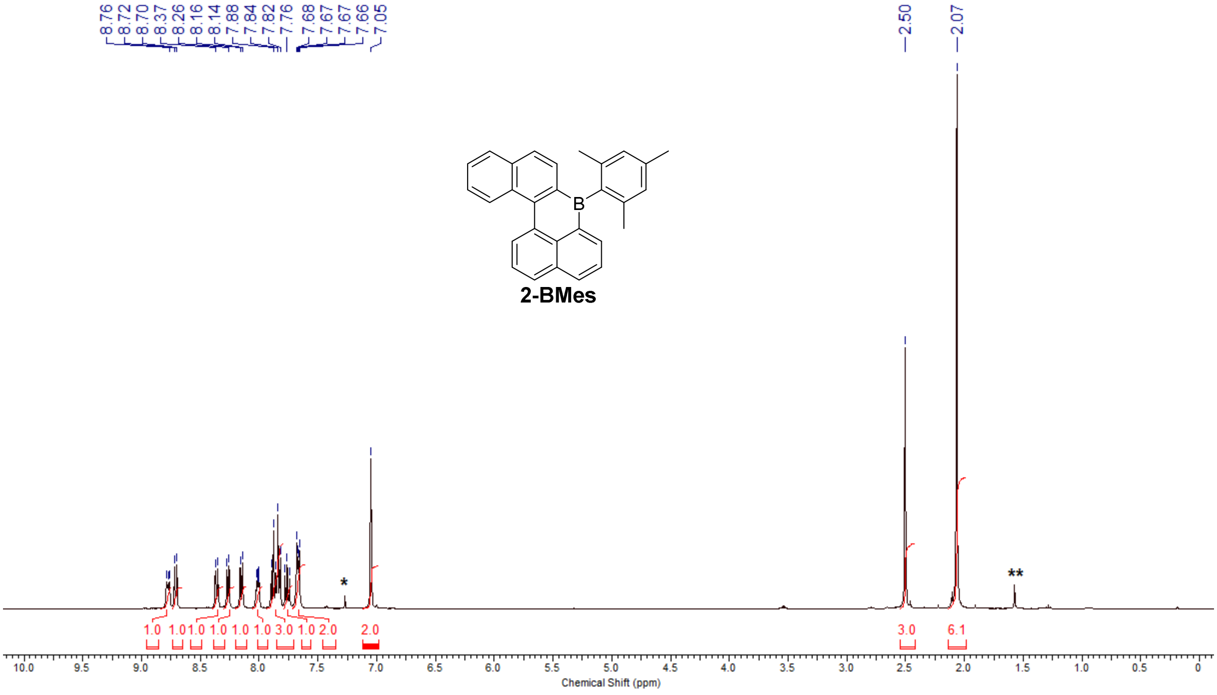

^1H NMR spectra of **2-BMes** in $CDCl_3$. * = $CHCl_3$, ** = H_2O.

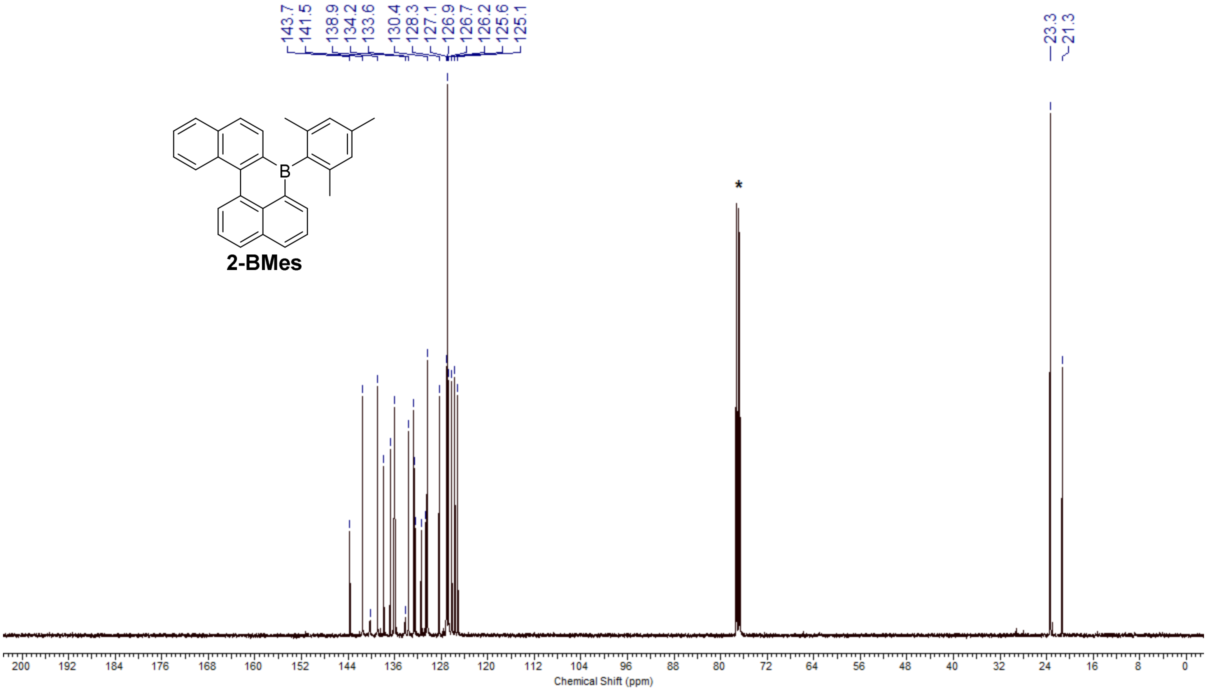

$^{13}C\{^1H\}$ NMR spectra of **2-BMes** in $CDCl_3$. * = $CDCl_3$.

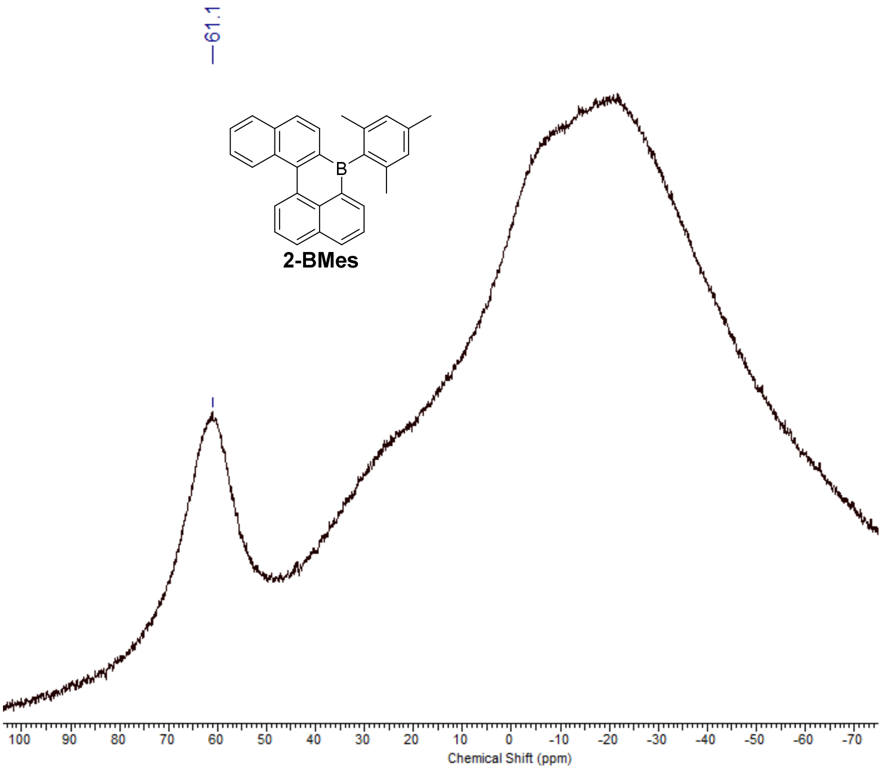

^{11}B NMR spectra of **2-BMes** in $CDCl_3$.

1-BTrip

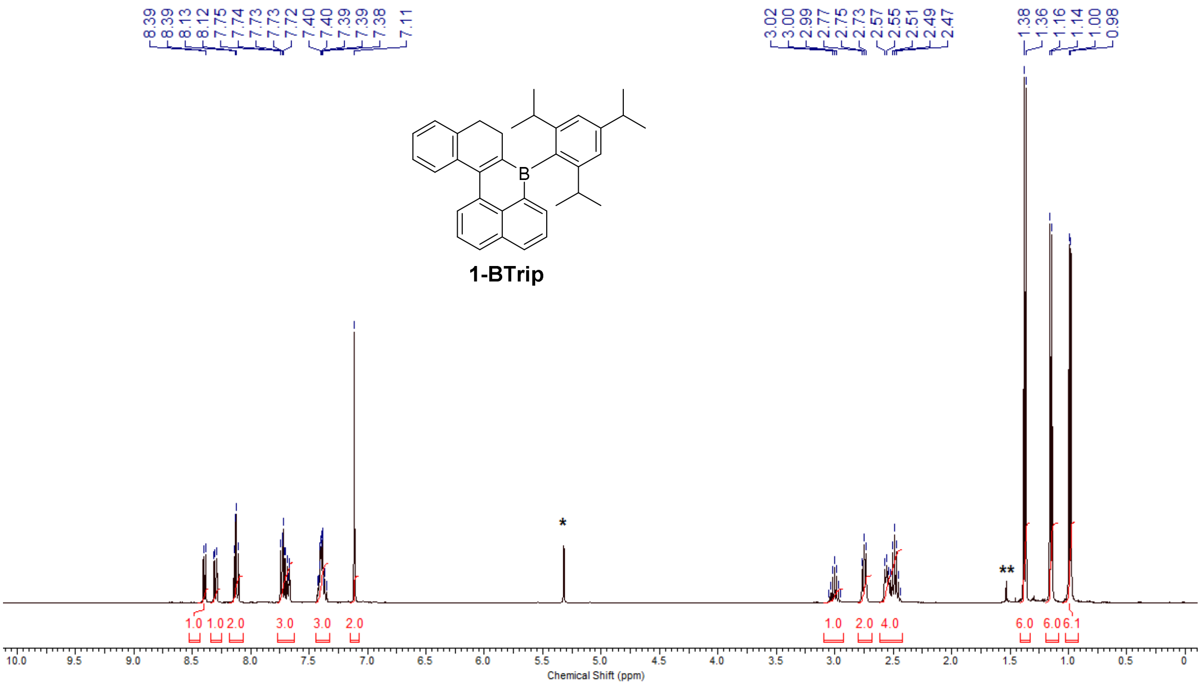

^{1}H NMR spectra of **1-BTrip** in CD_2Cl_2. * = CH_2Cl_2, ** = H_2O.

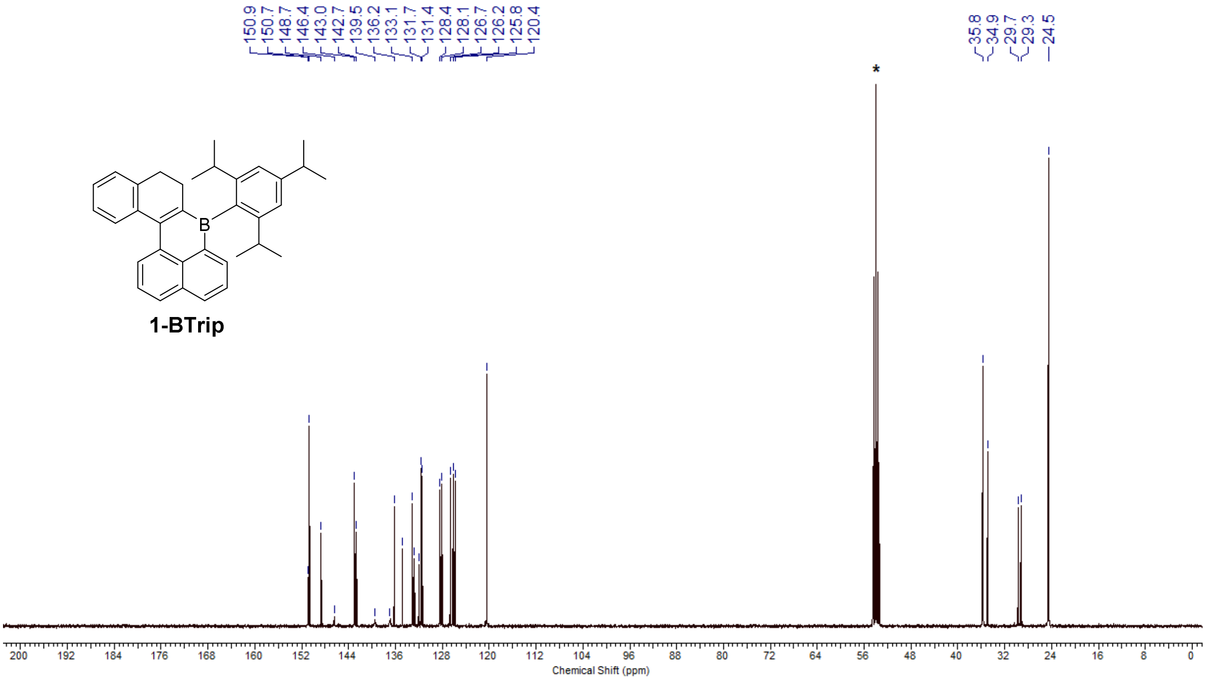

$^{13}C\{^{1}H\}$ NMR spectra of **1-BTrip** in CD_2Cl_2. * = CD_2Cl_2.

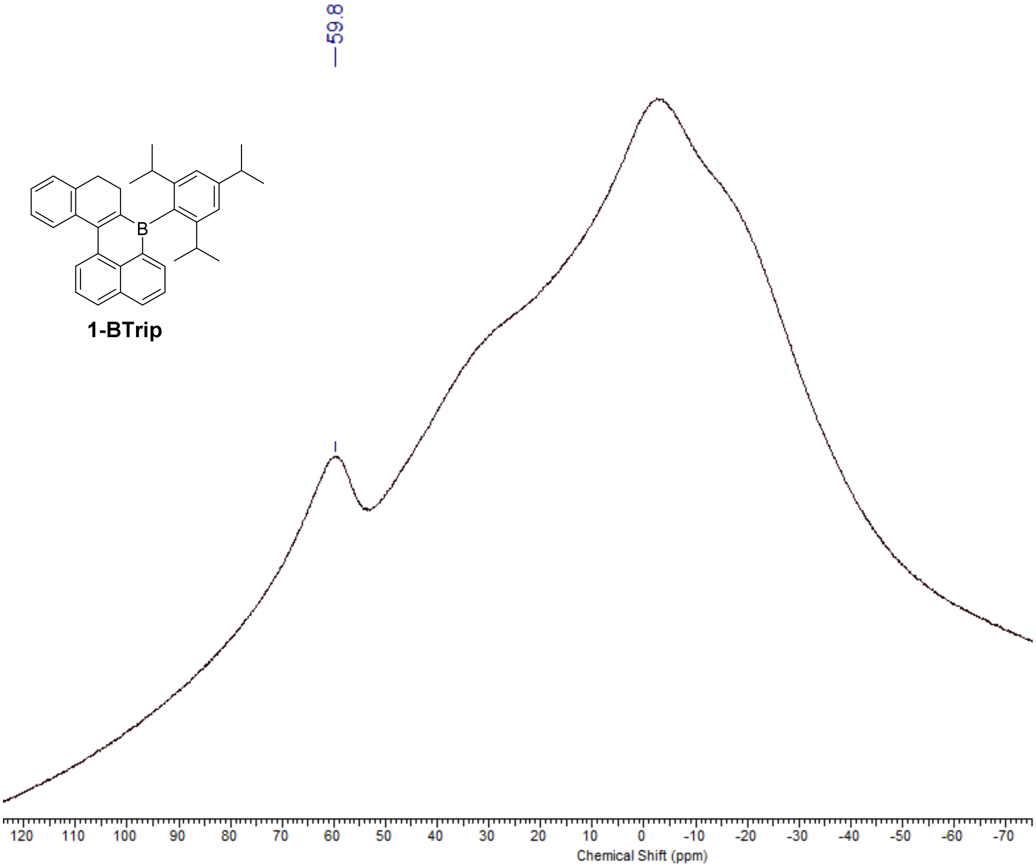

^{11}B NMR spectra of **1-BTrip** in CD_2Cl_2.

2-BTrip

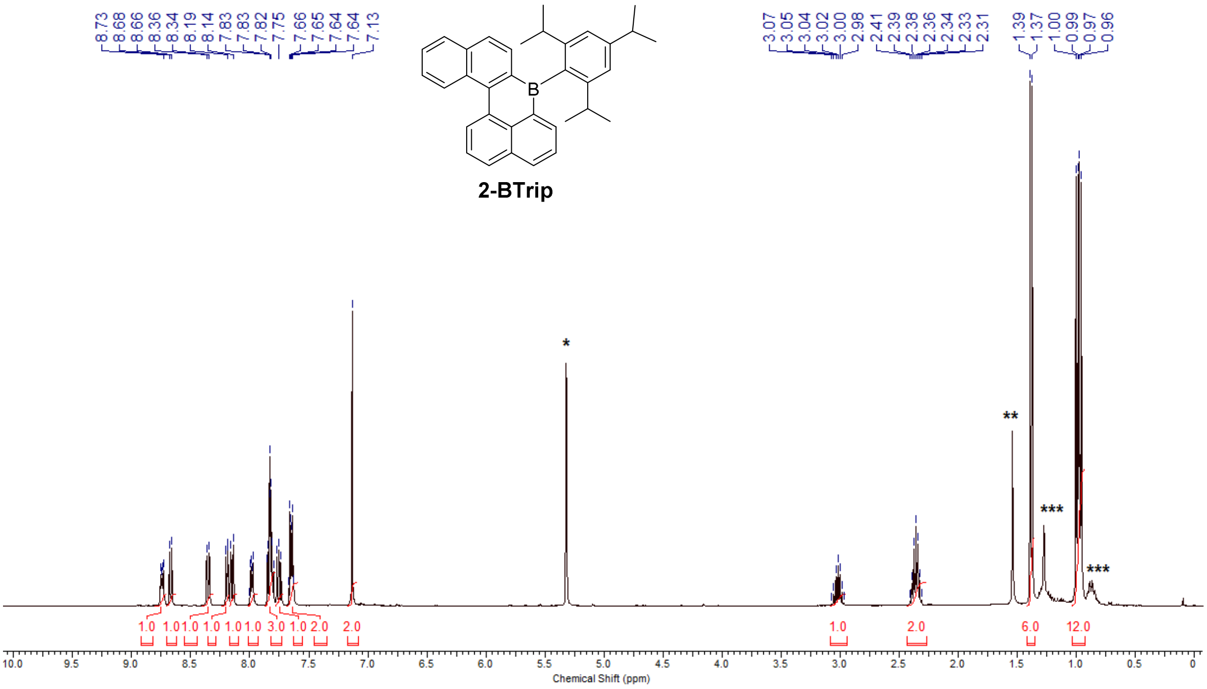

^{1}H NMR spectra of **1-BTrip** in CD_2Cl_2. * = CH_2Cl_2, ** = H_2O, *** = Apiezon H grease.

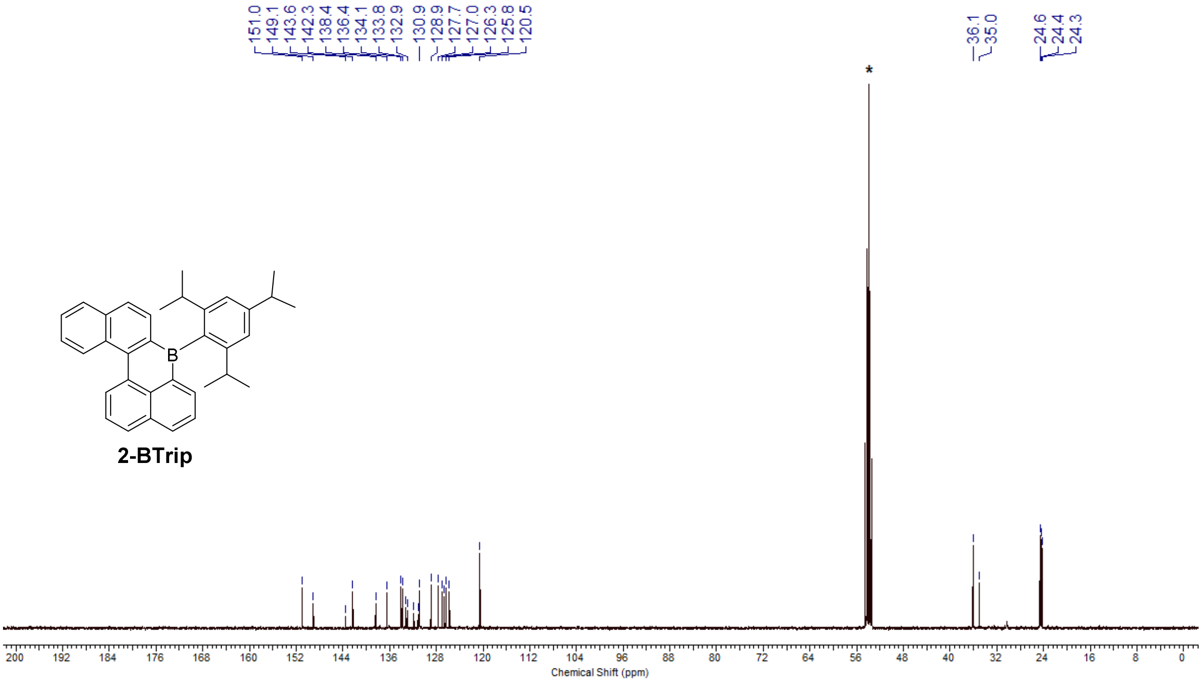

^{13}C{^{1}H} NMR spectra of **2-BTrip** in CD_2Cl_2. * = CD_2Cl_2.

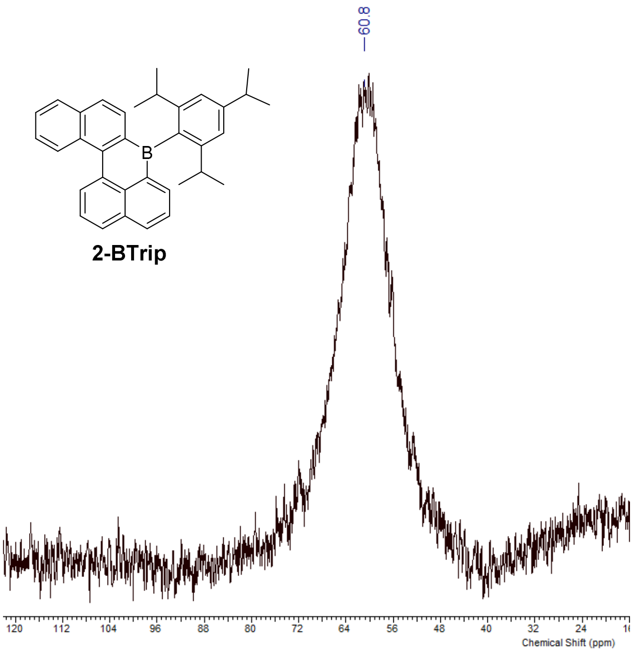

^{11}B ^{11}B NMR spectra of **2-BTrip** in CD_2Cl_2.

3

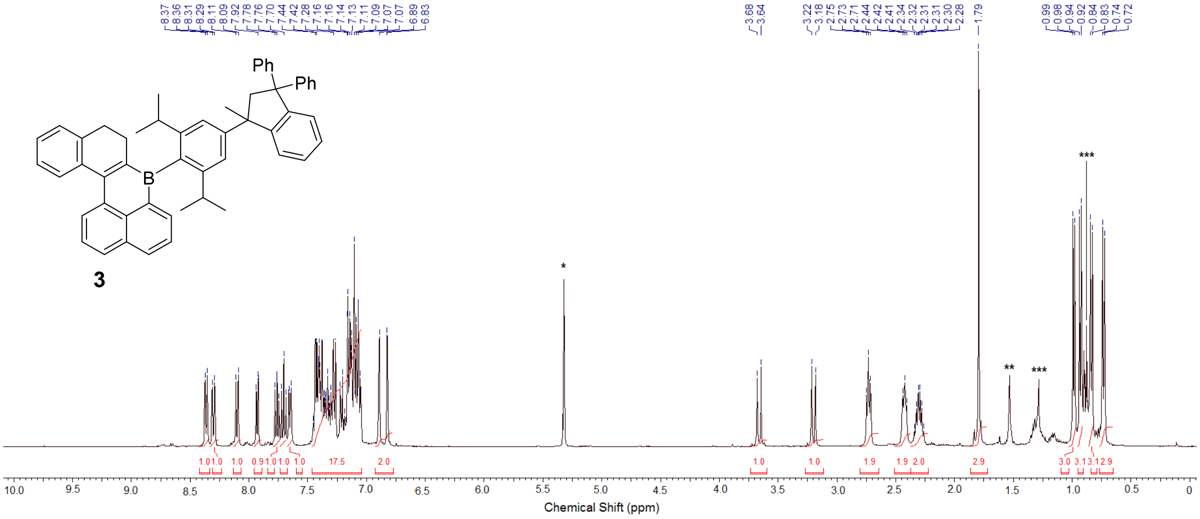

^{1}H NMR spectra of **3** in CD_2Cl_2. * = CH_2Cl_2, ** = H_2O, *** = petroleum ether + Apiezon H grease.

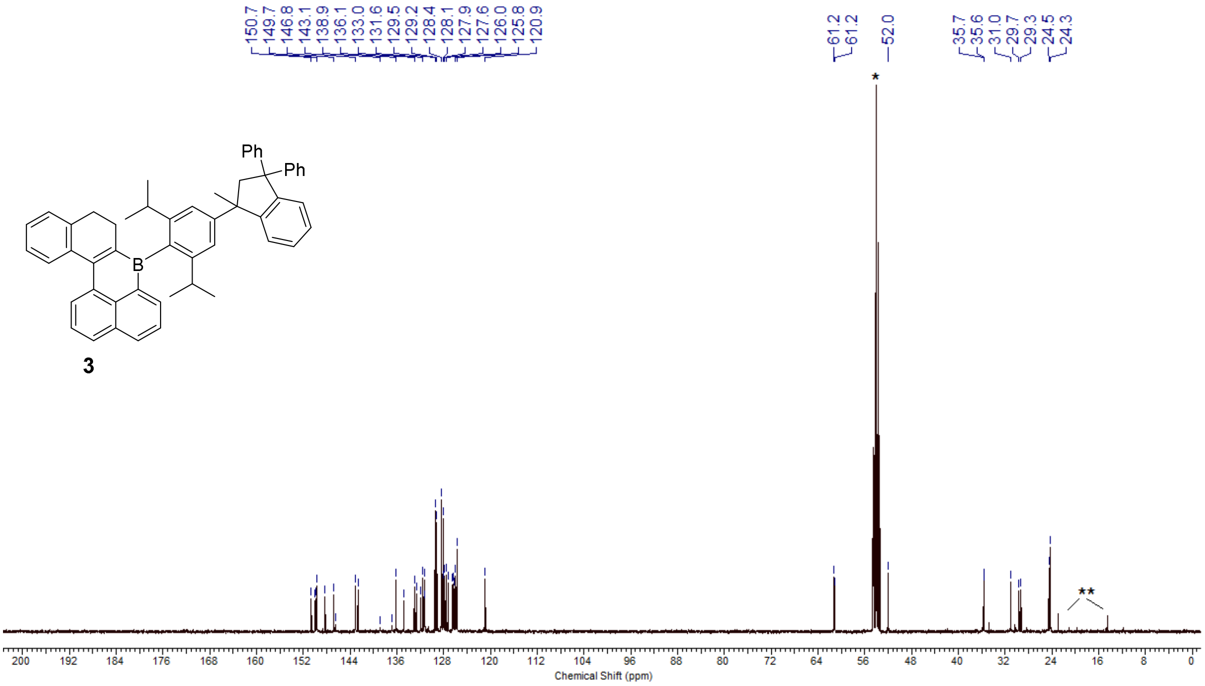

3

$^{13}C\{^{1}H\}$ NMR spectra of **3** in CD_2Cl_2. * = CD_2Cl_2, ** = Hexane

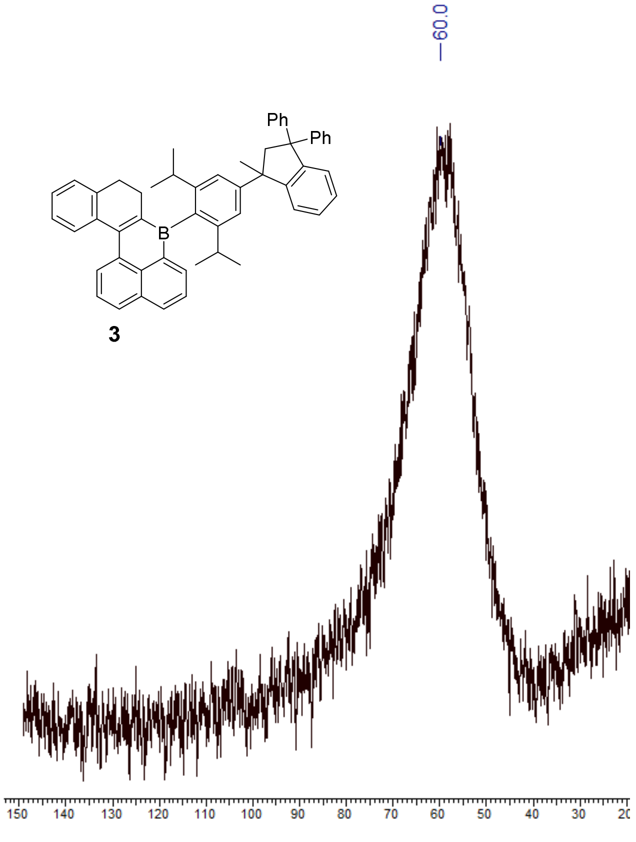

3

^{11}B NMR spectra of **3** in CD_2Cl_2.

5

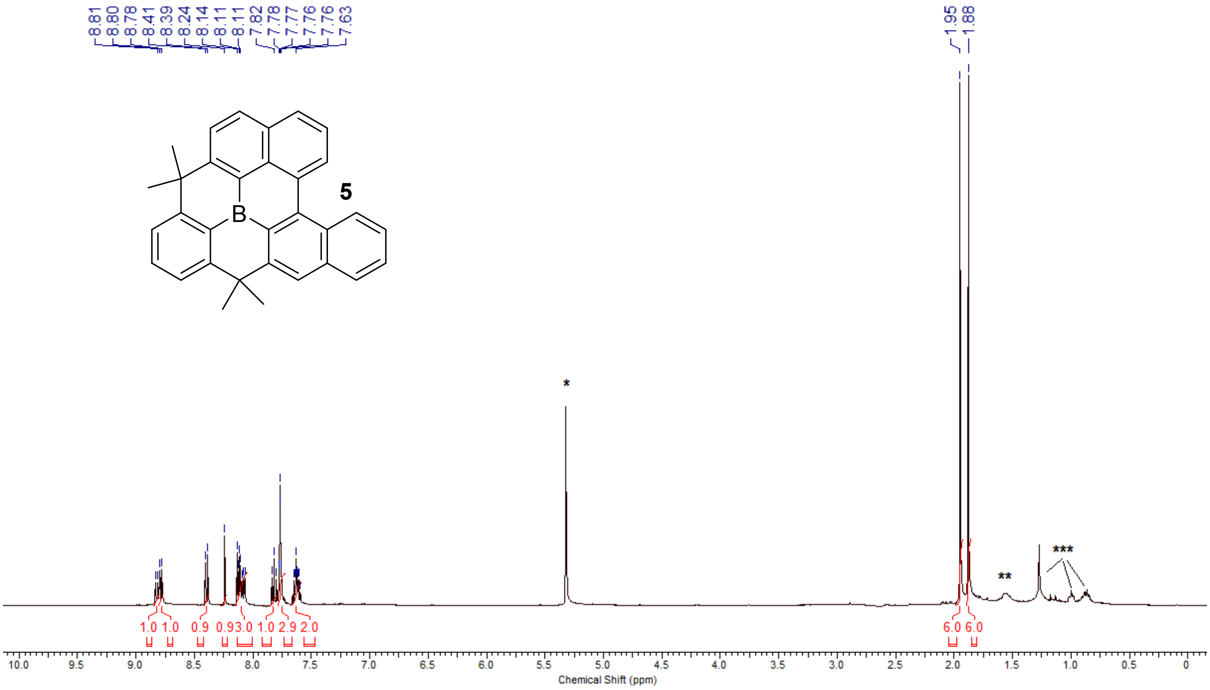

^{1}H NMR spectra of **5** in CD_2Cl_2. * = CH_2Cl_2, ** = H_2O, *** = Apiezon H grease+ Petroleum ether.

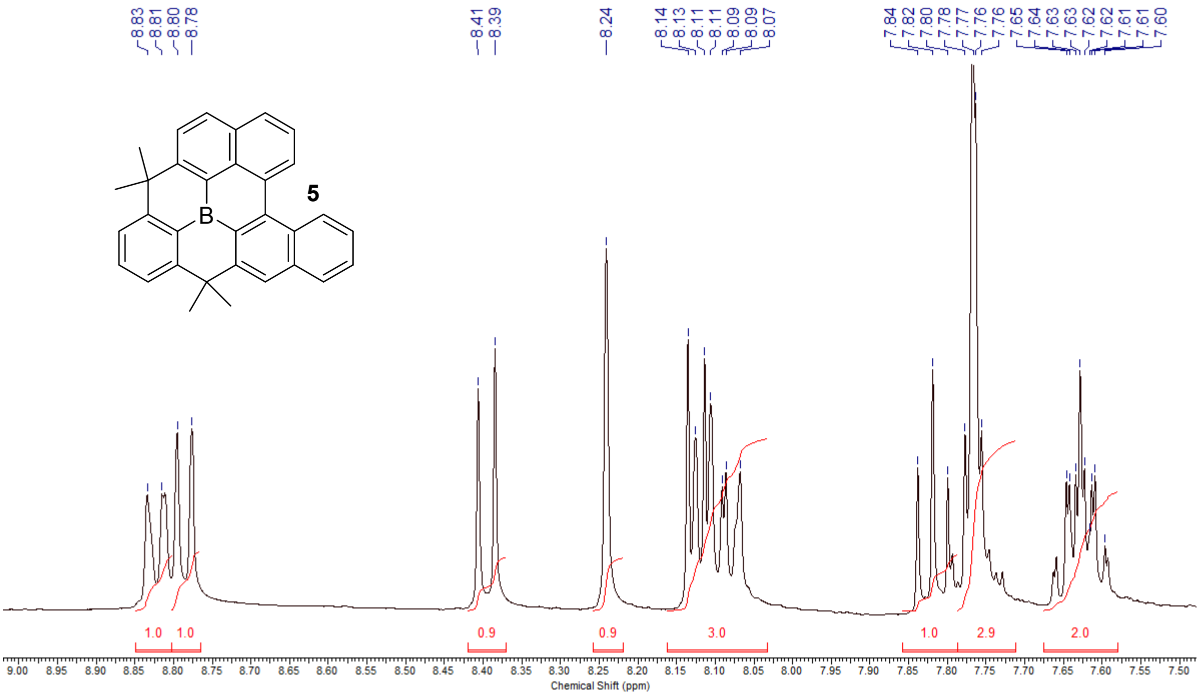

^{1}H NMR spectra of **5** in CD_2Cl_2 (9.0-7.5 ppm).

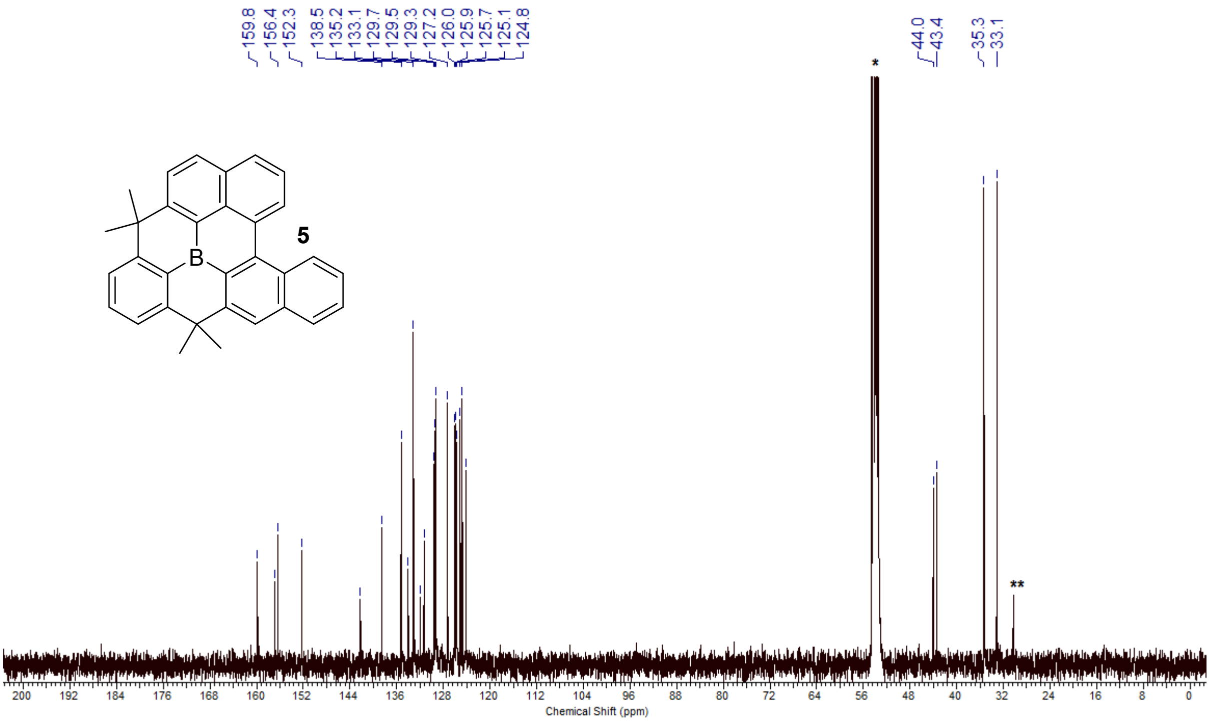

$^{13}C\{^{1}H\}$ NMR spectra of **5** in $CDCl_3$. * = $CDCl_3$, ** = Apiezon H grease.

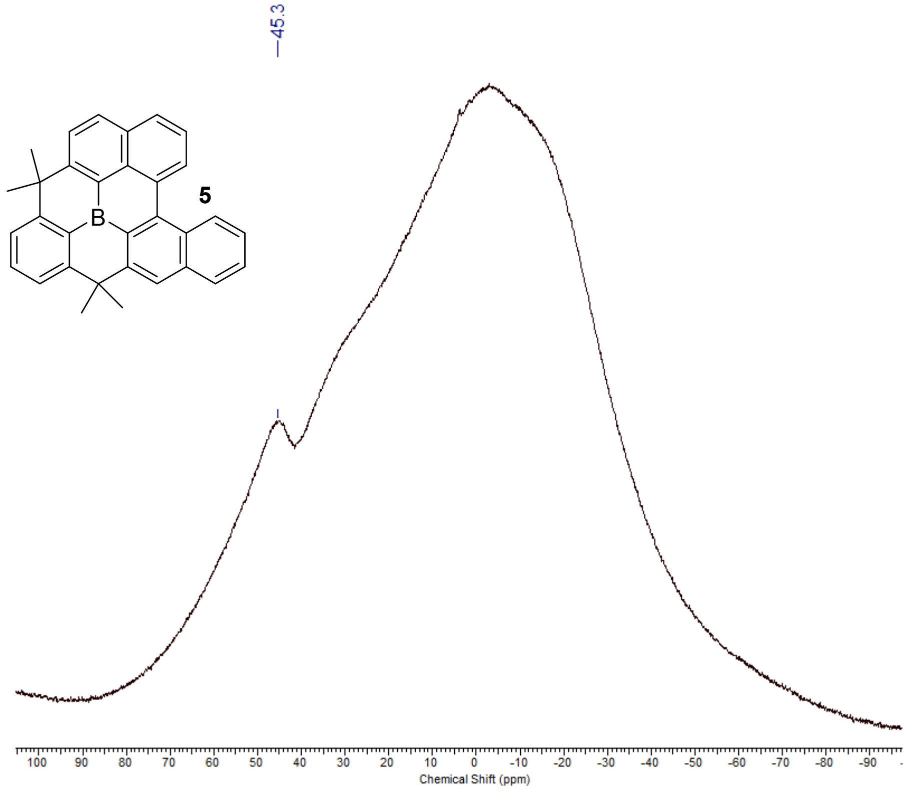

^{11}B NMR spectra of **5** in CD_2Cl_2.

6-BMes

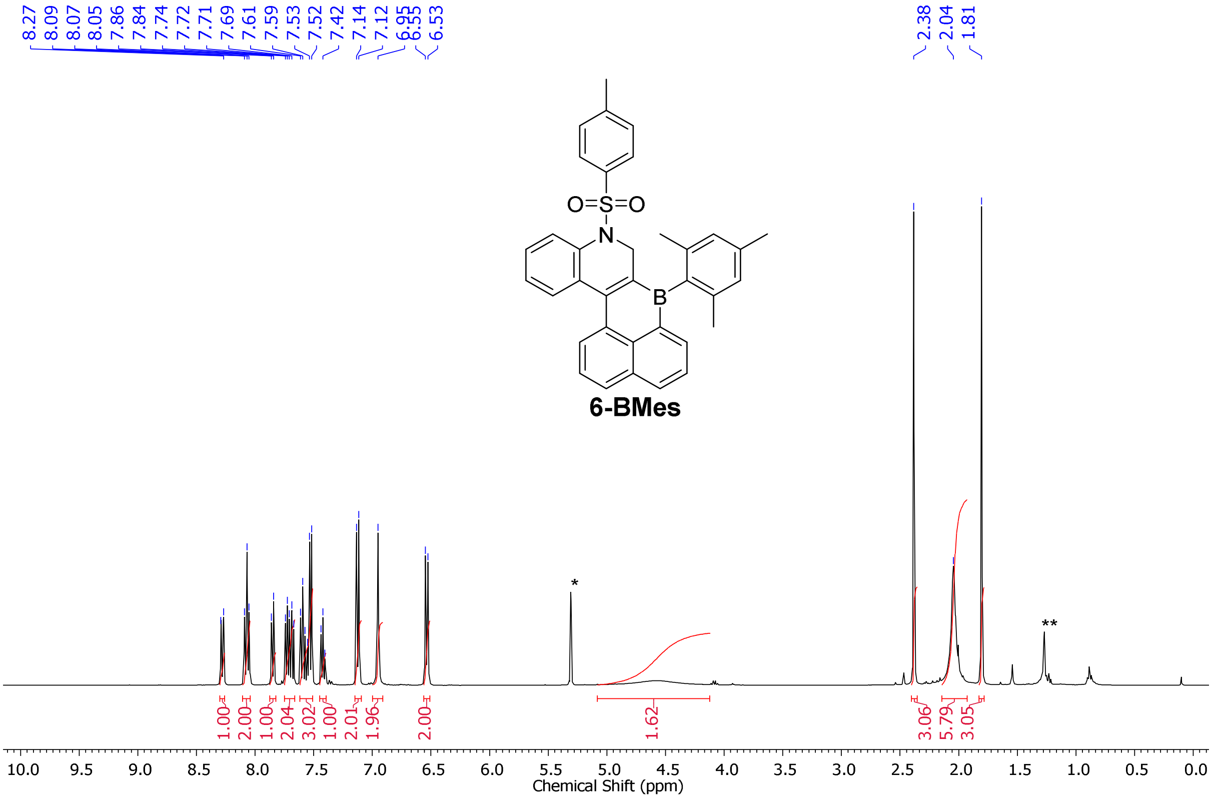

^{1}H NMR spectra of **6-BMes** in CD_2Cl_2. * = CD_2Cl_2, ** = apiezon grease.

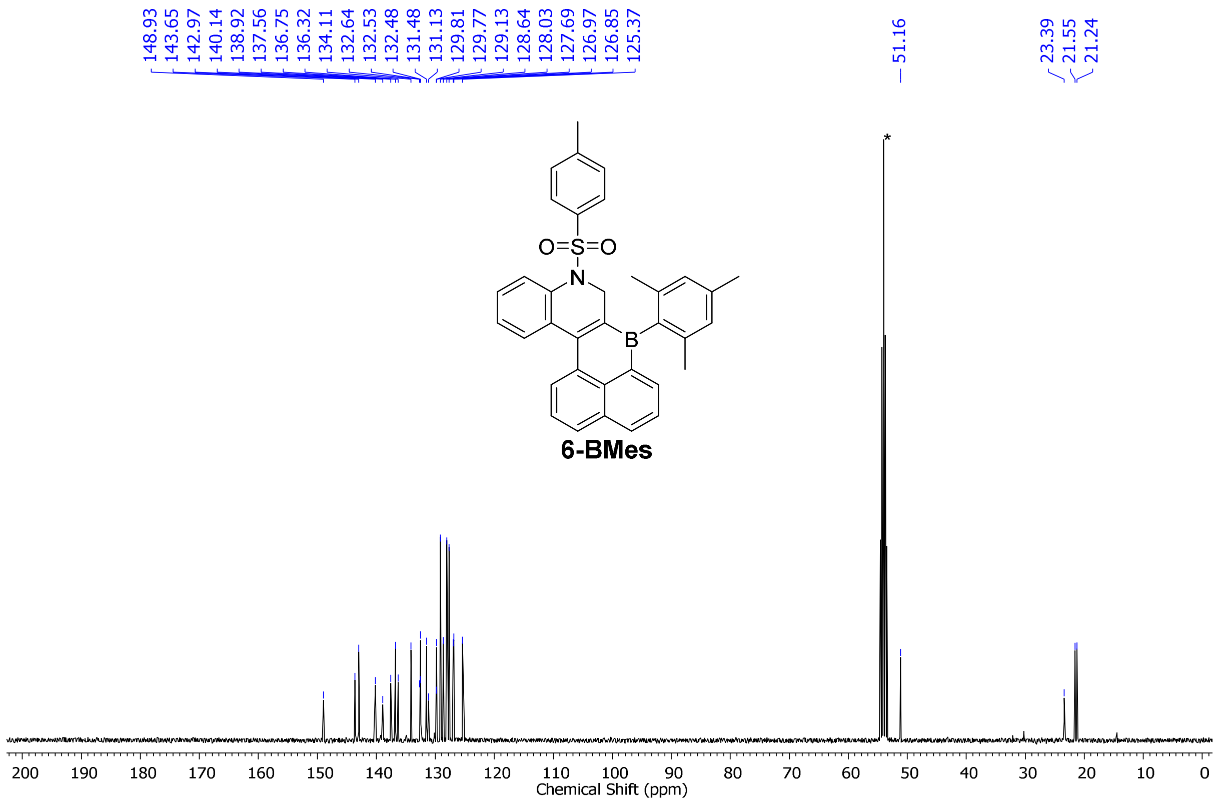

$^{13}C\{^1H\}$ NMR spectra of **6-BMes** in CD_2Cl_2. * = CD_2Cl_2.

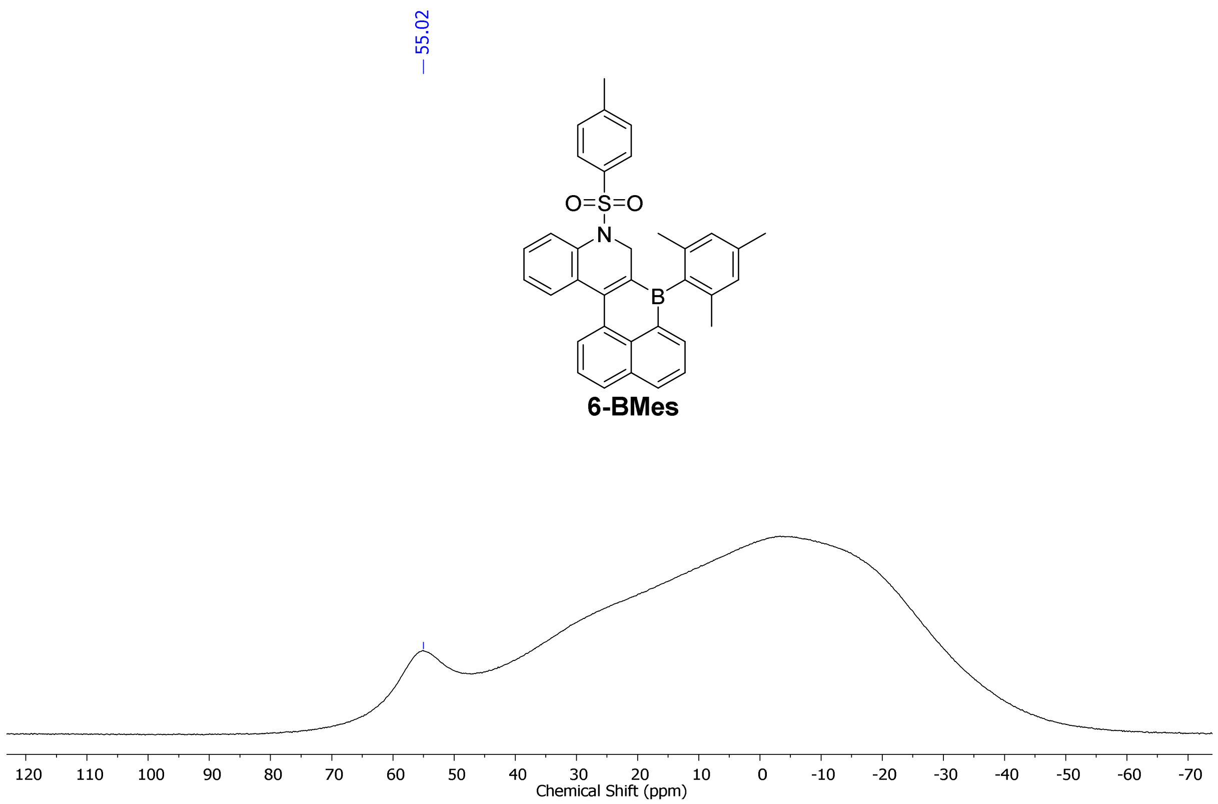

^{11}B NMR spectra of **6-BMes** in CD_2Cl_2.

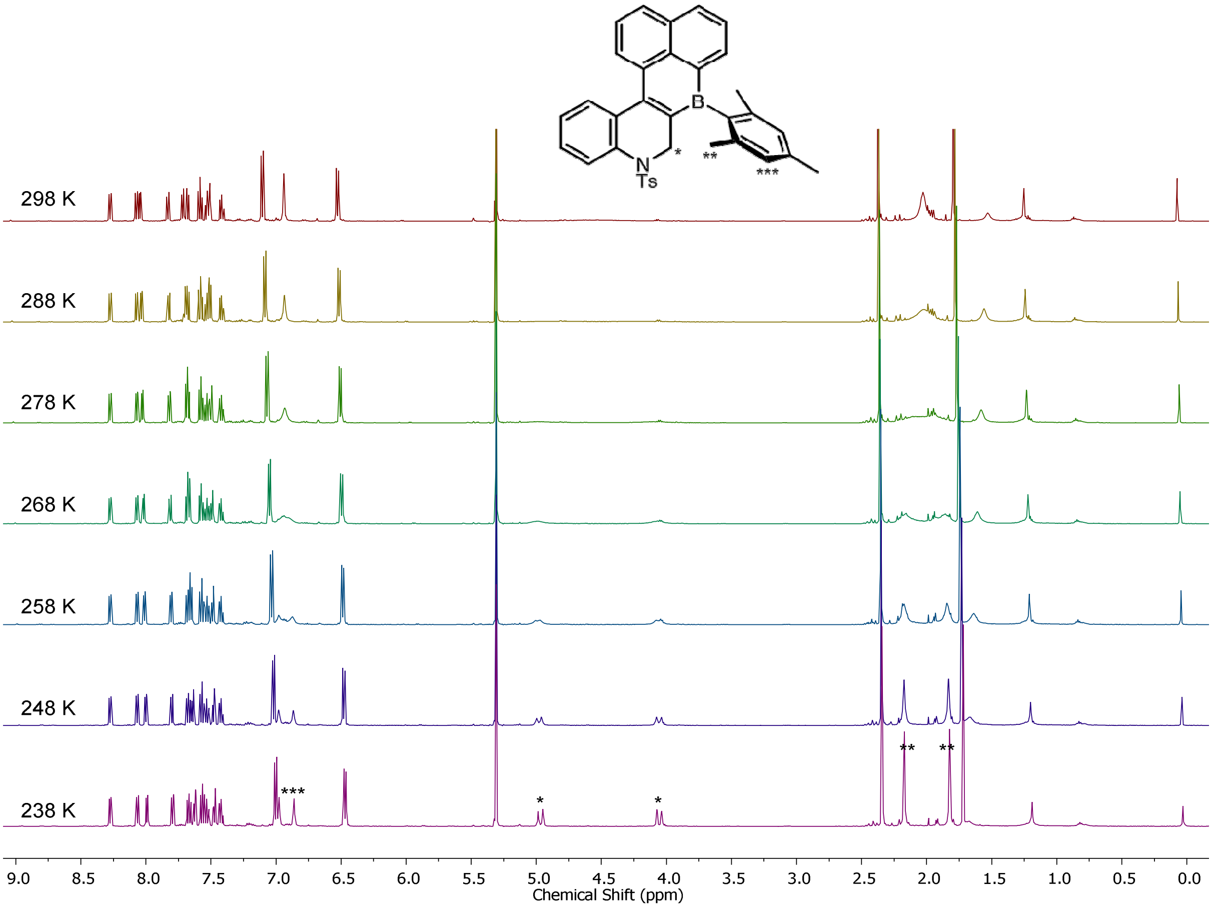

Variable temperature ^{1}H NMR spectra of **6-BMes** in CD_2Cl_2. * = hydroquinoline-CH_2, J = 18.1 Hz at 238 K; ** = mesityl-oCH_3; *** = mesityl-mCH.

7-BMes

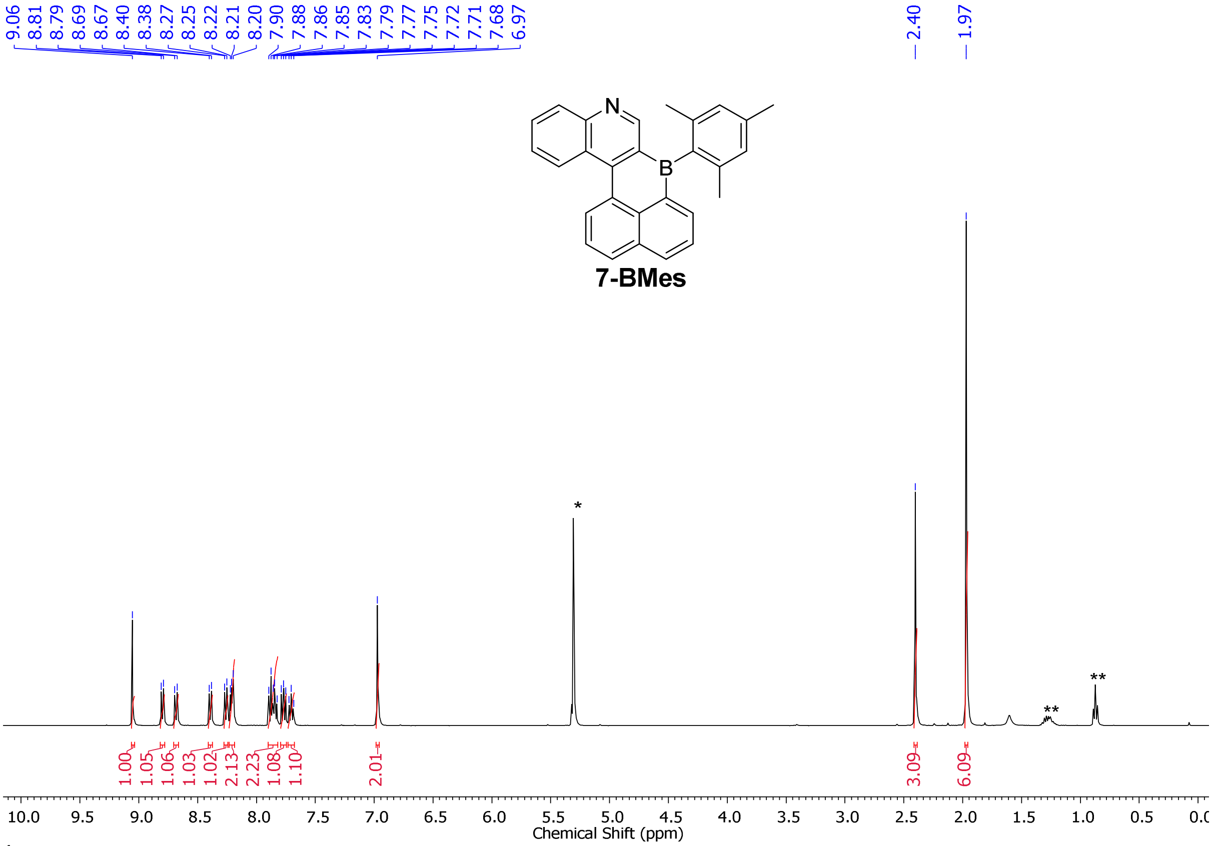

^{1}H NMR spectra of **7-BMes** in CD_2Cl_2. * = CD_2Cl_2, ** = hexanes.

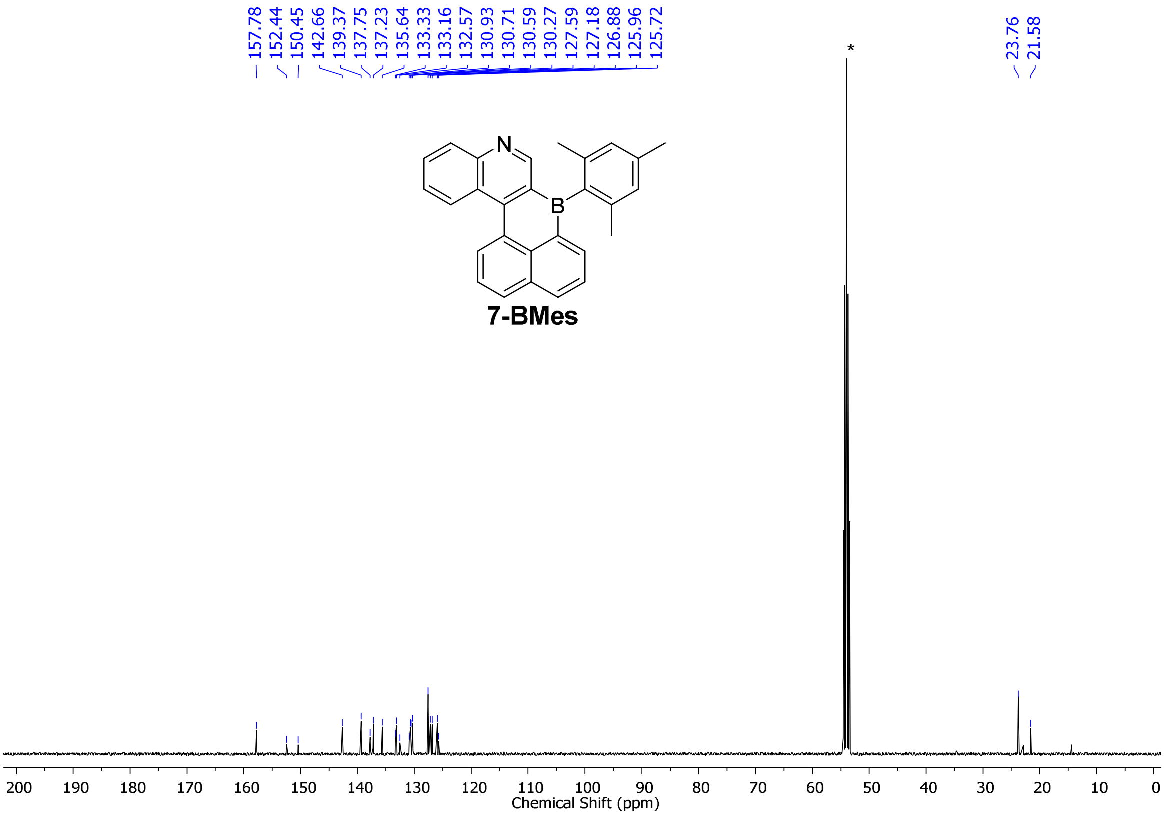

$^{13}C\{^{1}H\}$ NMR spectra of **7-BMes** in CD_2Cl_2. * = CD_2Cl_2.

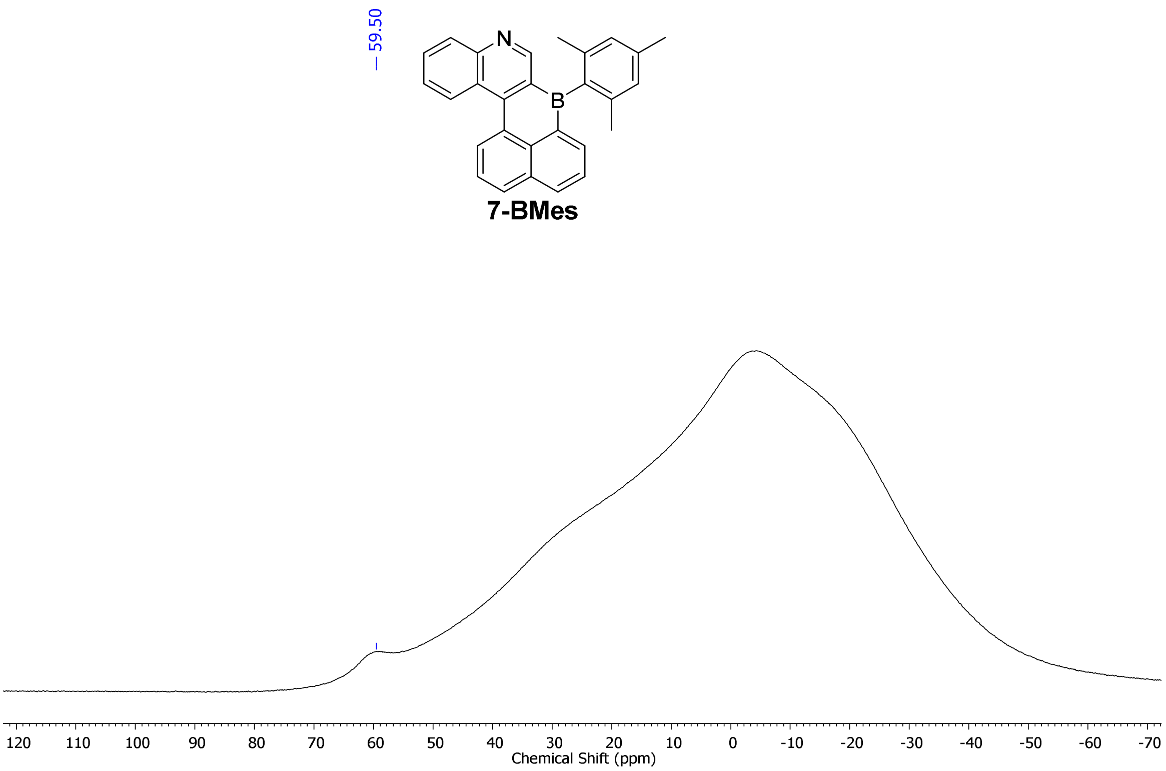

^{11}B NMR spectra of **7-BMes** in CD_2Cl_2.

<u>8</u>

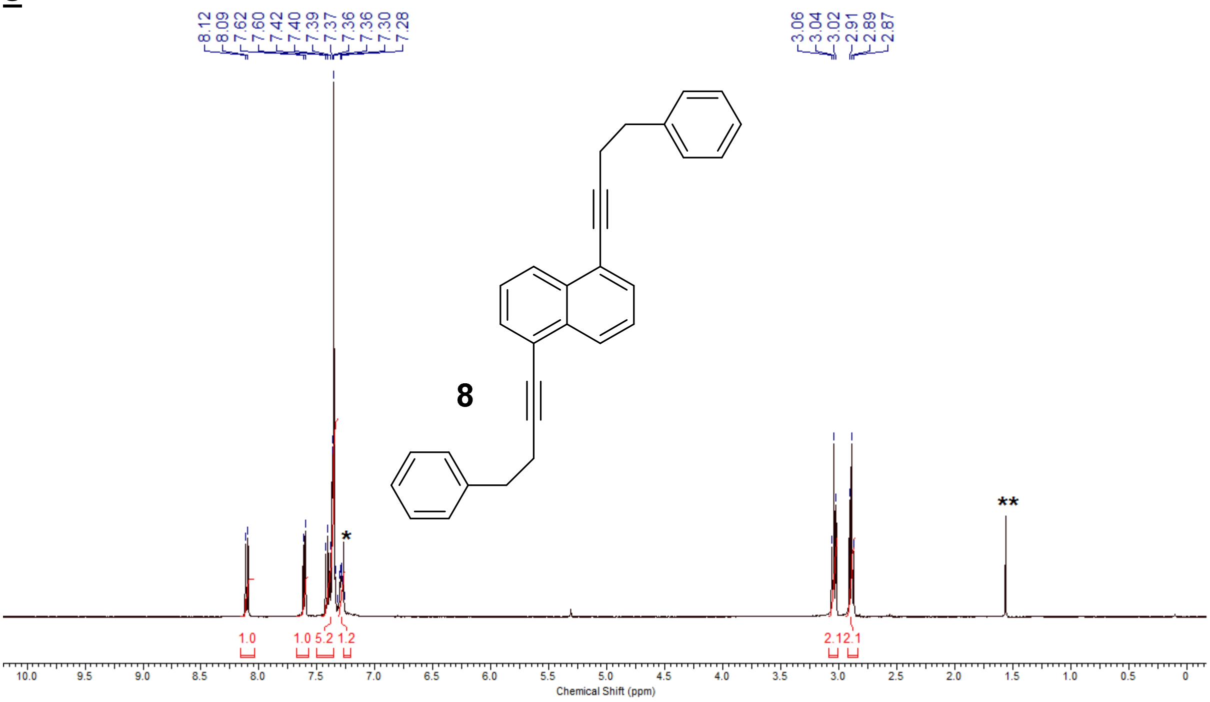

^{1}H NMR spectra of **8** in $CDCl_3$. * = $CHCl_3$, ** = H_2O.

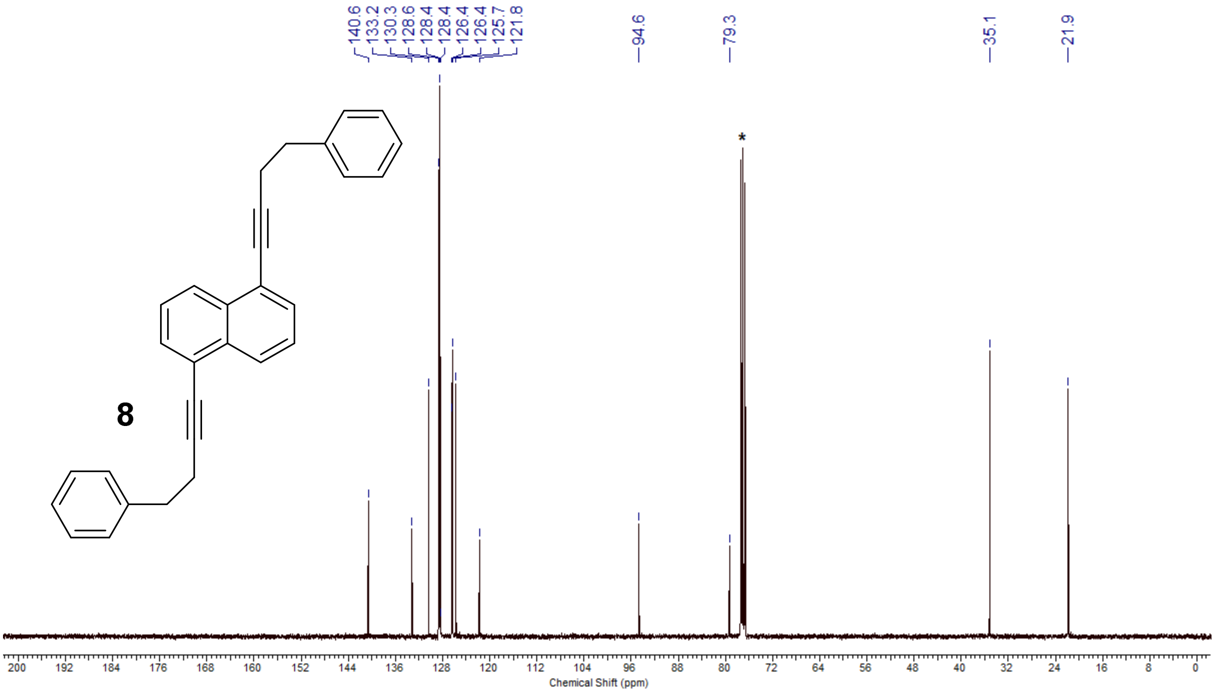

$^{13}C\{^{1}H\}$ NMR spectra of **8** in $CDCl_3$. * = $CDCl_3$.

9-BCl

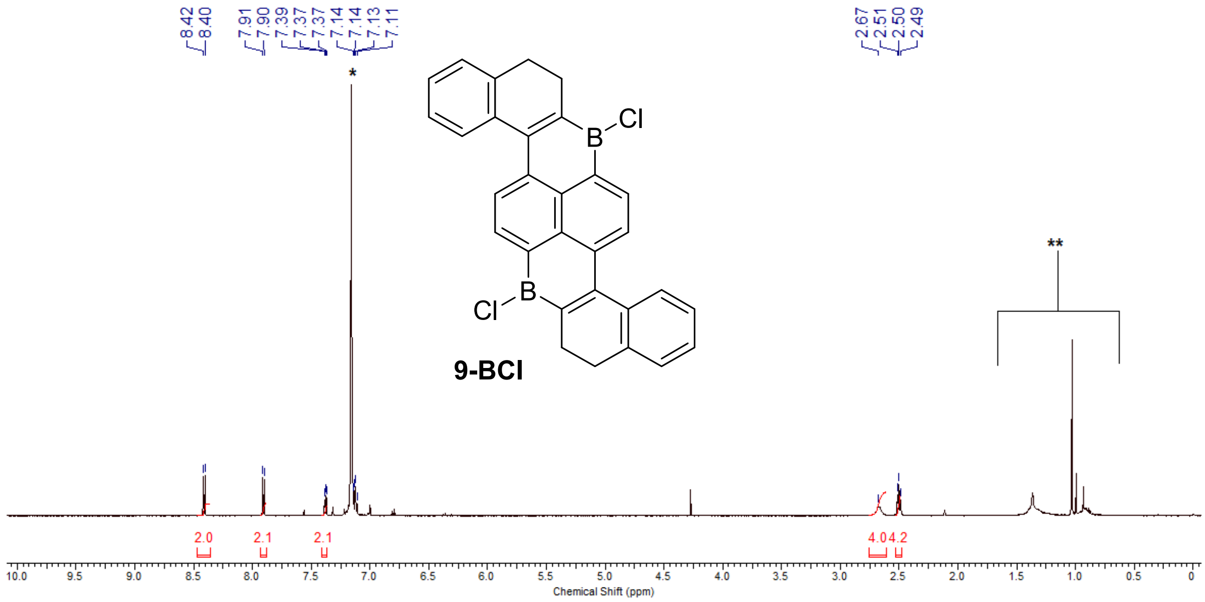

^{1}H NMR spectra of **9-BCl** in C_6D_6. * = C_6D_5H, ** = unidentified impurities.

9-BMes

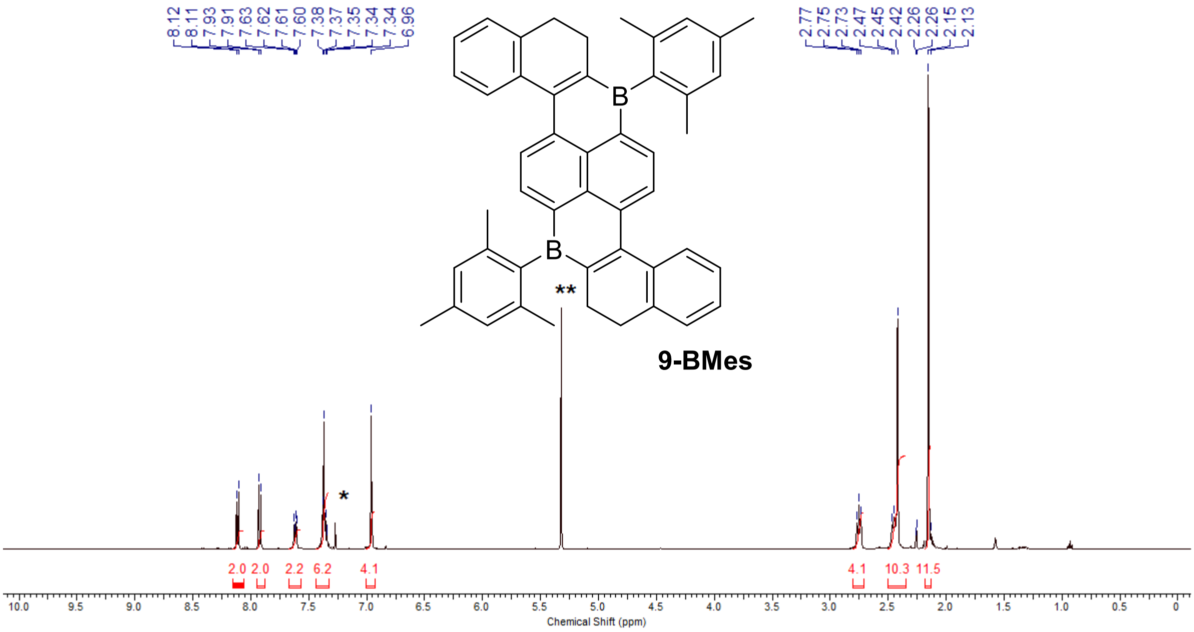

^{1}H NMR spectra of **9-BMes** in $CDCl_3$. * = $CHCl_3$, ** = CH_2Cl_2.

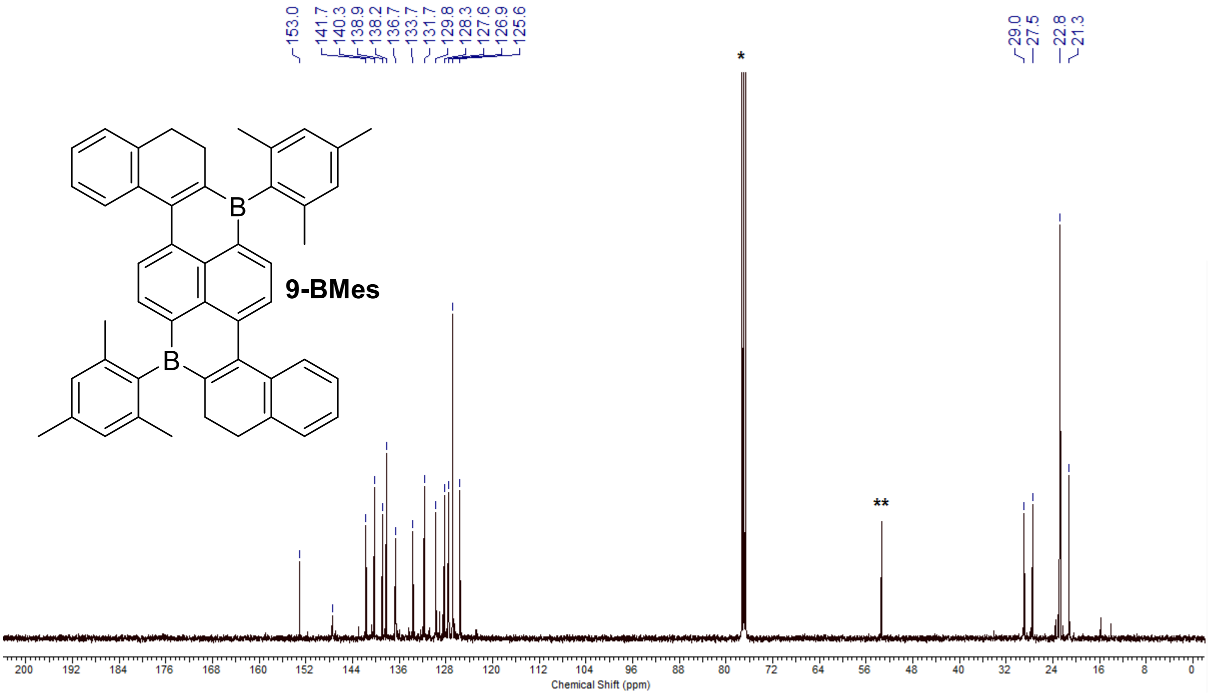

^{13}C{^{1}H} NMR spectra of **9-BMes** in $CDCl_3$. * = $CDCl_3$, ** = CH_2Cl_2.

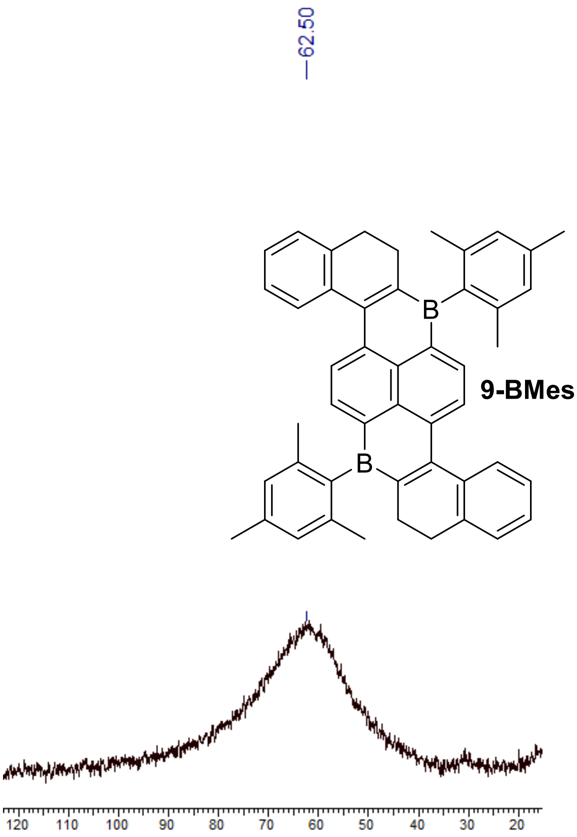

^{11}B NMR spectra of **9-BMes** in $CDCl_3$.

9-BTrip

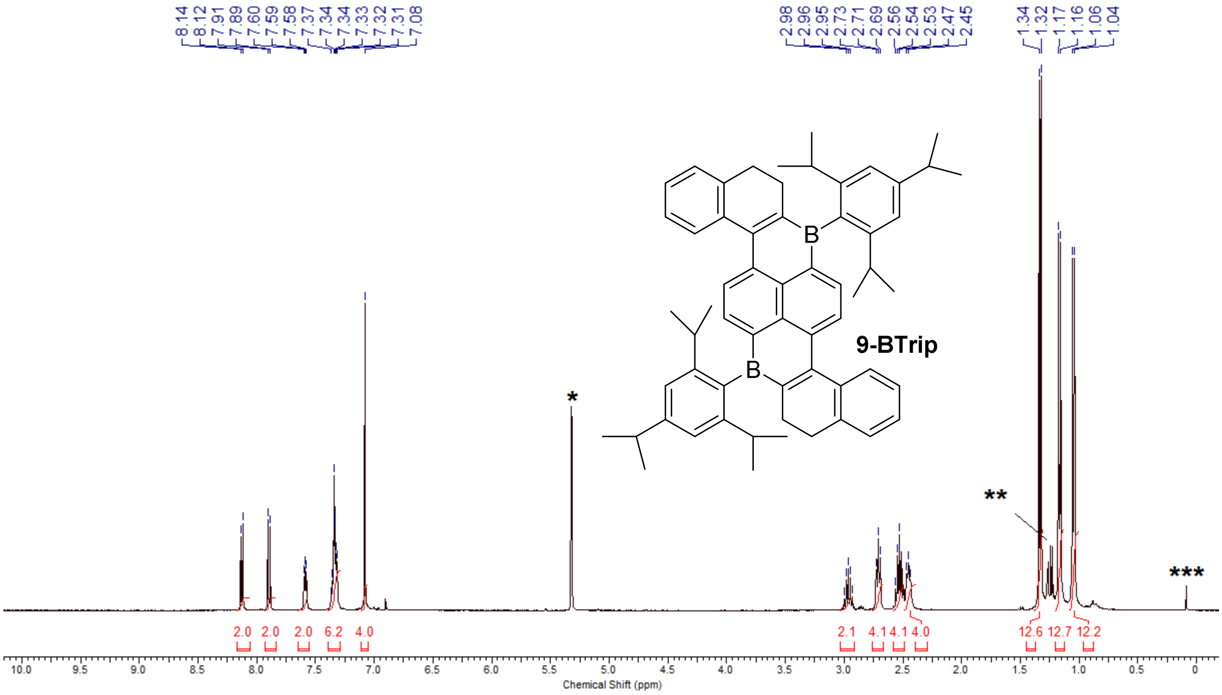

^{1}H NMR spectra of **9-BTrip** in CD_2Cl_2. * = CH_2Cl_2, ** = 1,3,5-triisopropylbenzene, *** = silicon grease.

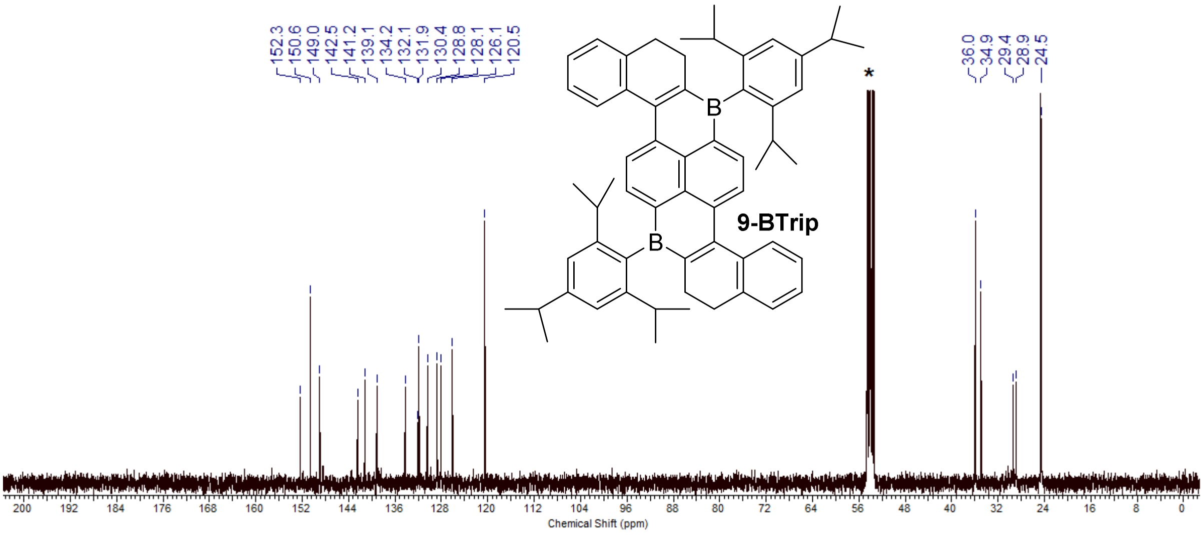

$^{13}C\{^{1}H\}$ NMR spectra of **9-BTrip** in CD_2Cl_2. * = CD_2Cl_2.

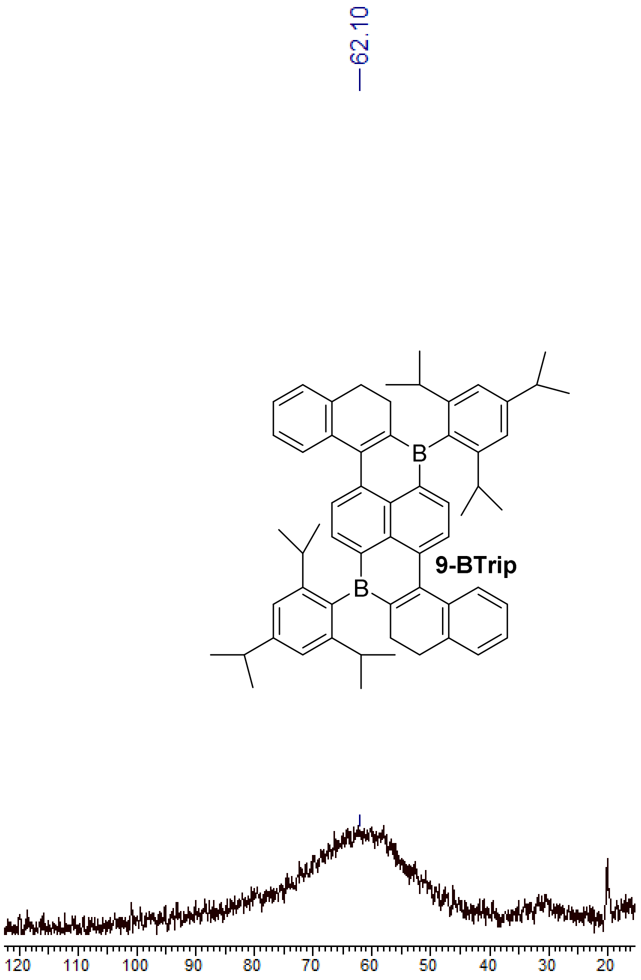

^{11}B NMR spectra of **9-BTrip** in $CDCl_3$.

10-BTrip

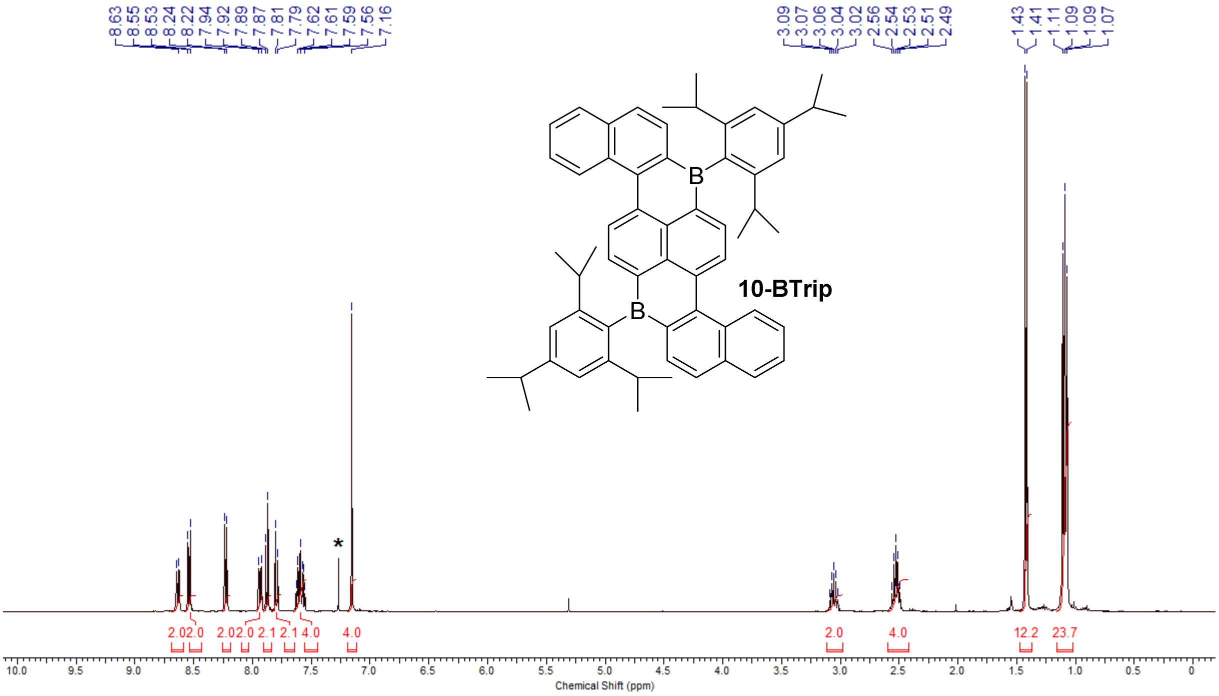

^{1}H NMR spectra of **10-BTrip** in $CDCl_3$. * = $CHCl_3$.

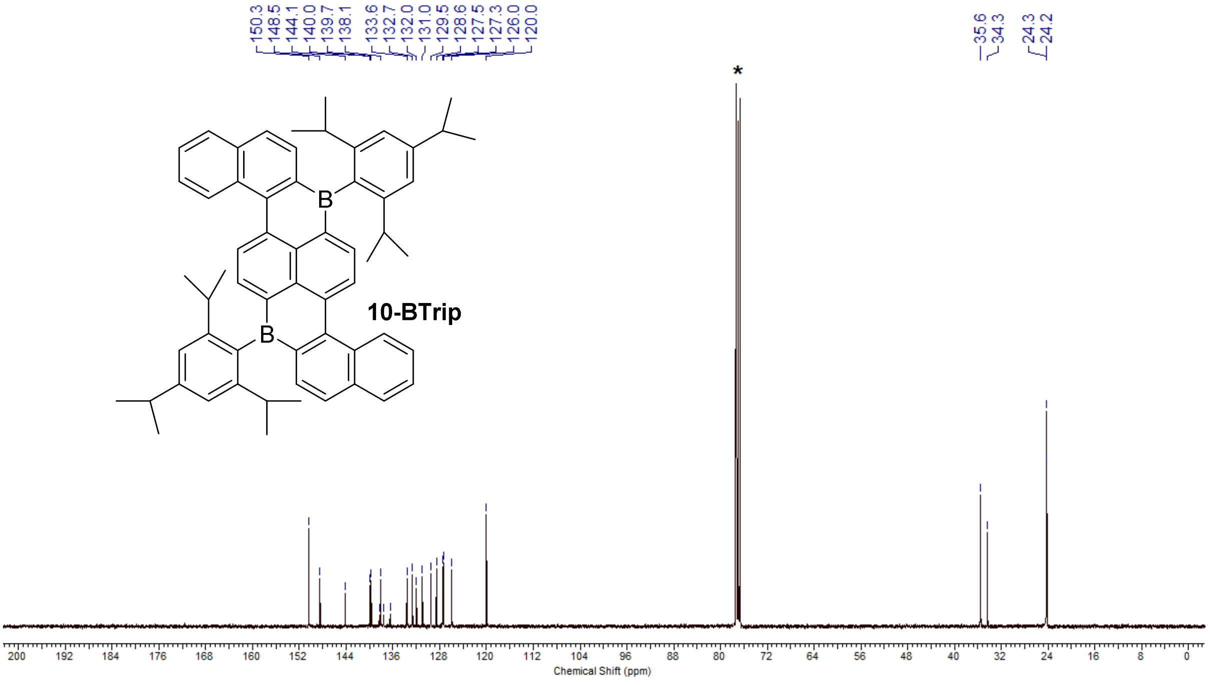

^{13}C{^{1}H} NMR spectra of **10-BTrip** in $CDCl_3$. * = $CDCl_3$.

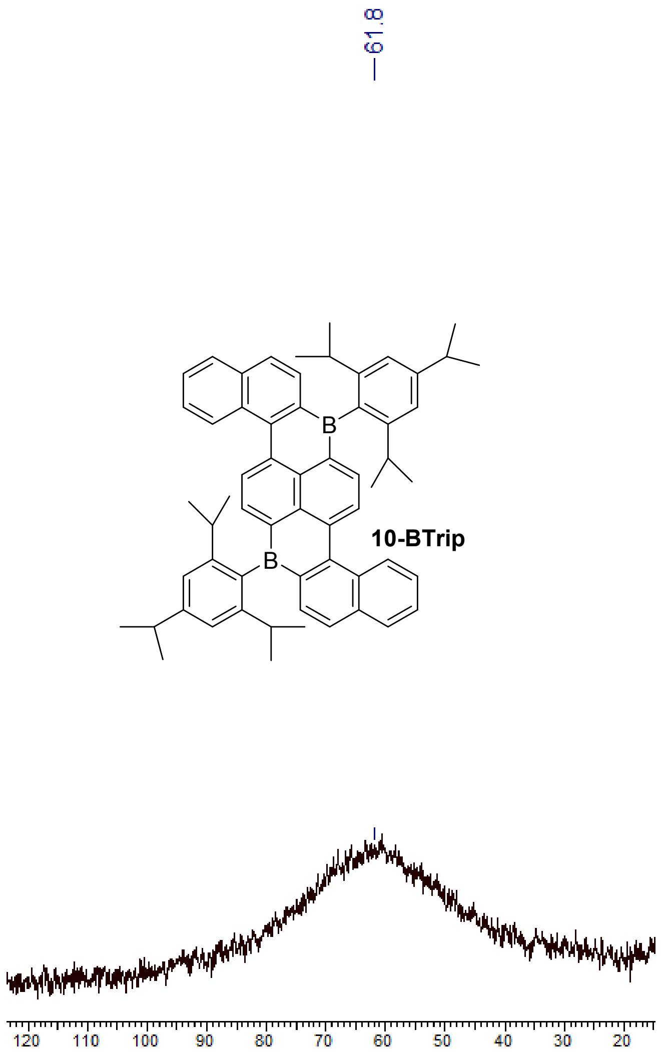

^{11}B NMR spectra of **10-BTrip** in $CDCl_3$.

11

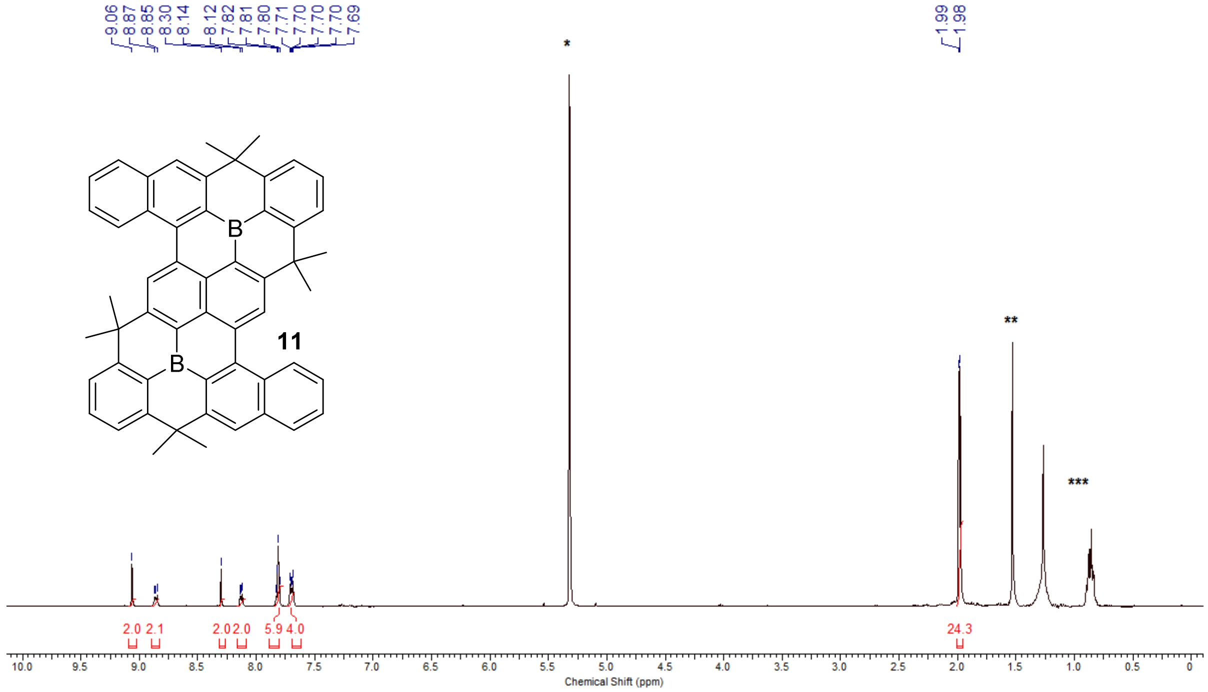

^{1}H NMR spectra of a saturated solution of **11** in CD_2Cl_2. * = CH_2Cl_2, ** = H_2O, *** = Apiezon H grease.

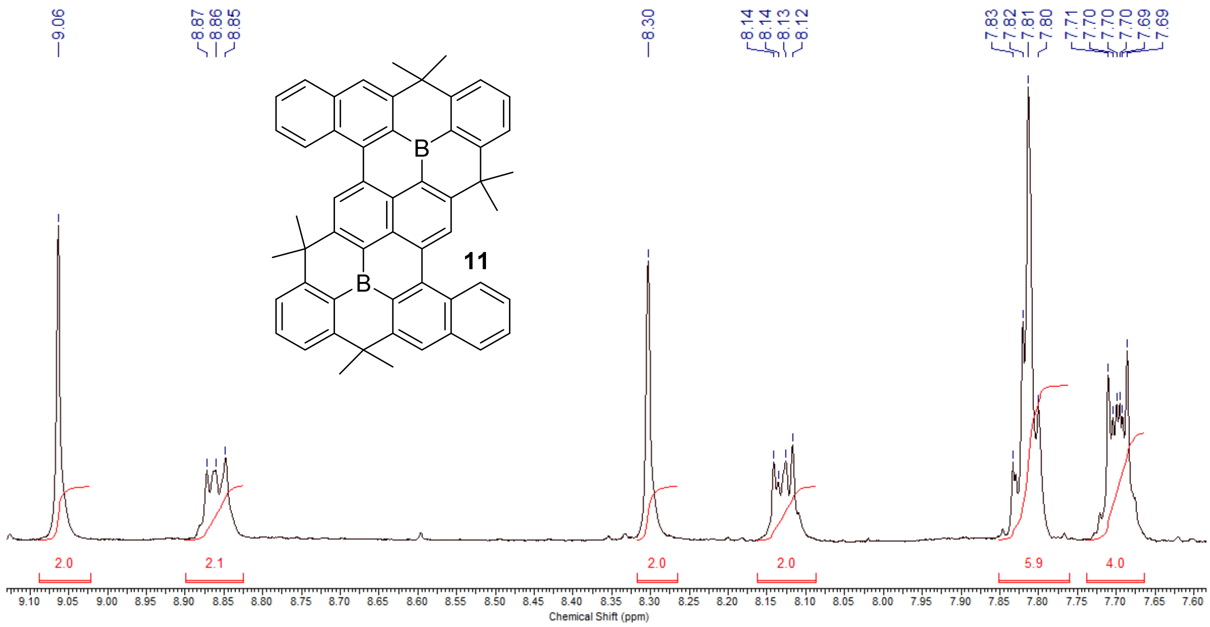

^{1}H NMR spectra of **11** in CD_2Cl_2 (9.1-7.6 ppm).

Photophysical Properties

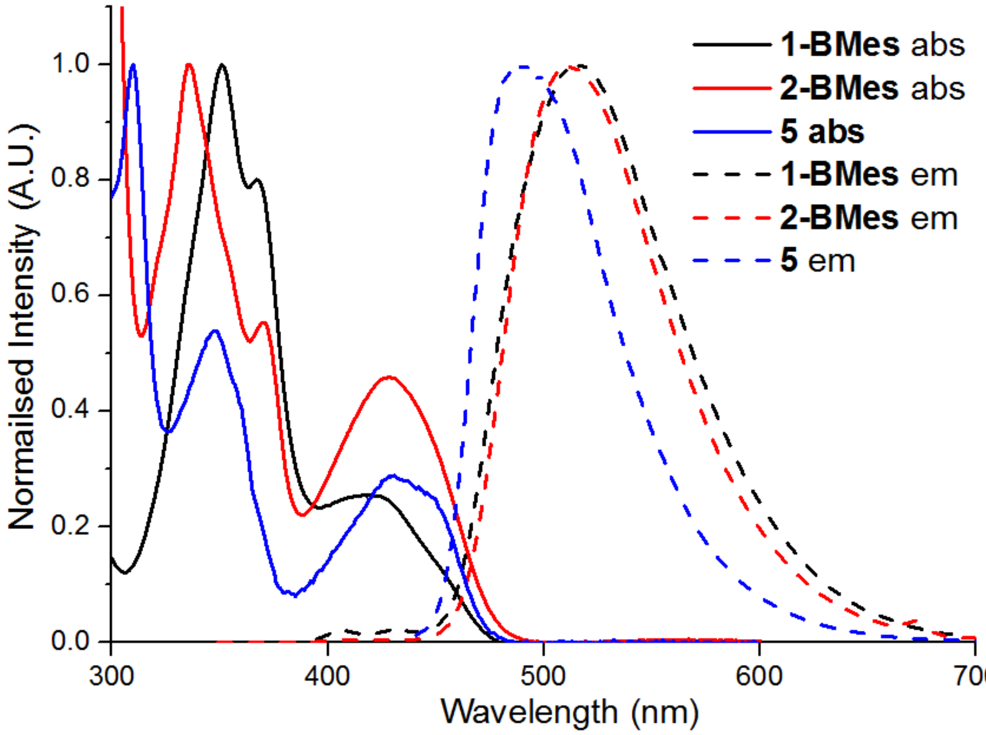

Figure S1: Normalised absorbance and emission spectra of **1-BMes** and **2-BMes** in toluene (1 x10^{-5} M). λex : **1-BMes** = 351 nm, **2-BMes** = 336 nm, **5** = 431 nm.

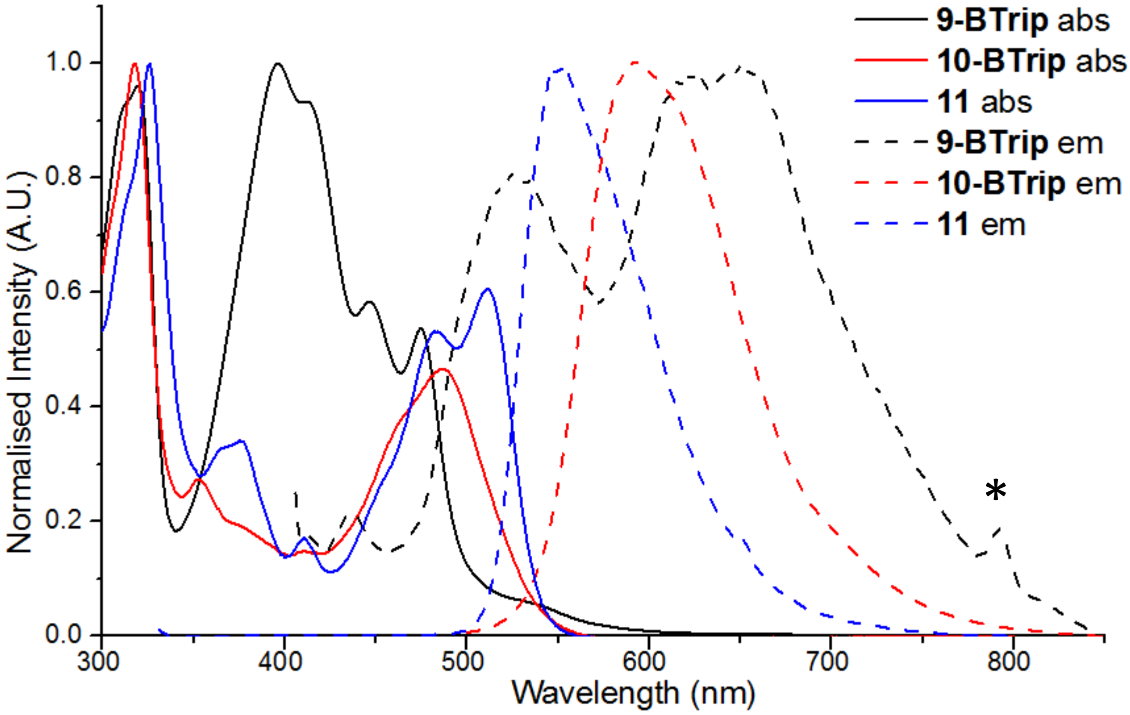

Figure S2: Normalised absorbance and emission spectra of **9-BTrip** and **10-BTrip** and **11** in toluene (1 x10^{-5} M). λex : **9-BTrip** = 396 nm, **10-BTrip** = 497 nm, **11** = 326 nm. * = half-harmonic

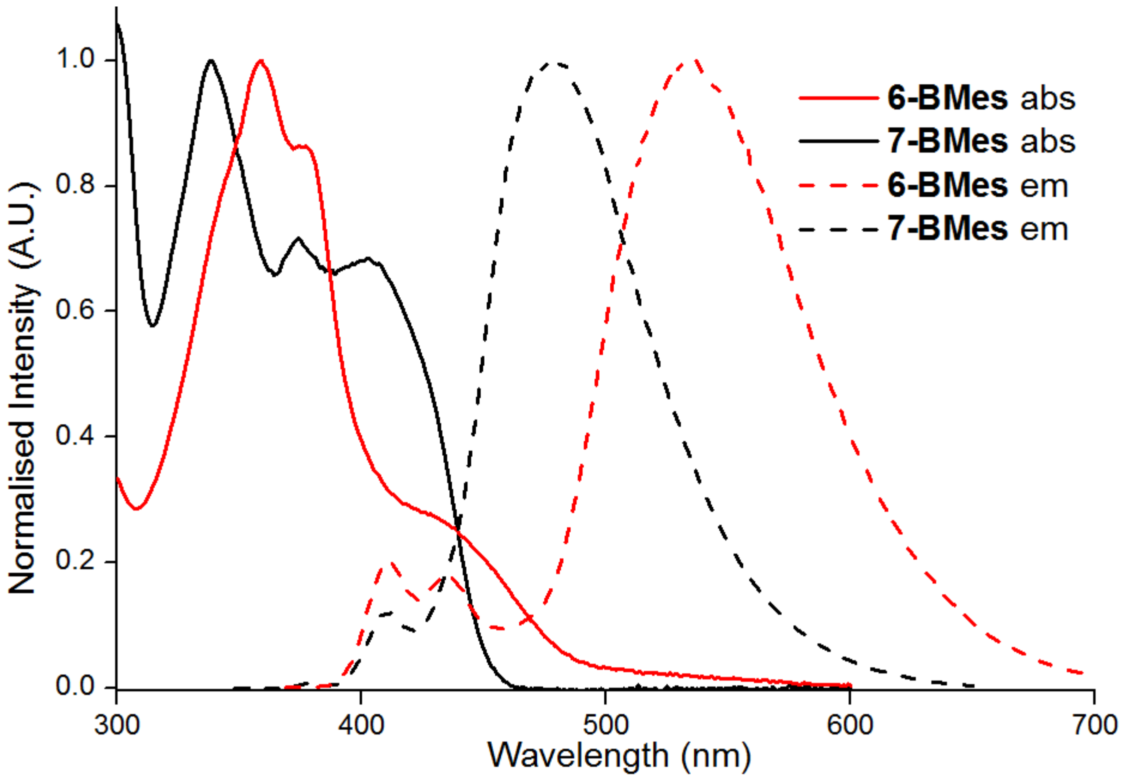

Figure S3: Normalised absorbance and emission spectra of **6-BTrip** and **7-BTrip** in toluene (1 x10^{-5} M). λex : **6-BMes** = 359 nm, **7-BMes** = 338 nm.

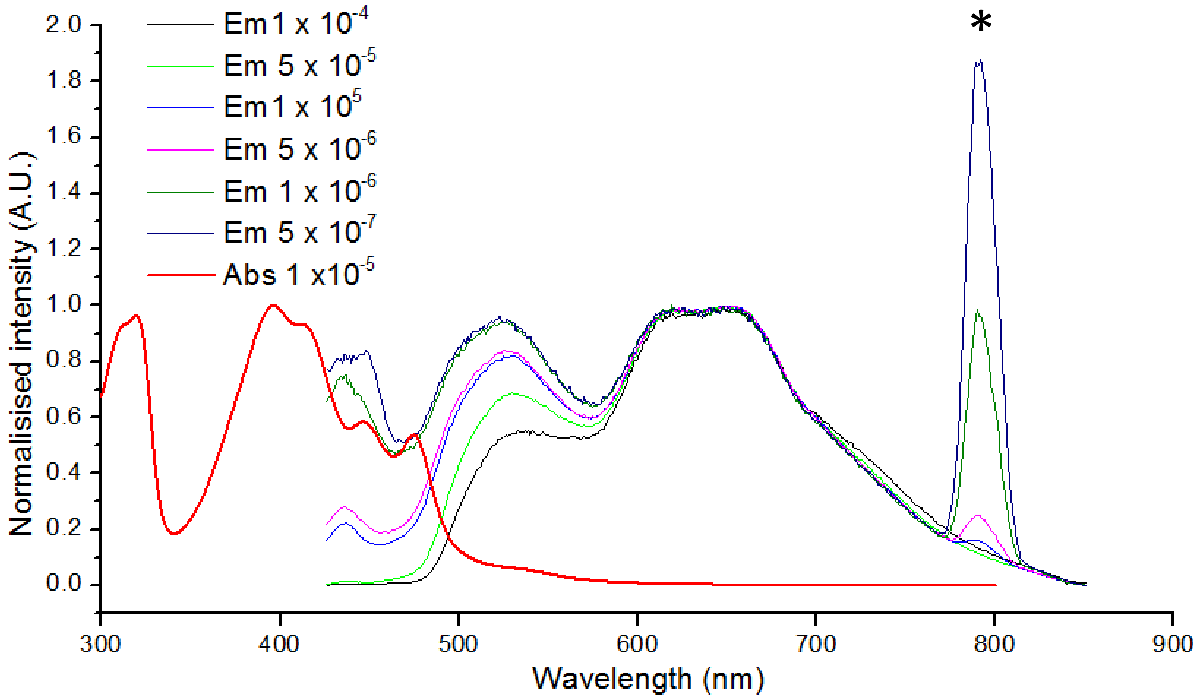

Figure S4: Normalised emission spectra of **9-BTrip** in toluene at different concentrations (in M). λex = 396 nm. * = Half-harmonic

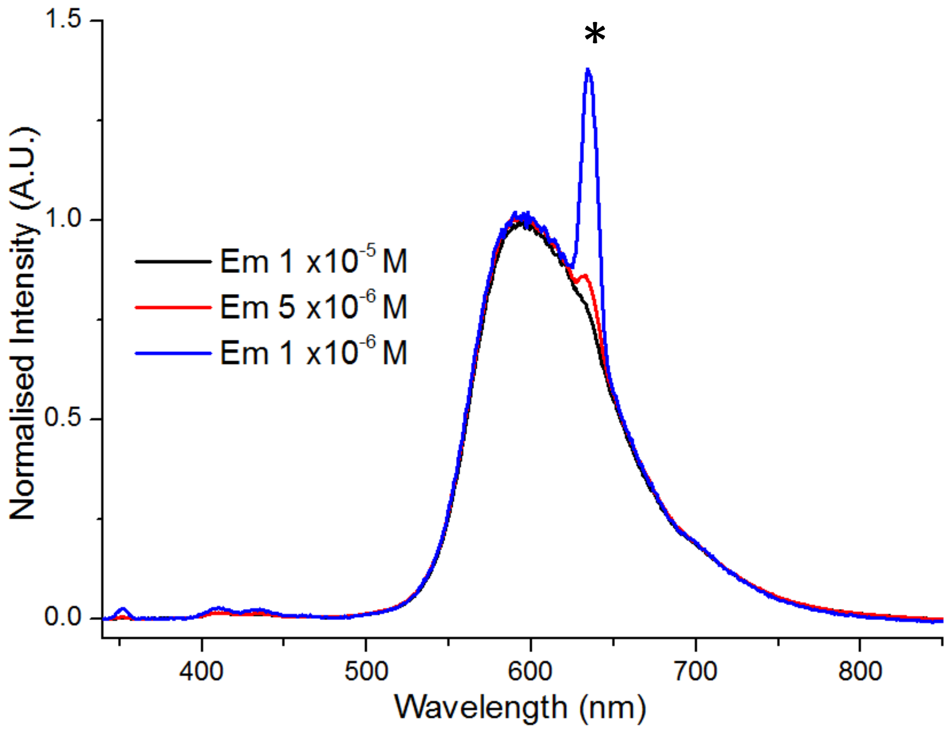

Figure S5: Normalised emission spectra of **9-BTrip** in toluene at different concentrations. λex = 318 nm. * = Half-harmonic

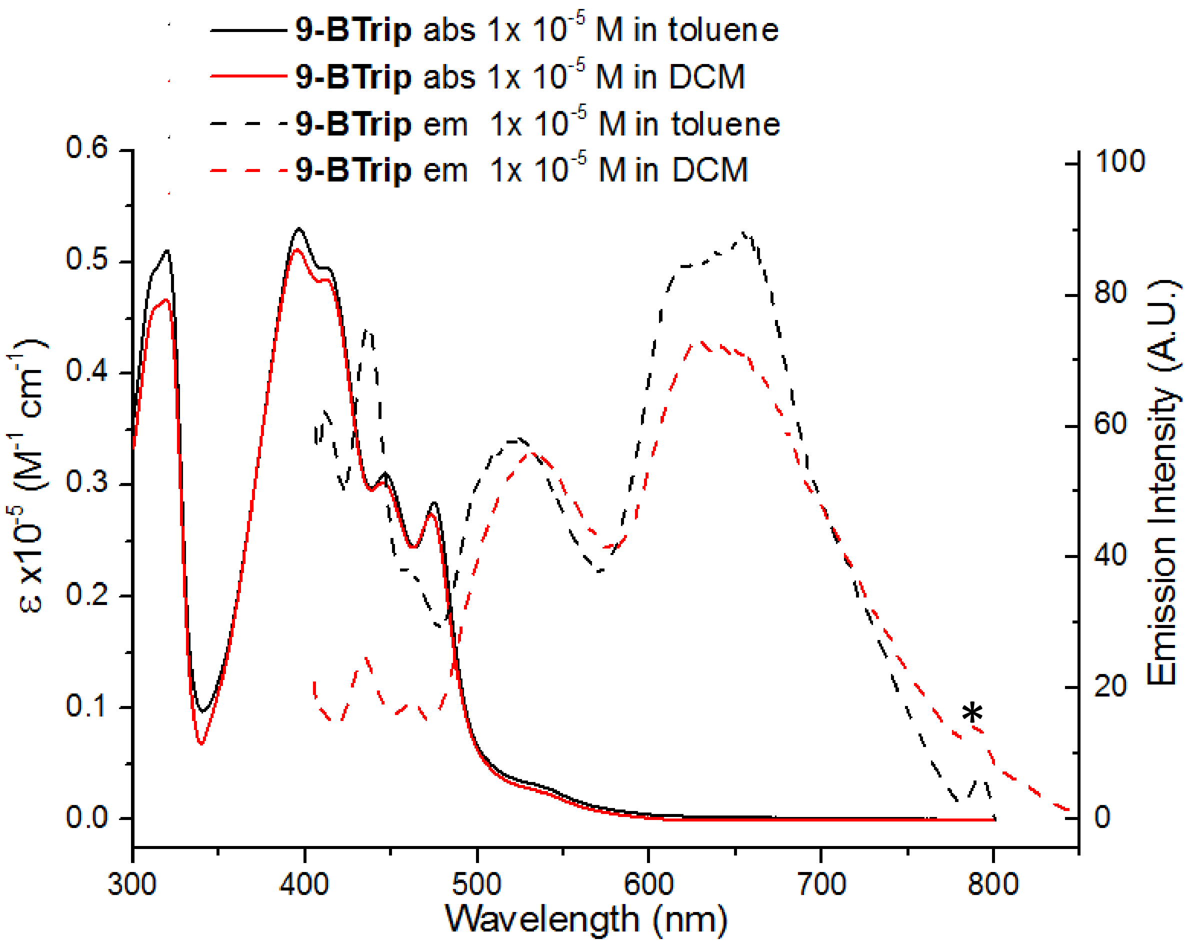

Figure S6: Absorbance and emission spectra of **9-BTrip** in toluene and DCM (1 x10^{-5}). λex = 396 nm. * = Half-harmonic

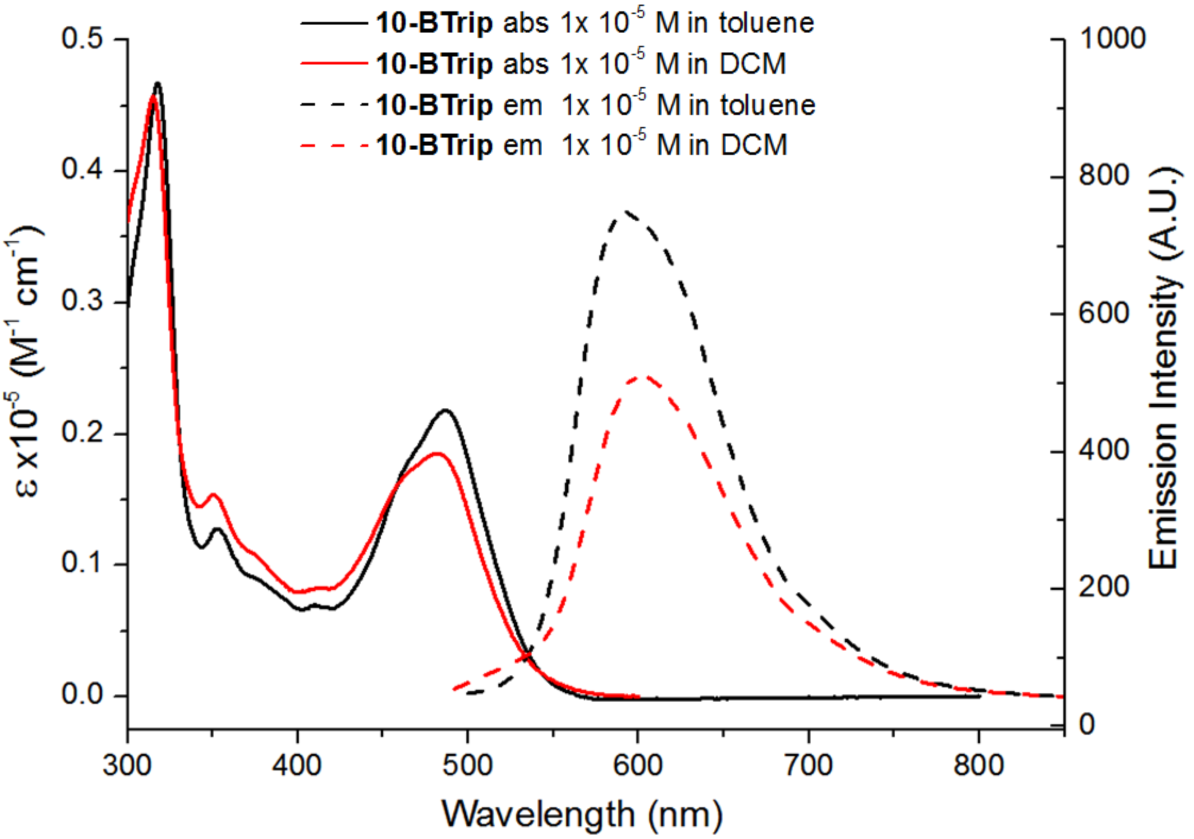

Figure S7: Absorbance and emission spectra of **10-BTrip** in toluene and DCM (1 x10^{-5}). λex = 487 nm.

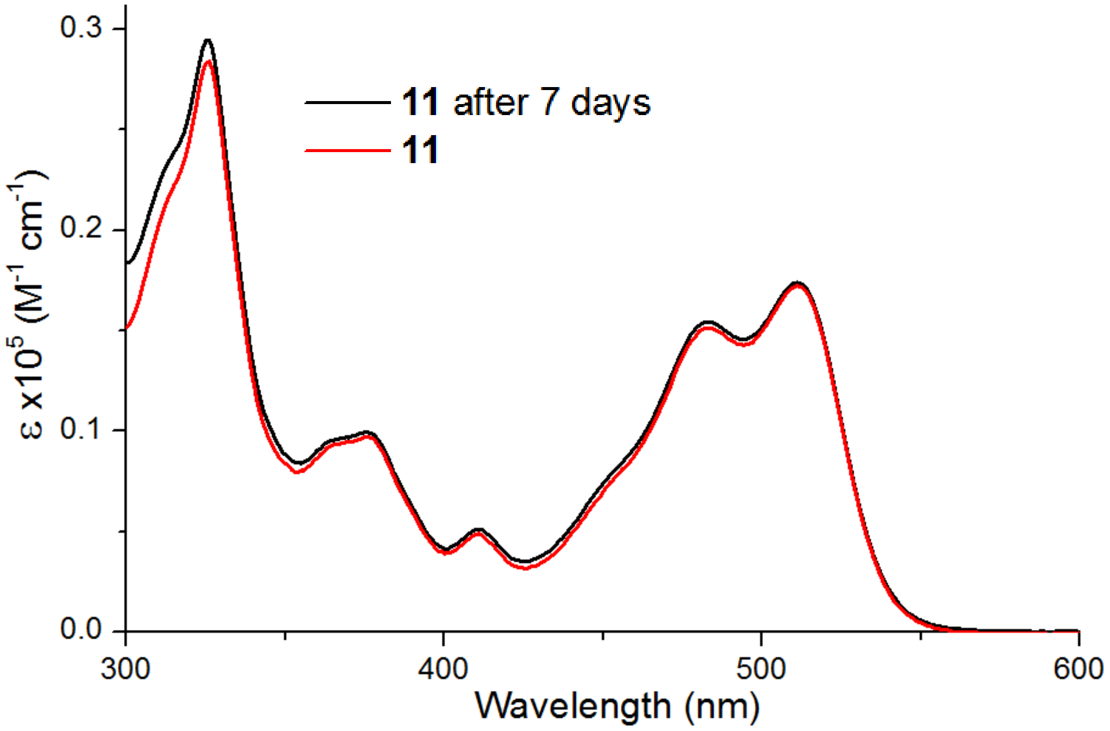

Figure S8: Stability in dilute solution under ambient conditions. Absorbance of **11** in toluene (1 x 10^{-5}) freshly made solution *vs* after 7 days.

Table S1: Summary of photophysical data.

Compound	λmax$_{abs}$ (nm)[a]	ε x 10^{-3} (M^{-1} cm^{-1})[a]	λmax$_{em}$ (nm)[a]	Optical band-gap (eV)[a]	QY (%)[b]
**1-BMes**	351, 367, 420	16.1, 12.9, 4.1	516[c]	2.61	16[c]
**2-BMes**	336, 370, 428	15.8, 8.7, 7.2	512[d]	2.59	24[d]
**6-BMes**	418[s], 378, 359	5.1, 14.7, 17.1	411, 433, 536[e]	2.54	7[e]
**7-BMes**	402, 374, 338	9.8, 10.2, 14.2	410, 478[f]	2.76	19[f]
**9-BTrip**	320, 396, 413, 446, 476	51.0, 53.0, 49.4, 31.0, 28.5	524, 628, 650[h]	2.49	1[h]
**10-BTrip**	318, 353, 487	46.5, 12.5, 21.5	592[i]	2.31	23[i]
**5**	310, 348, 431	11.6, 6.2, 3.3	486[j]	2.62	42[k]
**11**	326, 375, 411, 483, 511	28.4, 9.7, 4.9, 15.1, 17.2	553[l]	2.30	66[m]

[a]Measured at 1 x 10^{-5} M in toluene, [b]absolute quantum yield values measured using an integrating sphere at 0.2 x 10^{-5} M in toluene (estimated error ± 10%), [s]Shoulder, λex (nm): [c]351, [d]336, [e]359, [f]338, [g]355, [h]396, [i]487, [j]431, [k]348, [l]326, [m]483.

Electrochemical Properties

Table S2: Summary of electrochemical data

Compound	First Reduction Wave			
	E_{pa} (V)	$E_{1/2}$ (V)	E_{onset} (V)	LUMO (eV)
**1-BMes**	-2.00	-1.92	-1.84	-2.96
**2-BMes**	-2.00	-1.92	-1.85	-2.95
**6-BMes**	-1.84	-1.77	-1.70	-3.10
**7-BMes**	-1.80	-1.73	-1.62	-3.18
**9-BTrip**	-1.40	-1.33	-1.27	-3.53
**10-BTrip**	-146	-1.39	-1.32	-3.48
**5**	-2.10	N/A	-1.93	-2.87
**11**	-1.56	N/A	-1.42	-3.38
	Second Reduction Wave			
**1-BMes**	-2.72	N/A	-2.58	N/A
**2-BMes**	-2.65	N/A	-2.50	N/A
**6-BMes**	-2.60	N/A	-2.49	N/A
**7-BMes**	-2.50	N/A	-2.37	N/A
**9-BTrip**	-2.00	-1.93	-1.88	N/A
**10-BTrip**	-1.93	-1.86	-1.80	N/A
**5**	N/A	N/A	N/A	N/A
**11**	-1.89	N/A	-1.77	N/A

Measured in THF (1 mM) (compound **9** at ~0.5 mM) with $[^{n}Bu_4N][PF_6]$ (0.1 M) as the supporting electrolyte at a scan rate of 50 mV/s, potentials are given relative to Fc/Fc^+ redox couple which is taken to be 4.80 eV below vacuum.[S4] No oxidation waves are observed within the potential window on THF.

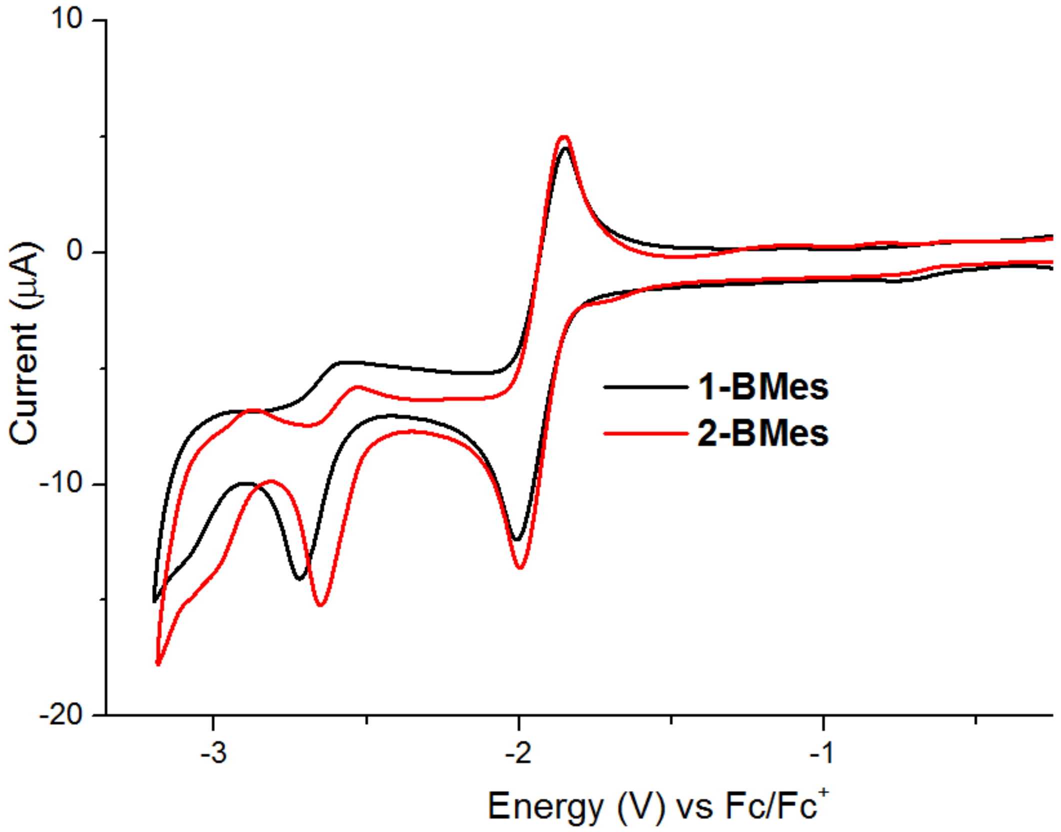

Figure S9: Cyclic voltammetry plots of **1-BMes** and **2-BMes**. Measured in THF (1 mM), with [^{n}Bu$_4$N][PF$_6$] (0.1 M) as the supporting electrolyte at a scan rate of 50 mV/s.

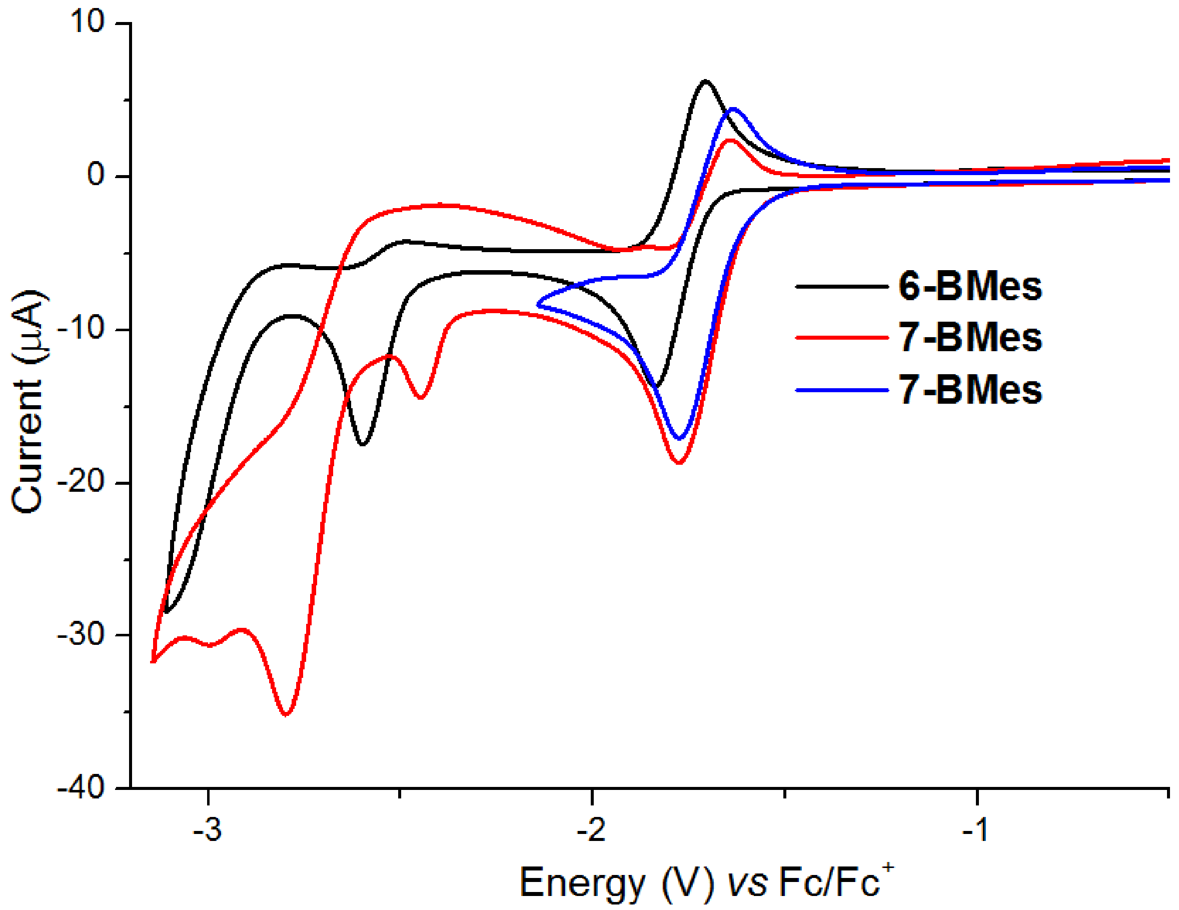

Figure S10: Cyclic voltammetry plots of **6-BMes** and **7-BMes**. Measured in THF (1 mM), with [$^{n}Bu_4N$][PF_6] (0.1 M) as the supporting electrolyte at a scan rate of 50 mV/s.

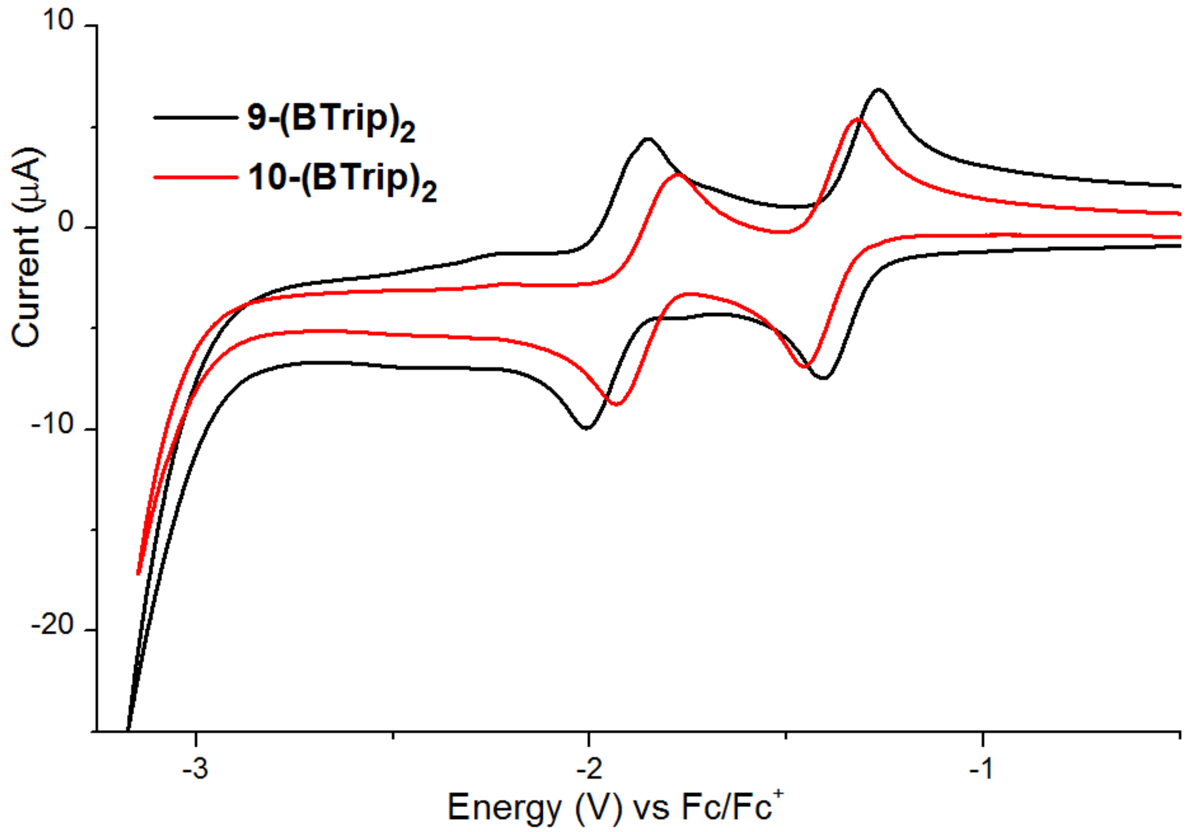

Figure S11: Cyclic voltammetry plots of **9-BTrip** and **10-BTrip**. Measured in THF (1 mM), with $[^{n}Bu_4N][PF_6]$ (0.1 M) as the supporting electrolyte at a scan rate of 50 mV/s.

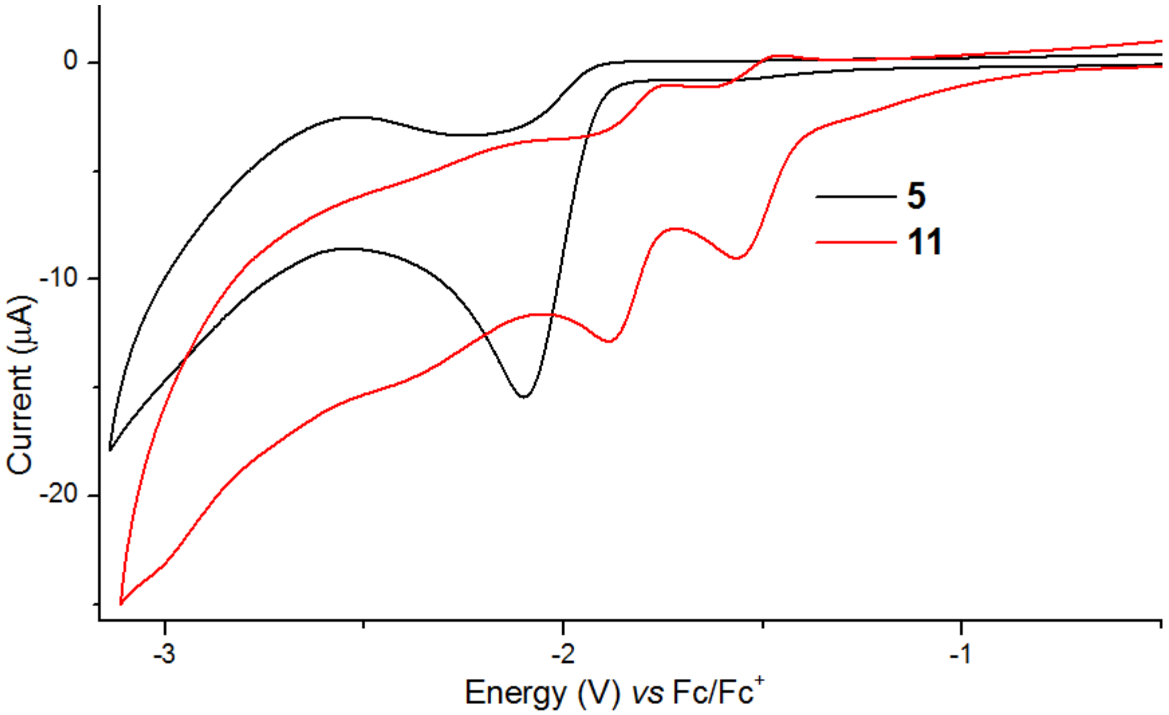

Figure S12: Cyclic voltammetry plots of **5** and **11**. Measured in THF (compound **5** = 1 mM, compound **11** = ~0.5 mM), with $[^{n}Bu_4N][PF_6]$ (0.1 M) as the supporting electrolyte at a scan rate of 50 mV/s.

Crystallographic Details

Data Collection. Synchrotron X-ray data were collected at beamline I19 (λ = 0.6889 Å) Diamond Light Source[S10] for **1-BCl**, **10-BTrip)** and **11** at temperature of 100 K. Data were measured using GDA suite of programs. X-ray data for compound **1-BMes** and **9-BCl** was collected at a temperature of 150 K using Mo-Kα radiation on an Agilent Supernova, equipped with an Oxford Cryosystems Cobra nitrogen flow gas system. Data were measured using CrysAlisPro suite of programs. X-ray data for compound **2-BMes**, **7-BMes** and **9-BMes** was collected at a temperature of 150 K using a using Mo-Kα radiation on an Agilent Supernova, equipped with an Atlas detector and Oxford Cryosystems Cobra nitrogen flow gas system. X-ray data for **6-BMes** was collected at 150 K on a Rigaku FR-X diffractometer using Mo-Kα radiation. Data were measured using CrysAlisPro suite of programs.

Crystal structure determinations and refinements. X-ray data were processed and reduced using dials (**1-BCl**, **11**) and CrysAlisPro (**10-BTrip, 1-BMes**, **9-BCl**, **2-BMes** and **9-BMes**, **6-BMes**, **7-BMes**) suite of programs. Absorption correction was performed using empirical methods based upon symmetry-equivalent reflections combined with measurements at different azimuthal angles. The crystal structures were solved with SHELXT structure solution program using intrinsic phasing, and refined with the Olex 2 (**1-BCl**, **10-BTrip, 1-BMes**, **9-BCl**, **2-BMes** and **9-BMes**) and SHELXL refinement packages (**6-BMes**, **7-BMes**, **11**) using Least Squares minimisation.[S11] All the atoms were refined anisotropically. Hydrogen atoms were placed in calculated positions refined using idealized geometries (riding model) and assigned fixed isotropic displacement parameters.

CCDC 1546328 (**1-BCl**), 1537995 (**1-BMes**), 1538224 (**2-BMes**), 1538258 (**9-BCl$_2$**), 1538386 (**9-BMes**), 1546329 (**10-BTrip**), 1553725 (**11**), 1553723 (**6-BMes**) and 1555383 (**7-BMes**) contains the supplementary crystallographic data for this paper. These data can be obtained free of charge *via* www.ccdc.cam.ac.uk/conts/retrieving.html (or from the Cambridge Crystallographic Data Centre, 12 Union Road, Cambridge CB21EZ, UK; fax: (+44)1223-336-033; or deposit@ccdc.cam.ac.uk).

Table S1**:** Crystallographic information for compounds **1-BCl**, **1-BMes**, **2-BMes**.

	**1-BCl**	**1-BMes**	**2-BMes**
Crystal colour	yellow	yellow	yellow
Crystal size (mm)	0.08 × 0.02 × 0.02	0.3 × 0.3 × 0.5	0.3 × 0.4 × 0.4
Crystal system	Orthorombic	Monoclinic	Monoclinic
Space group, *Z*	Pbca, *8*	P2$_1$/c, *4*	P2$_1$/c, *8*

a (Å)	35.8866(3)	12.3346(6)	19.8982(6), ,
b (Å)	10.32200(10)	13.3034(7)	9.9574(4)
c (Å)	7.75280(10)	13.4028(7)	21.3509(8)
α (°)	90	90	90
β (°)	90	106.114(5)	92.071(3)
γ (°)	90	90	90
V (Å^3)	2871.80(5)	2112.87(19)	4227.6(3)
Density (Mg.m^{-3})	1.390	1.208	1.201
Wavelength (Å)	0.6998	0.71703	0.71703
Temperature (K)	100	150	220
μ(Mo-Kα) (mm^{-1})	0.233	0.067	0.067
2Θ range (°)	4.4 to 49.038	6.878 to 50.054	6.856 to 52.744
Reflns collected	33859	7987	21510
Independent reflns (R_{int})	2635 (0.1049)	3729 (0.0231)	8622 (0.0432)
L.S. parameters, *p*	199	274	547
No. of restraints, *r*	0	0	0
$R1$ (F)[a] $I > 2.0σ(I)$	0.0478	0.0483	0.0626
$wR2(F^2)$,[a] all data	0.1405	0.1289	0.1761
$S(F^2)$,[a] all data	1.040	1.083	1.019
CCDC number	1546328	1537995	1538224

[a] $R1(F) = Σ(|F_o| − |F_c|)/Σ|F_o|$; [b] $wR^2(F^2) = [Σw(F_o^2 − F_c^2)^2/ΣwF_o^4]^{½}$; [c] $S(F^2) = [Σw(F_o^2 − F_c^2)^2/(n + r − p)]^{½}$

Table S2: Crystallographic information for compounds **6-BMes** and **7-BMes**.

	**6-BMes**	**7-BMes**
Crystal colour	yellow	yellow
Crystal size (mm)	0.6 × 0.3 × 0.15	0.3 × 0.3 × 0.2
Crystal system	Monoclinic	Monoclinic
Space group, *Z*	$P2_1/c$, *4*	$P2_1/c$, *4*
a (Å)	13.7785(4)	11.3745(19)
b (Å)	10.2018(2)	13.741(2)
c (Å)	20.9277(7)	13.0111(16)
α (°)	90	90
β (°)	108.461(4)	101.224(13)
γ (°)	90	90
V ($Å^3$)	2790.33(15)	1994.8(5)
Density ($Mg.m^{-3}$)	1.284	1.276
Wavelength (Å)	0.71703	0.71703
Temperature (K)	150	293
μ(Mo-Kα) (mm^{-1})	0.150	0.073
2Θ range (°)	4.296 to 58.352	6.71 to 57.9
Reflns collected	24784	9374
Independent reflns (R_{int})	6670 (0.0336)	4553 (0.0494)
L.S. parameters, *p*	365	274

No. of restraints, *r*	0	0
*R*1 (*F*)[a] *I* > 2.0σ*(I)*	0.0477	0.0853
*wR*2(F^2),[a] all data	0.1232	0.2168
S(F^2),[a] all data	1.056	1.050
CCDC number	1553723	1555383

Table S3: Crystallographic information for compounds **9-BMes**, **9-BMes**, **10-BTrip** and **11**.

	**9-BCl**	**9-BMes**	**10-BTrip**	**11**
Crystal colour	red	red	red	red
Crystal size (mm)	0.3 × 0.3 × 0.5	0.2 × 0.3 × 0.4	0.08 × 0.02 × 0.1	0.1 x 0.1 x 0.2
Crystal system	Triclinic	Monoclinic	Monoclinic	Monoclinic
Space group, *Z*	P-1, *2*	C2/c, *4*	$P2_1$/c, *4*	$P2_1$/c, *4*
a (Å)	7.1186(5)	18.2145(10)	9.2839(16)	9.59243(5)
b (Å)	8.2556(6)	11.8265(8)	23.623(6)	17.61337(9)
c (Å)	10.1532(8)	16.5516(9)	10.9585(17)	11.75177(6)
α (°)	103.751(6)	90	90	90
β (°)	95.819(6)	98.452(5)	90.503(14)	113.2421(4)
γ (°)	108.226(7)	90	90	90
V ($Å^3$)	540.43(7)	3526.7(4)	2403.3(8)	1824.385(13)
Density ($Mg.m^{-3}$)	1.453	1.206	1.112	1.297
Wavelength (Å)	0.71703	0.71703	0.71703	0.6889
Temperature (K)	150	293	100	100

μ(Mo-Kα) (mm^{-1})	0.320	0.067	0.057	0.069
2Θ range (°)	6.75 to 58	6.714 to 58.446	4.4 to 49.038	4.288 to 51.004
Reflns collected	4070	9158	28221	24396
Independent reflns (R_{int})	2459 (0.0259)	4040 (0.0492)	4399 (0.1827)	3727 (0.0529)
L.S. parameters, p	154	229	286	257
No. of restraints, r	0	0	0	0
$R1$ (F)[a] $I > 2.0\sigma(I)$	0.0452	0.0631	0.1081	0.0520
$wR2(F^2)$,[a] all data	0.1126	0.1550	0.2952	0.1744
$S(F^2)$,[a] all data	1.060	1.028	1.041	1.227
CCDC number	1538258	1538386	1546329	1553725

[a] $R1(F) = \Sigma(|F_o| - |F_c|)/\Sigma|F_o|$; [b] $wR^2(F^2) = [\Sigma w(F_o^2 - F_c^2)^2/\Sigma wF_o^4]^{½}$; [c] $S(F^2) = [\Sigma w(F_o^2 - F_c^2)^2/(n + r - p)]^{½}$

Extended Packing Structures for B_2-PAHs

2-BMes

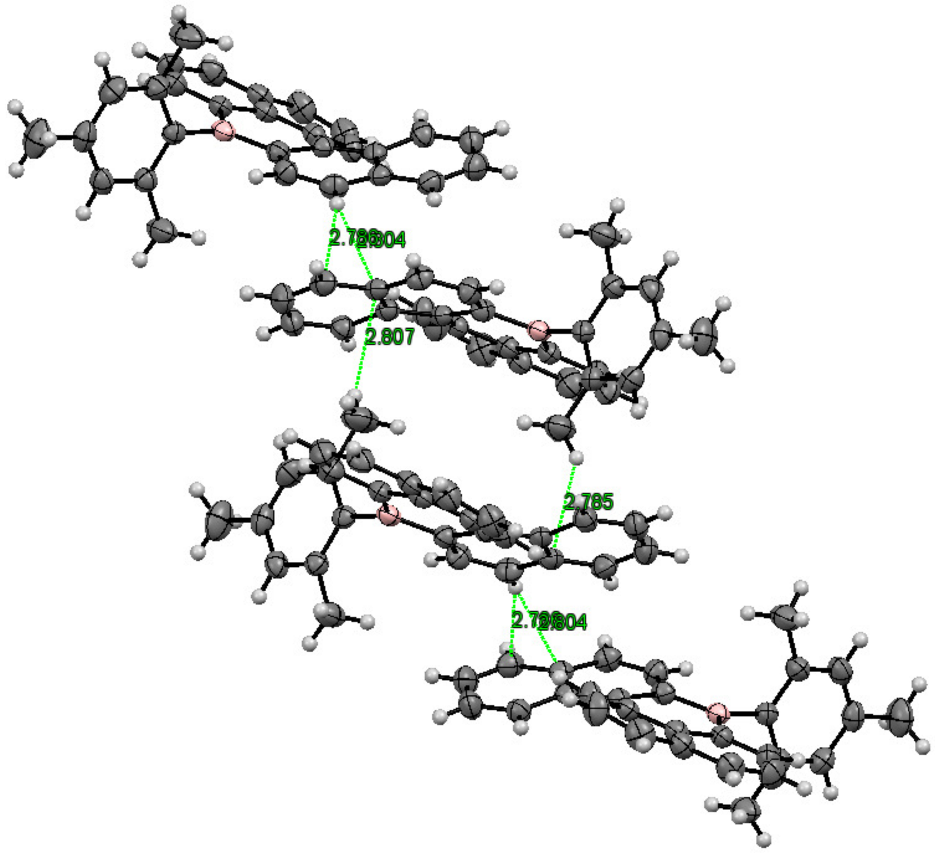

Figure S13: Extended packing structure of 2-BMes showing the closest intermolecular contacts which are C-H---π

9-BCl

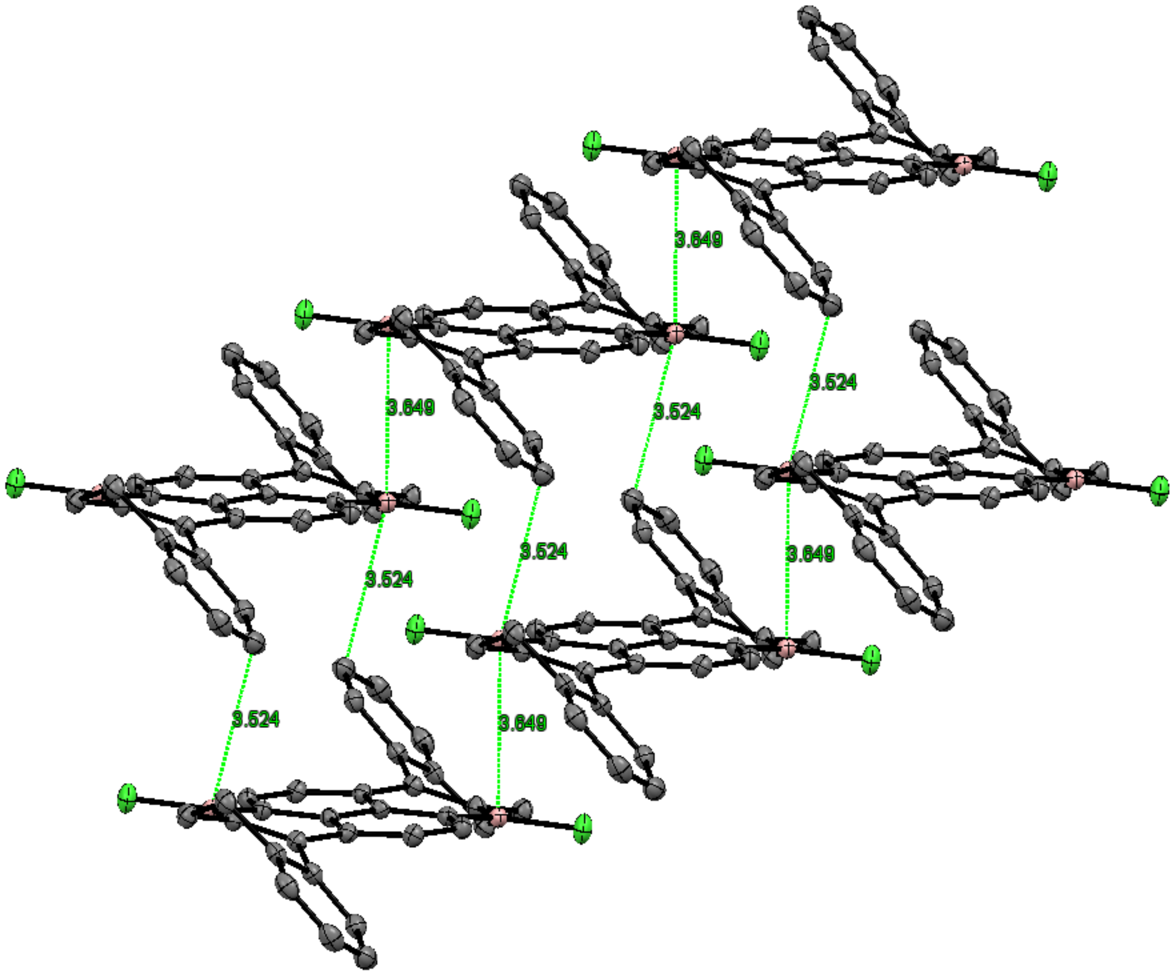

Figure S14: Extended packing structure (hydrogens omitted for clarity) for 9-BCl showing the close B---B and B---C intermolecular contacts.

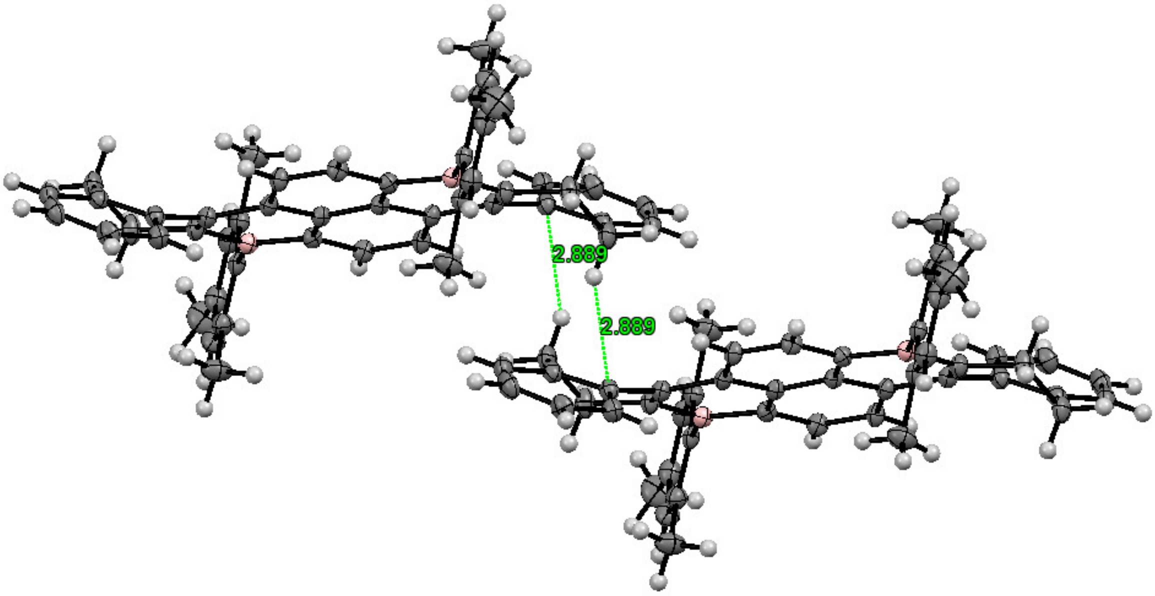

Figure S15: Extended packing structure for 9-BMes showing the closest intermolecular contacts which are C-H---π.

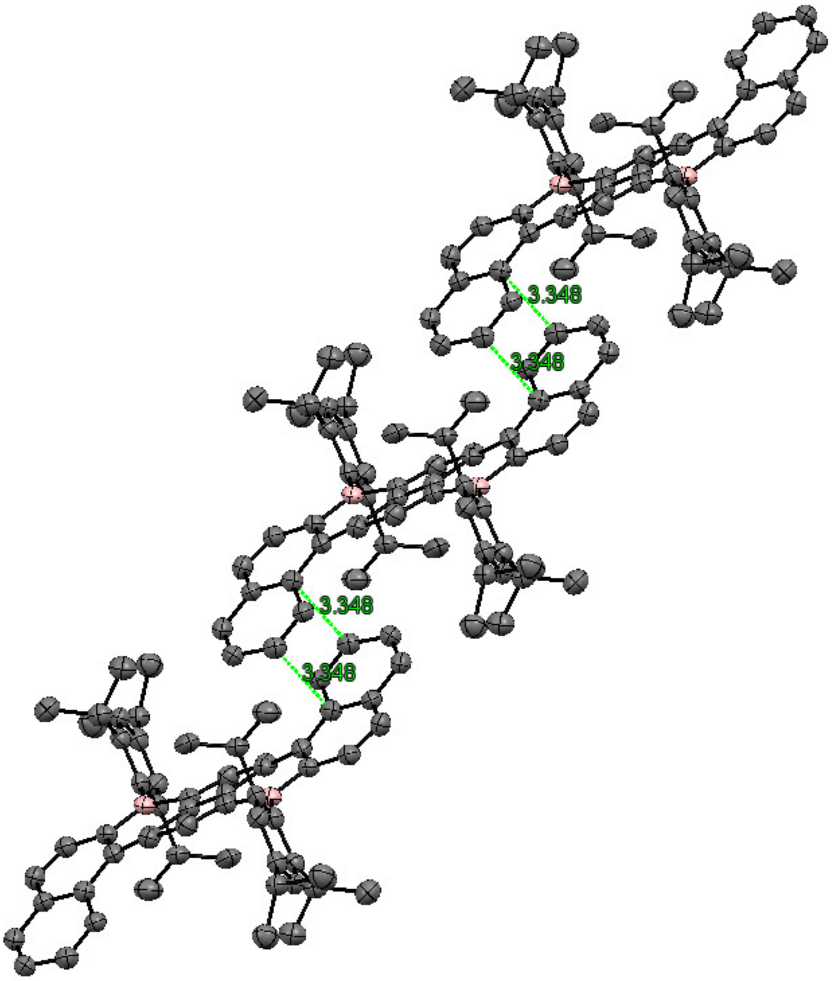

Figure S16: Extended packing structure (hydrogens omitted for clarity) for 10-BTrip showing the close C---C intermolecular contacts involving ring A moieties in adjacent molecules.

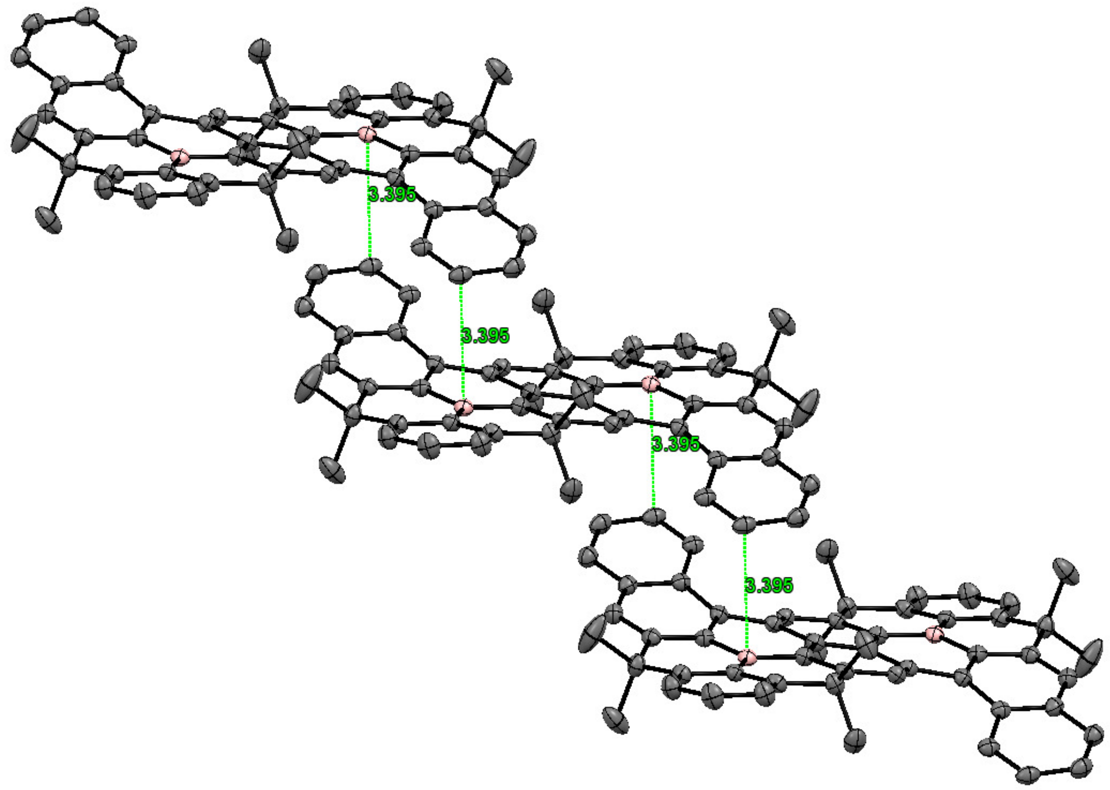

Figure S17: Extended packing structure (hydrogens omitted for clarity) for 11 showing the close B---C and C---C intermolecular contacts involving the diborapyrene core and the ring A of an adjacent molecule.

DFT Calculations

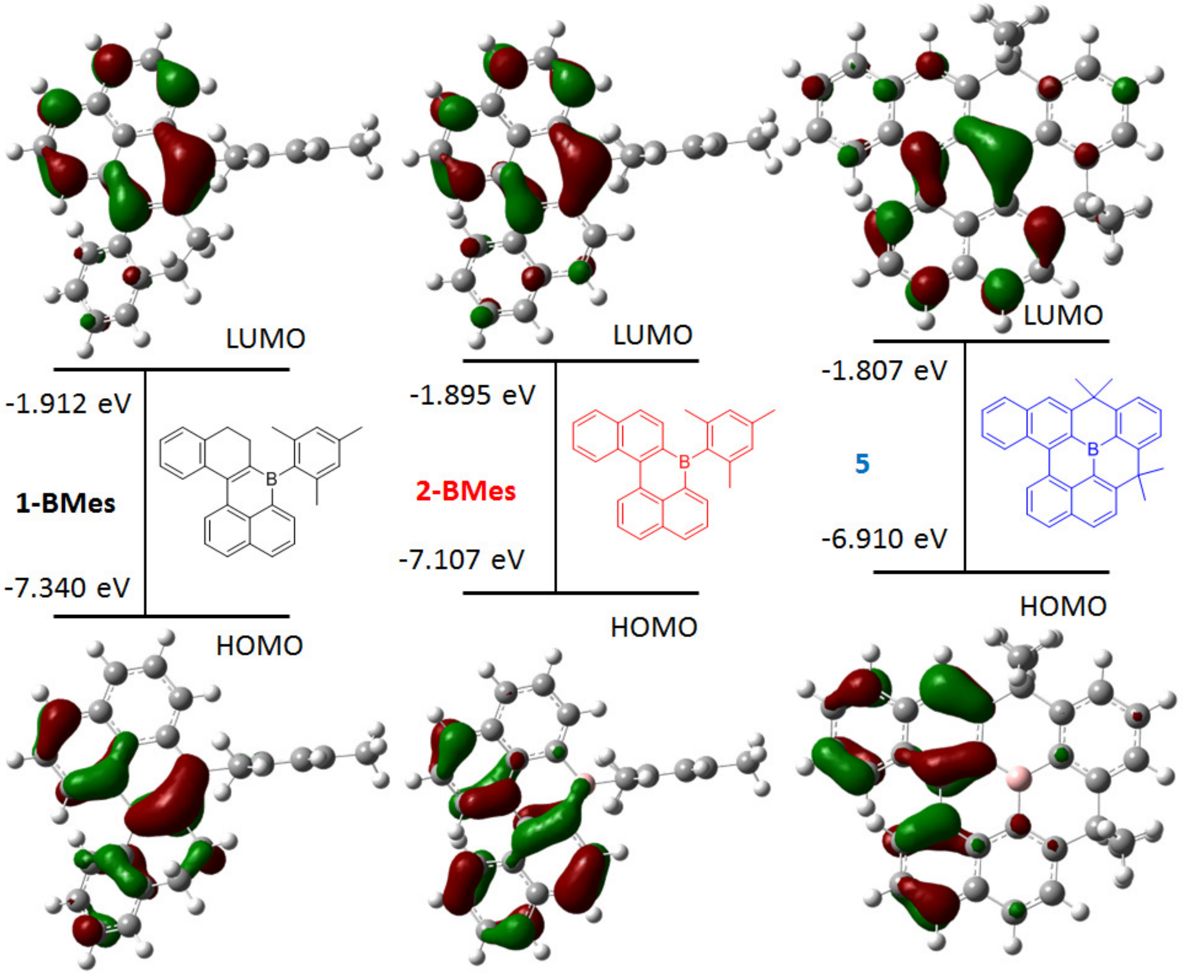

Figure S18: Molecular orbital energy levels and molecular orbital contours (isovalue = 0.04) of the HOMO and LUMO of **1-BMes** (left), **2-BMes** (centre), **5** (right).

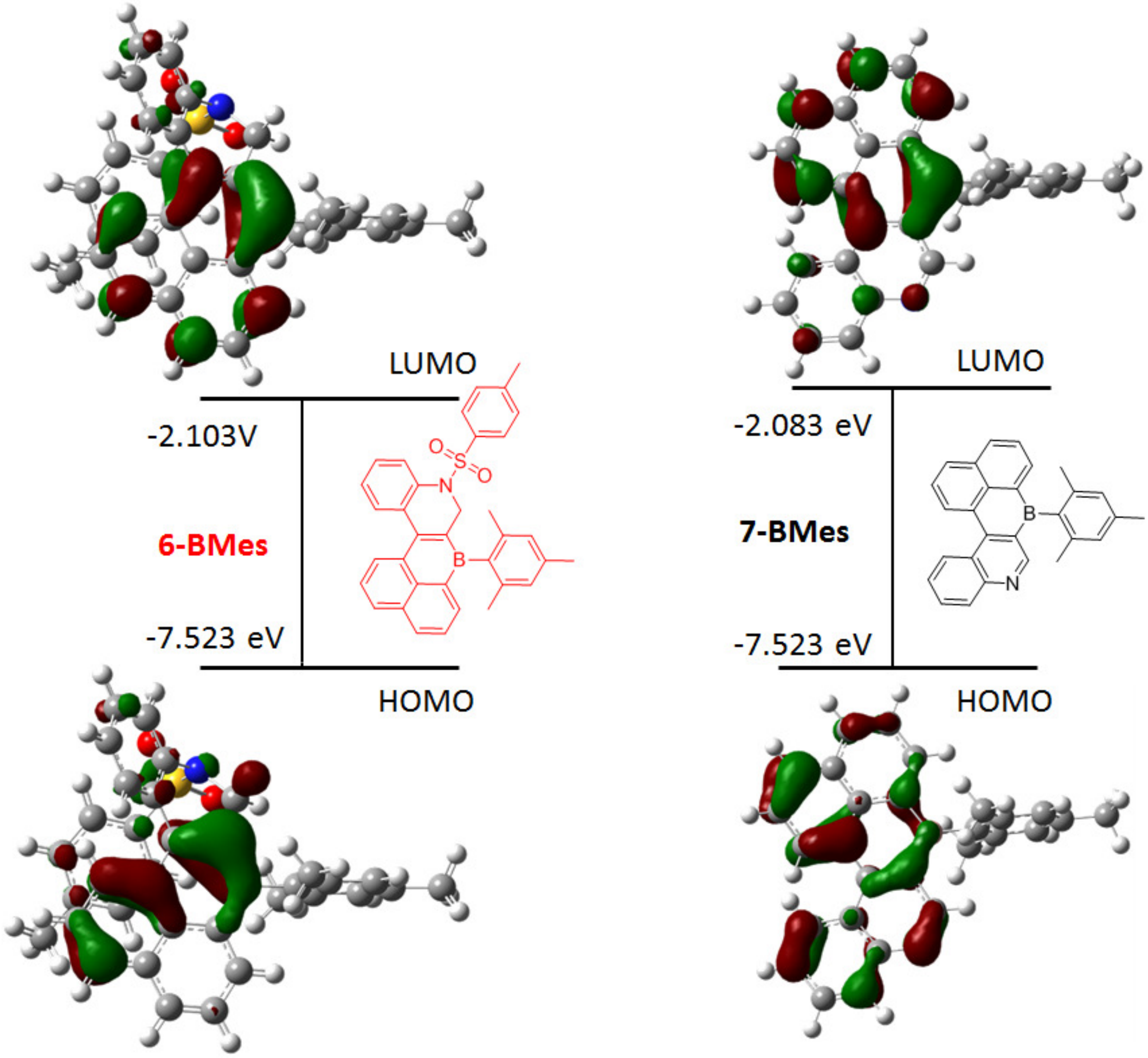

Figure S19: Molecular orbital energy levels and molecular orbital contours (isovalue = 0.04) of the HOMO and LUMO of **6-BMes** (left), **7-BMes** (right).

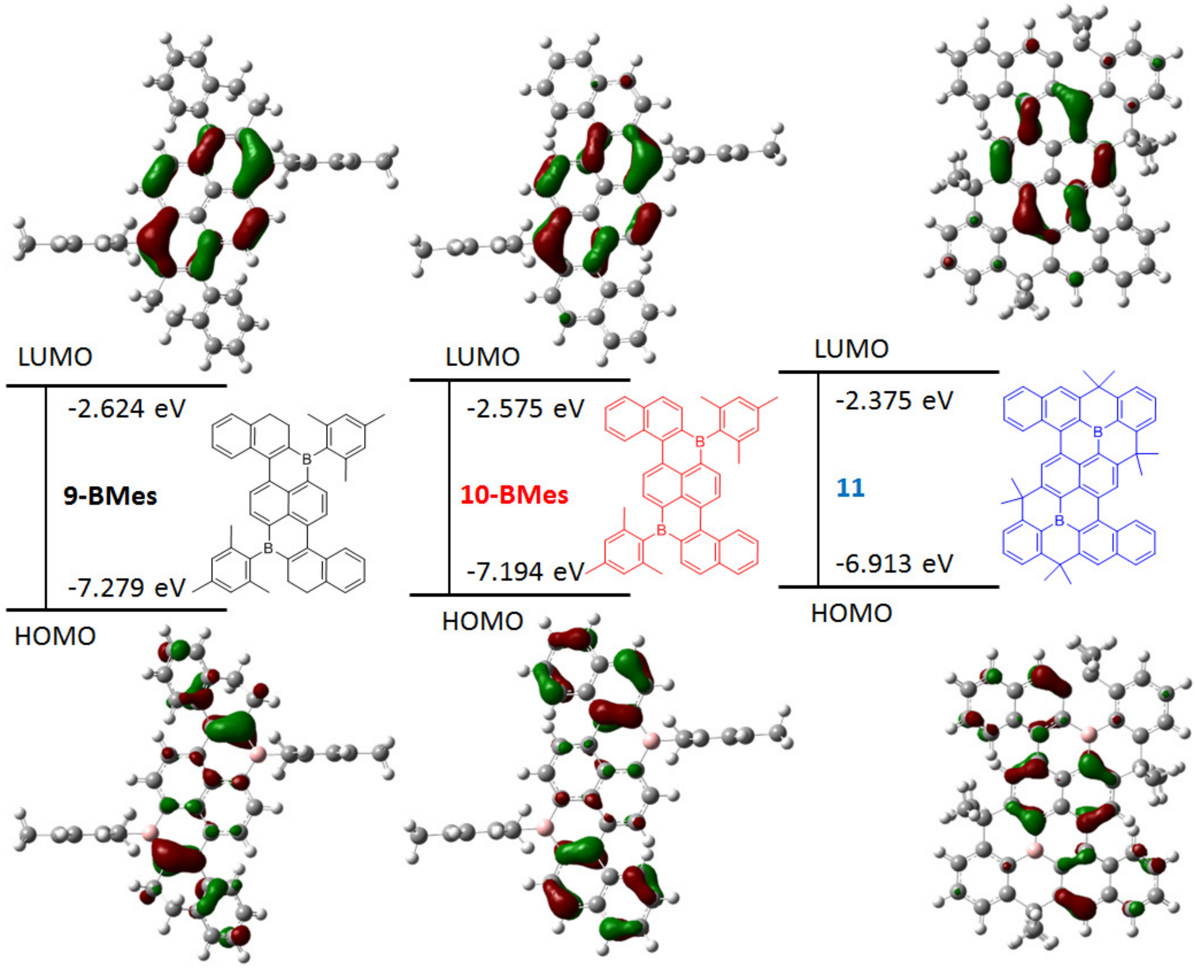

Figure S20: Molecular orbital energy levels and molecular orbital contours (isovalue = 0.04) of the HOMO and LUMO of **9-BMes** (left), **10-BMes** (centre) and **11** (right).

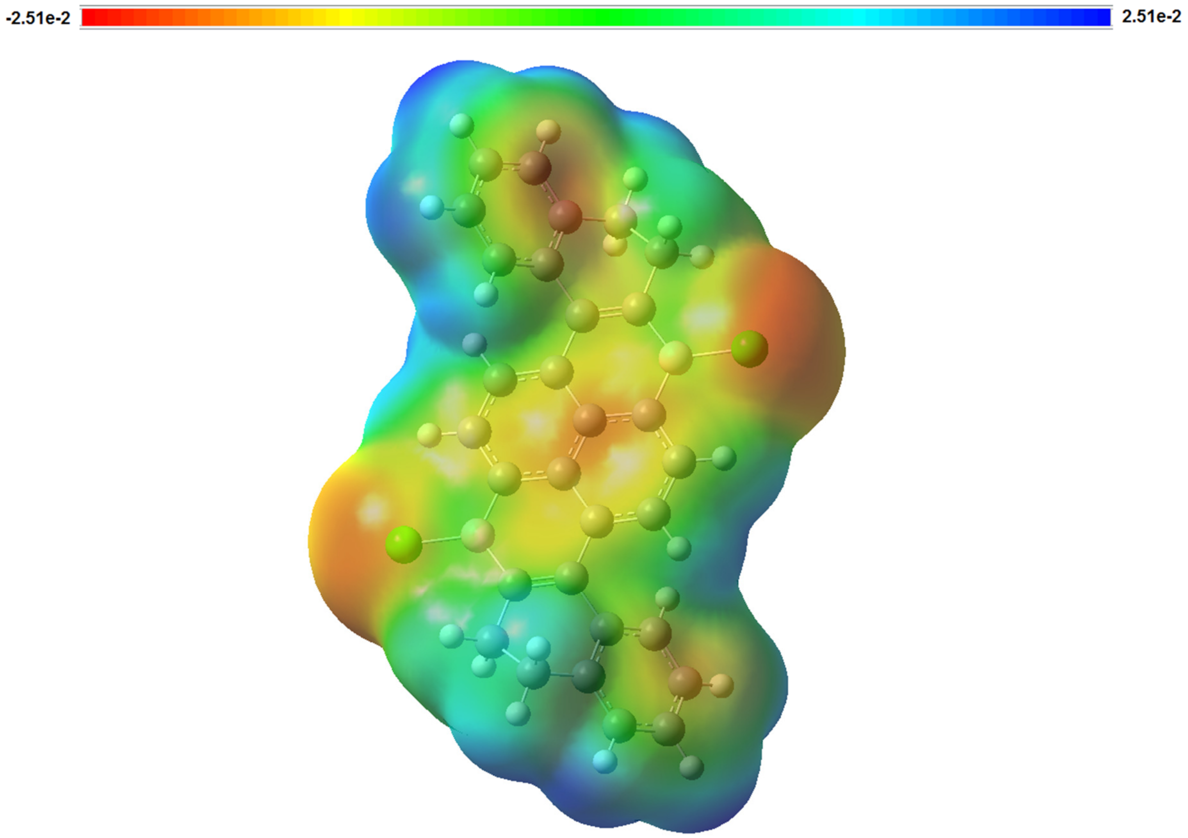

Figure S21: Electrostatic potential surface of **9-BCl**.

The unexpected packing structure for 9-BCl aligns adjacent B atoms at close distances, but this is not positioning highly positive areas close in space, instead the electrostatic potential near boron is close to neutral.

Optimised Structure Coordinates

1-BMes Energy = -1144.850658 A.U

	X	Y	Z
C	-3.517400	-2.707300	-0.714500
C	-2.332300	-2.610100	0.008000
C	-1.742200	-1.373200	0.247000
C	-2.369800	-0.200000	-0.211700
C	-3.532600	-0.315500	-0.983200
C	-4.105200	-1.557500	-1.230200
H	0.101300	-2.201600	0.961800
H	-3.964100	-3.677900	-0.896100
H	-1.845400	-3.507400	0.374900
C	-0.402600	-1.234400	0.919300
C	-1.692100	1.101800	0.035900
H	-3.981400	0.570400	-1.415100
H	-5.004400	-1.624200	-1.830800
C	-0.324900	1.116000	0.095000
C	0.413200	-0.204400	0.139800
H	1.395800	-0.057800	0.595200
C	-2.485300	2.340600	0.191700
C	-1.854400	3.624300	0.114400
C	-3.832000	2.296200	0.513000
C	-0.441900	3.738200	-0.045800
C	-2.641800	4.799300	0.241500
C	-4.605200	3.465000	0.666900
H	-4.316000	1.341200	0.668700

C	0.122200	5.002700	-0.125500
C	-2.016100	6.066400	0.129100
C	-4.030900	4.696800	0.504700
H	-5.657000	3.375500	0.909100
C	-0.660400	6.171300	-0.060100
H	1.200300	5.088800	-0.231000
H	-2.632000	6.956600	0.205200
H	-4.617800	5.604200	0.598200
H	-0.192500	7.145200	-0.139400
B	0.421500	2.456200	-0.021700
C	1.990100	2.506400	-0.158800
C	2.802600	2.459800	0.984900
C	2.598700	2.581500	-1.424100
C	4.191900	2.490100	0.854600
C	3.987500	2.607000	-1.526000
C	4.802900	2.561100	-0.394500
H	4.809600	2.458200	1.748100
H	4.447100	2.663800	-2.509400
C	2.166600	2.395800	2.353300
H	1.597400	3.307000	2.561500
H	1.464900	1.558000	2.423100
H	2.916100	2.277500	3.136800
C	6.304300	2.569600	-0.532000
H	6.787500	2.726100	0.433200
H	6.662500	1.619400	-0.937400
H	6.631600	3.358700	-1.212500

C	1.741700	2.630200	-2.667300
H	1.055200	1.778400	-2.702300
H	1.128900	3.536300	-2.684200
H	2.351100	2.613200	-3.571600
H	-0.531400	-0.878000	1.949000
H	0.594100	-0.574000	-0.879400

2-BMes Energy = -1143.6529263 A.U

	**X**	**Y**	**Z**
C	-3.612200	-2.664700	-0.804500
C	-2.303600	-2.614700	-0.405600
C	-1.659100	-1.374100	-0.167700
C	-2.403600	-0.161500	-0.260900
C	-3.736800	-0.252000	-0.751500
C	-4.322400	-1.463400	-1.016400
H	0.282200	-2.266500	0.171500
H	-4.091000	-3.617500	-0.996100
H	-1.725000	-3.525400	-0.295200
C	-0.262500	-1.331900	0.093400
C	-1.737600	1.084000	0.039700
H	-4.286100	0.652400	-0.971600
H	-5.331800	-1.497300	-1.408000
C	-0.342300	1.098900	0.119900
C	0.376700	-0.130600	0.157700
H	1.457000	-0.088600	0.256600
C	-2.491000	2.339700	0.274100
C	-1.851600	3.615600	0.129100

C	-3.790900	2.323600	0.751400
C	-0.444900	3.719800	-0.091200
C	-2.619300	4.802200	0.278900
C	-4.536900	3.504500	0.943800
H	-4.257400	1.382000	1.006800
C	0.122000	4.975500	-0.243500
C	-1.994600	6.059600	0.080500
C	-3.980200	4.723300	0.667200
H	-5.555600	3.432700	1.304300
C	-0.652200	6.150100	-0.191100
H	1.196100	5.049700	-0.388000
H	-2.600100	6.956100	0.165200
H	-4.550400	5.638900	0.779400
H	-0.185700	7.117700	-0.331200
B	0.416900	2.441800	-0.000500
C	1.988000	2.490700	-0.092400
C	2.765200	2.739500	1.049100
C	2.632100	2.280100	-1.324400
C	4.156700	2.775700	0.948300
C	4.022300	2.326200	-1.397200
C	4.803500	2.570700	-0.267700
H	4.747400	2.970400	1.839400
H	4.509300	2.167900	-2.356000
C	2.090500	2.965600	2.381300
H	1.465400	3.863400	2.355800
H	1.437200	2.124900	2.635300

H	2.820500	3.082200	3.183000
C	6.307900	2.590400	-0.366100
H	6.755900	3.025900	0.527900
H	6.702500	1.577100	-0.479900
H	6.636400	3.167900	-1.232700
C	1.812700	1.995800	-2.560900
H	1.295600	1.035200	-2.470000
H	1.045800	2.762200	-2.708800
H	2.437800	1.962100	-3.453900

5 Energy = -1259.1823933

	X	Y	Z
C	2.903900	-5.622800	-3.937100
C	3.223400	-4.674000	-2.928900
C	2.404800	-6.860200	-3.629500
C	3.607500	-3.351100	-3.250600
C	3.098400	-5.069100	-1.575200
C	2.165000	-7.229300	-2.277700
H	2.166500	-7.541100	-4.437700
C	3.798300	-2.442600	-2.242800
C	3.447700	-4.152700	-0.523600
C	2.539000	-6.346100	-1.277800
C	3.708100	-2.842300	-0.894100
H	3.828700	-2.082800	-0.134100
C	3.416800	-4.568400	0.906100
C	4.118400	-3.850800	1.936000
C	2.680700	-5.701900	1.266900
C	5.187000	-2.936400	1.707400

C	3.791000	-4.148600	3.291700
C	2.437500	-6.054500	2.626500
C	5.794400	-2.272800	2.740800
H	5.566700	-2.799400	0.705000
C	4.406100	-3.410700	4.338900
C	2.918800	-5.220900	3.604800
C	5.372500	-2.480100	4.073400
H	6.617500	-1.598800	2.536100
H	4.114700	-3.627200	5.361100
H	2.706600	-5.395600	4.654300
H	5.845200	-1.934800	4.881500
B	2.235300	-6.665500	0.174000
C	1.536900	-7.968400	0.529400
C	1.243800	-8.248800	1.877000
C	1.186700	-8.857200	-0.505700
C	0.569300	-9.433200	2.174500
C	0.534600	-10.043600	-0.168600
C	0.225500	-10.317900	1.158600
H	0.304700	-9.685200	3.194800
H	0.253300	-10.763600	-0.928000
H	-0.292500	-11.237600	1.405900
C	1.629600	-7.303100	3.027200
C	1.488900	-8.571900	-1.985800
C	0.327200	-6.823500	3.707600
H	-0.264000	-7.673100	4.055000
H	0.553800	-6.191600	4.568800

H	-0.278200	-6.245800	3.005300
C	2.468200	-8.112300	4.043400
H	3.398800	-8.451400	3.582500
H	2.718100	-7.507100	4.915500
H	1.919900	-8.987100	4.394600
C	0.150400	-8.607200	-2.761200
H	-0.519200	-7.824700	-2.397100
H	0.309500	-8.455000	-3.829500
H	-0.344300	-9.570600	-2.633900
C	2.417600	-9.690600	-2.513500
H	2.628500	-9.553100	-3.575600
H	3.364300	-9.685000	-1.968700
H	1.951500	-10.669100	-2.386700
H	3.700900	-3.062300	-4.291800
H	3.043100	-5.338300	-4.975100
H	4.024800	-1.408100	-2.470800

6-BMes Energy = -1979.7799585 A.U

	**X**	**Y**	**Z**
S	7.783800	5.217900	11.149800
O	7.448600	4.742600	12.479700
O	8.703900	4.470100	10.308300
N	6.327300	5.272200	10.306900
C	6.132200	7.807900	9.193500
C	7.350600	9.596900	7.904700
C	6.569900	9.207400	9.040800
C	7.805000	10.938500	7.797900

C	6.641500	6.813400	8.406100
C	7.708200	8.654100	6.896000
C	7.623400	6.078800	5.983500
C	5.185800	7.417500	10.266900
C	5.341100	6.151200	10.857800
C	6.665400	5.886800	4.969500
C	8.394700	6.880200	11.321100
C	9.274200	7.384700	10.368300
H	9.609500	6.755400	9.553300
C	4.102200	8.200800	10.681800
H	3.901800	9.142100	10.185400
C	8.454400	9.085700	5.808800
H	8.731700	8.362600	5.046800
C	8.788800	5.297900	5.973800
C	8.558400	11.327100	6.661400
H	8.888100	12.358200	6.586800
C	8.873000	10.422500	5.677600
H	9.451700	10.729700	4.815000
C	8.042100	4.153600	3.961000
C	8.984300	4.351000	4.966000
H	9.894000	3.757000	4.966700
C	6.362300	10.140000	10.043800
H	5.860900	9.849300	10.956500
C	6.829800	11.465000	9.941700
H	6.638600	12.158100	10.751900
C	7.520200	11.867500	8.830100

H	7.874200	12.888300	8.732600
C	3.273100	7.776100	11.711500
H	2.439700	8.396500	12.017700
C	4.529400	5.730900	11.903800
H	4.705000	4.757100	12.342900
C	6.884700	4.933100	3.979400
H	6.138000	4.793500	3.201900
C	6.384600	5.391300	8.835300
H	5.425100	5.011500	8.466200
H	7.161600	4.721200	8.467800
C	3.504200	6.557300	12.345300
H	2.862900	6.234300	13.156000
C	9.708000	8.696300	10.482000
H	10.389200	9.099000	9.739800
C	9.281300	9.506900	11.538900
C	7.943700	7.664100	12.376800
H	7.261200	7.251300	13.110400
C	5.405700	6.720900	4.955800
H	4.868600	6.638100	5.905800
H	4.732500	6.409200	4.156500
H	5.641000	7.779600	4.811600
C	8.399500	8.973500	12.480400
H	8.057700	9.591400	13.303400
C	9.827700	5.479500	7.054700
H	10.079800	6.537600	7.179500
H	10.745600	4.939300	6.819300

H	9.460900	5.106400	8.017700
C	8.250800	3.121400	2.882000
H	9.246300	2.680500	2.945400
H	8.133100	3.563000	1.889800
H	7.517500	2.315600	2.970000
B	7.355400	7.163800	7.094100
C	9.786600	10.918700	11.662300
H	10.780100	10.925600	12.119300
H	9.125500	11.523100	12.284300
H	9.865600	11.387700	10.680200

7-BMes Energy = -1159.69689 A.U

	**X**	**Y**	**Z**
C	-3.531500	-2.688100	-0.761500
C	-2.212800	-2.585400	-0.407000
C	-1.613700	-1.319100	-0.199600
C	-2.411900	-0.141100	-0.270400
C	-3.755500	-0.283200	-0.713100
C	-4.298900	-1.519200	-0.952700
H	-3.977000	-3.660500	-0.934200
H	-1.576400	-3.456300	-0.309300
C	-1.768200	1.113500	0.020300
H	-4.345600	0.598300	-0.919900
H	-5.318600	-1.597300	-1.309400
C	-0.374900	1.121200	0.067400
C	0.305800	-0.130000	0.077000
H	1.391100	-0.118700	0.167100
C	-2.512900	2.367300	0.265100

C	-1.857600	3.636000	0.128100
C	-3.815300	2.354800	0.736100
C	-0.449800	3.732300	-0.093500
C	-2.617000	4.827000	0.284600
C	-4.550200	3.540000	0.936500
H	-4.288300	1.415200	0.985400
C	0.124200	4.986200	-0.235900
C	-1.984500	6.082000	0.096200
C	-3.978800	4.754900	0.670100
H	-5.570100	3.476500	1.294300
C	-0.641500	6.165500	-0.174000
H	1.198600	5.054100	-0.381500
H	-2.584400	6.981500	0.187100
H	-4.540900	5.674800	0.788900
H	-0.168400	7.130800	-0.306900
B	0.407300	2.449800	-0.025100
C	1.978400	2.483800	-0.105200
C	2.747500	2.720900	1.044600
C	2.630500	2.276800	-1.333600
C	4.139800	2.747700	0.954900
C	4.021500	2.314100	-1.394500
C	4.794900	2.545700	-0.257100
H	4.724400	2.932300	1.851900
H	4.515100	2.158700	-2.350300
C	2.064400	2.945100	2.372700
H	1.446400	3.847700	2.347600

H	1.403500	2.107900	2.618700
H	2.789500	3.052100	3.180100
C	6.300000	2.555000	-0.342700
H	6.743500	2.984300	0.556500
H	6.687800	1.539000	-0.456400
H	6.639900	3.132700	-1.204800
C	1.820300	2.008100	-2.579600
H	1.302000	1.046600	-2.507000
H	1.055500	2.777000	-2.725200
H	2.452700	1.984300	-3.467700
N	-0.263300	-1.302600	0.021700

9-BCl Energy = -2125.3059523

	**X**	**Y**	**Z**
C	-1.700000	-1.782800	-0.516400
C	-0.446700	-2.412300	-0.546200
C	0.717200	-1.720000	-0.277300
C	0.633800	-0.318200	-0.034100
C	-0.633800	0.318200	0.034000
C	-1.828500	-0.450000	-0.163500
C	1.828500	0.450100	0.163400
C	1.700000	1.782800	0.516200
C	0.446700	2.412400	0.546000
C	-0.717200	1.720000	0.277200
B	-2.099800	2.386300	0.162900
C	-3.333000	1.534600	-0.115100
C	-3.171800	0.177200	-0.081400

B	2.099800	-2.386300	-0.163000
C	3.333000	-1.534600	0.115100
C	3.171700	-0.177200	0.081300
C	4.721300	-2.111800	0.260700
C	5.588400	-1.180700	1.106200
C	5.601300	0.173800	0.452300
C	4.388300	0.664700	-0.065900
C	-4.721400	2.111800	-0.260600
C	-5.588400	1.180700	-1.106100
C	-5.601300	-0.173900	-0.452200
C	-4.388300	-0.664700	0.066000
C	6.768300	0.912500	0.291700
C	6.760100	2.114100	-0.409300
C	5.577800	2.570400	-0.981700
C	4.402600	1.848900	-0.814000
C	-6.768300	-0.912500	-0.291500
C	-6.760100	-2.114100	0.409500
C	-5.577700	-2.570400	0.981900
C	-4.402500	-1.848900	0.814100
H	-2.576800	-2.365300	-0.763500
H	-0.402300	-3.473200	-0.766400
H	2.576800	2.365300	0.763300
H	0.402300	3.473200	0.766200
H	4.657100	-3.100400	0.719000
H	5.177800	-2.251200	-0.728400
H	6.605000	-1.563800	1.204900

H	5.158400	-1.109500	2.112400
H	-5.177800	2.251200	0.728500
H	-4.657200	3.100400	-0.718900
H	-5.158600	1.109500	-2.112300
H	-6.605100	1.563800	-1.204700
H	7.696400	0.530200	0.703100
H	7.678400	2.676200	-0.531400
H	5.569600	3.481900	-1.566800
H	3.495200	2.196900	-1.291800
H	-7.696500	-0.530200	-0.702800
H	-7.678300	-2.676200	0.531700
H	-5.569400	-3.481900	1.566900
H	-3.495100	-2.196900	1.291800
Cl	2.279000	-4.142800	-0.437400
Cl	-2.279000	4.142800	0.437300

9-BMes Energy = -1903.8810903 A.U

	**X**	**Y**	**Z**
C	1.465600	3.630900	0.305500
C	6.099000	1.751800	1.580900
H	6.903900	1.221700	2.071800
C	3.773000	2.469100	1.644600
C	4.856400	1.840600	2.229100
H	4.750000	1.419600	3.224700
C	1.312300	3.402500	1.644100
C	2.235000	2.155600	3.872900
C	3.922700	2.981600	0.324400

B	2.444100	2.691300	2.408700
C	2.799500	3.572500	-0.344500
C	0.247700	3.921600	-0.498400
C	-0.830400	4.563400	0.140400
C	-0.688900	4.883700	1.604100
H	-1.664900	5.105800	2.038800
H	-0.052100	5.766800	1.739200
C	0.077700	3.464900	-1.811200
H	0.865600	2.897100	-2.290500
C	1.840000	1.211200	6.501800
C	1.660900	0.370500	5.404900
H	1.358600	-0.660200	5.569800
C	2.412300	3.012300	4.971800
C	-1.104100	3.703600	-2.502200
H	-1.216400	3.338800	-3.515900
C	2.214500	2.532900	6.265800
H	2.353100	3.205700	7.107900
C	-2.139500	4.399300	-1.887500
H	-3.060200	4.595500	-2.424100
C	-2.001800	4.813000	-0.566400
H	-2.822200	5.317700	-0.066900
C	-0.026200	3.688500	2.286300
H	0.112700	3.874000	3.354300
H	-0.678800	2.807500	2.207000
C	2.835800	4.444000	4.743400
H	3.843300	4.488100	4.318200

H	2.835900	5.014000	5.673000
H	2.167800	4.946000	4.035600
C	1.652600	-0.111300	2.930700
H	0.944000	0.308900	2.209900
H	1.271300	-1.079600	3.256700
H	2.593400	-0.277100	2.397500
C	1.854800	0.823000	4.100400
C	1.660100	0.698000	7.907900
H	0.940900	-0.121900	7.939200
H	1.312500	1.488600	8.574900
H	2.607200	0.322300	8.305800
C	7.641700	2.282500	-0.305400
C	3.008300	4.161600	-1.580800
H	2.203400	4.691700	-2.071800
C	5.334300	3.444400	-1.644500
C	4.250900	4.072800	-2.229000
H	4.357300	4.493800	-3.224700
C	7.795000	2.510900	-1.644000
C	6.872200	3.757700	-3.872900
C	5.184600	2.931800	-0.324300
B	6.663200	3.222100	-2.408600
C	6.307800	2.340900	0.344500
C	8.859600	1.991800	0.498500
C	9.937600	1.349900	-0.140400
C	9.796200	1.029600	-1.604000
H	10.772200	0.807500	-2.038800

H	9.159400	0.146500	-1.739100
C	9.029700	2.448500	1.811300
H	8.241700	3.016300	2.290500
C	7.267100	4.701700	-6.502000
C	7.446400	5.542600	-5.405100
H	7.748600	6.573200	-5.570100
C	6.694900	2.900800	-4.971700
C	10.211400	2.209800	2.502200
H	10.323800	2.574600	3.516000
C	6.892600	3.380000	-6.265800
H	6.753900	2.707200	-7.107800
C	11.246800	1.514100	1.887500
H	12.167500	1.317900	2.424100
C	11.109100	1.100300	0.566400
H	11.929400	0.595600	0.067000
C	9.133500	2.224800	-2.286200
H	8.994600	2.039400	-3.354300
H	9.786100	3.105800	-2.206900
C	6.271300	1.469200	-4.743100
H	5.263800	1.425100	-4.317800
H	6.271200	0.899100	-5.672600
H	6.939400	0.967200	-4.035300
C	7.454800	6.024600	-2.931000
H	8.163300	5.604500	-2.210100
H	7.836100	6.992900	-3.257100
H	6.514000	6.190600	-2.397800

C	7.252500	5.090300	-4.100600
C	7.447000	5.214800	-7.908100
H	8.166200	6.034700	-7.939500
H	7.794500	4.424100	-8.575000
H	6.499800	5.590400	-8.306000

10-BMes Energy = -1901.4859972

	**X**	**Y**	**Z**
C	0.667700	11.312300	3.798000
C	3.987700	15.262400	5.086900
C	4.135800	17.671500	4.789600
C	2.303700	12.976200	2.932200
C	2.774300	15.235600	4.379900
C	4.649000	16.473600	5.284300
H	5.584400	16.483600	5.837300
C	2.931700	17.634100	4.088600
H	2.515400	18.558500	3.697600
C	2.448200	11.705300	0.427000
C	-1.006300	10.227900	5.834600
C	4.563600	13.976600	5.630700
H	3.866100	13.498900	6.325700
C	2.248200	16.437200	3.877800
C	0.269100	9.985200	3.776900
H	0.600900	9.331700	2.982400
C	-0.457900	11.534300	5.990000
C	4.878900	18.969200	4.981500
H	4.190700	19.815000	5.021100
C	3.274800	13.361900	1.966400

H	3.929900	14.194700	2.201800
C	2.464500	11.136400	-0.872600
H	3.269400	11.412600	-1.544800
C	1.469300	10.292500	-1.284700
H	1.483300	9.873900	-2.283700
C	1.470700	11.882200	2.684500
C	0.372800	10.511500	0.858600
H	-0.489900	10.320700	1.480700
C	1.429900	11.329000	1.352500
C	-0.592800	9.469000	4.756300
H	-0.966500	8.455200	4.646800
C	0.390900	10.011100	-0.417300
H	-0.438500	9.409600	-0.768800
C	3.399700	12.698300	0.782100
H	4.172700	12.959500	0.067800
C	0.946200	16.424200	3.112500
H	1.006400	15.749700	2.252600
B	2.019100	13.873700	4.159500
C	-0.763900	12.310700	7.160100
C	-4.083900	8.360600	5.871300
C	-4.232000	5.951500	6.168400
C	-2.399900	10.646800	8.025900
C	-2.870500	8.387400	6.578200
C	-4.745200	7.149400	5.673800
H	-5.680600	7.139400	5.120900
C	-3.027800	5.988900	6.869500

H	-2.611500	5.064500	7.260500
C	-2.544400	11.917700	10.531100
C	0.910100	13.395100	5.123500
C	-4.659900	9.646500	5.327400
H	-3.962400	10.124100	4.632400
C	-2.344400	7.185800	7.080300
C	-0.365300	13.637800	7.181200
H	-0.697100	14.291300	7.975700
C	0.361700	12.088700	4.968100
C	-4.975100	4.653800	5.976600
H	-4.286900	3.808000	5.937000
C	-3.371000	10.261100	8.991700
H	-4.026100	9.428300	8.756300
C	-2.560700	12.486600	11.830700
H	-3.365600	12.210400	12.502900
C	-1.565500	13.330500	12.242700
H	-1.579500	13.749100	13.241800
C	-1.566900	11.740800	8.273500
C	-0.469000	13.111500	10.099500
H	0.393700	13.302300	9.477400
C	-1.526100	12.294000	9.605600
C	0.496600	14.154000	6.201800
H	0.870300	15.167800	6.311300
C	-0.487100	13.611900	11.375300
H	0.342300	14.213400	11.726900
C	-3.495900	10.924700	10.176000

H	-4.268900	10.663500	10.890300
C	-1.042300	7.198800	7.845500
H	-1.102500	7.873300	8.705400
B	-2.115300	9.749300	6.798500
H	-5.666900	4.478700	6.805400
H	-5.560000	4.667300	5.055600
H	-4.850100	10.360300	6.135100
H	-5.598500	9.472600	4.800300
H	-0.785300	6.204300	8.211600
H	-0.221100	7.552900	7.215000
H	5.502200	14.150500	6.157800
H	4.753900	13.262700	4.823000
H	5.463800	18.955700	5.902400
H	5.570600	19.144300	4.152700
H	0.125000	16.070100	3.743000
H	0.689100	17.418700	2.746500

11 Energy = -2132.5450624

	**X**	**Y**	**Z**
C	0.584400	11.304700	3.790300
C	2.352800	15.923200	5.436800
C	4.008000	17.519700	4.702400
C	2.190500	12.974800	2.868800
C	2.680300	15.132300	4.316600
C	3.021300	17.136100	5.603300
H	2.791200	17.794400	6.432600
C	4.334300	16.726700	3.607300

H	5.115300	17.064500	2.936500
C	2.337500	11.700700	0.406400
C	-1.038200	10.258100	5.854900
C	1.279500	15.518800	6.463900
C	3.656700	15.527800	3.383300
C	0.116700	10.000400	3.761000
H	0.418900	9.361500	2.944100
C	-0.438600	11.533700	6.020100
C	3.120600	13.448700	1.900700
C	2.380700	11.106200	-0.884300
H	3.188200	11.385900	-1.552000
C	1.406600	10.239400	-1.295900
H	1.443000	9.804800	-2.287500
C	1.371700	11.862700	2.651000
C	0.274300	10.478400	0.825800
H	-0.600700	10.299300	1.434800
C	1.320100	11.307500	1.325900
C	-0.757600	9.481800	4.746000
C	0.314600	9.960700	-0.441900
H	-0.507000	9.351100	-0.798800
C	3.249000	12.734700	0.734400
H	4.001000	12.984000	-0.006600
C	4.008300	14.685300	2.143200
C	1.911600	15.565500	7.874600
H	2.684000	14.799100	7.973300
H	1.159000	15.403200	8.648300

H	2.365900	16.537300	8.067000
C	0.132300	16.553400	6.385100
H	0.507600	17.556800	6.595200
H	-0.648200	16.323600	7.113700
H	-0.312500	16.551300	5.387700
C	5.465000	14.192100	2.298100
H	6.147100	15.035800	2.421400
H	5.773000	13.628500	1.414600
H	5.555500	13.542000	3.171400
C	3.910200	15.601000	0.901800
H	2.886400	15.959200	0.773400
H	4.204400	15.069000	-0.003200
H	4.567400	16.465100	0.999700
B	1.960200	13.811300	4.120700
C	-0.676700	12.306800	7.213200
C	-3.748900	8.083600	7.620300
C	-4.100400	6.091900	6.301100
C	-2.282700	10.636600	8.134900
C	-2.772600	8.479200	6.687000
C	-4.426600	6.884800	7.396300
H	-5.207400	6.547000	8.067300
C	-3.113900	6.475500	5.400100
H	-2.883900	5.817300	4.570700
C	-2.429400	11.910600	10.597300
C	0.945900	13.353400	5.148600
C	-4.100200	8.926000	8.860700

C	-2.445200	7.688400	5.566600
C	-0.209100	13.611100	7.242500
H	-0.511400	14.250000	8.059400
C	0.346300	12.077800	4.983400
C	-3.212600	10.162600	9.103100
C	-2.472600	12.505200	11.888000
H	-3.280000	12.225400	12.555800
C	-1.498500	13.372100	12.299500
H	-1.534900	13.806700	13.291100
C	-1.463900	11.748700	8.352600
C	-0.366300	13.133100	10.177700
H	0.508600	13.312300	9.568600
C	-1.412100	12.304000	9.677700
C	0.665200	14.129800	6.257500
C	-0.406700	13.650800	11.445400
H	0.414900	14.260500	11.802200
C	-3.340900	10.876600	10.269400
H	-4.092800	10.627200	11.010500
C	-1.372100	8.092900	4.539500
C	-4.001400	8.010100	10.101900
H	-2.977400	7.652200	10.229900
H	-4.295500	8.541800	11.007100
H	-4.658300	7.145700	10.004000
C	-5.557000	9.418900	8.706500
H	-6.239100	8.575100	8.583400
H	-5.864700	9.982300	9.590200

H	-5.648000	10.069100	7.833300
C	-0.225100	7.058000	4.617900
H	-0.600500	6.054800	4.407500
H	0.555600	7.288000	3.889400
H	0.219700	7.059700	5.615400
C	-2.004400	8.046400	3.128800
H	-2.776600	8.813100	3.030400
H	-1.251800	8.208800	2.355100
H	-2.458900	7.074800	2.936400
B	-2.052500	9.800200	6.882900
H	4.531000	18.457100	4.854600
H	-4.623600	5.154500	6.148900

References

S1) Walkinshaw, A. J.; Xu, W.; Suero, M. G.; Gaunt, M. J. *J. Am. Chem. Soc*. **2013**, 135, 12532.

S2) J. J. Murphy, D. Bastida, S. Paria, M. Fagnoni & P. Melchiorre, *Nature*, **2016**, 532, 218–222.

S3) Z. Zhou, A. Wakamiya, T. Kushida, and S. Yamaguchi, J. Am. Chem. Soc. 2012, 134, 4529−4532.

S4) K. Schickedanz, J. Radtke, M. Bolte, H.-W. Lerner and M. Wagner, *J. Am. Chem. Soc.*, **2017**, *139*, 2842–2851.

S5) Gaussian 09, Revision C1, Frisch, M. J.; Trucks, G. W.; Schlegel, H. B.; Scuseria, G. E.; Robb, M. A.; Cheeseman, J. R.; Scalmani, G.; Barone, V.; Mennucci, B.; Petersson, G. A.; Nakatsuji, H.; Caricato, M.; Li, X.; Hratchian, H. P.; Izmaylov, A. F.; Bloino, J.; Zheng, G.; Sonnenberg, J. L.; Hada, M.; Ehara, M.; Toyota, K.; Fukuda, R.; Hasegawa, J.; Ishida, M.; Nakajima, T.; Honda, Y.; Kitao, O.; Nakai, H.; Vreven, T.; Montgomery, Jr., J. A.; Peralta, J. E.; Ogliaro, F.; Bearpark, M.; Heyd, J. J.; Brothers, E.; Kudin, K. N.; Staroverov, V. N.; Kobayashi, R.; Normand, J.; Raghavachari, K.; Rendell, A.; Burant, J. C.; Iyengar, S. S.; Tomasi, J.; Cossi, M.; Rega, N.; Millam, J. M.; Klene, M.; Knox, J. E.; Cross, J. B.; Bakken, V.; Adamo, C.; Jaramillo, J.; Gomperts, R.; Stratmann, R. E.; Yazyev, O.; Austin, A. J.; Cammi, R.; Pomelli, C.; Ochterski, J. W.; Martin, R. L.; Morokuma, K.; Zakrzewski, V. G.; Voth, G. A.; Salvador, P.; Dannenberg, J. J.; Dapprich, S.; Daniels, A. D.; Farkas, Ö.; Foresman, J. B.; Ortiz, J. V.; Cioslowski, J.; Fox, D. J. Gaussian, Inc., Wallingford CT, 2009.

S6) Zhao Y.; Truhlar D. G., Theor. Chem. Acc., **2008**, 120, 215.

S7) Mennucci B.; Cancès E.; Tomasi J., J. Phys. Chem. B, **1997**, 101, 1050.

S8) Nakin Surapanich, Patcharin Chaisuwan, Chutima Kuhakarn, Vichai Reutrakul Synlett 2016; 27(19): 2689-2694.

S9) A. J. Warner, J. R. Lawson, V. Fasano, M. J. Ingleson, *Angew. Chem. Int. Ed.*, 2015, **54**, 11245.

S10) Nowell H, Barnett SA, Christensen KE, Teat SJ, Allan DR. J Synchrotron Radiat., **2012,** *19*, 435-441.

S11) (a) Sheldrick. G. M., *Acta Crystallogr.*, **2015**, *A71*, 3-8. (b) Sheldrick. G. M., *Acta Crystallogr.*, **2015**, *C71*, 3-8. (c) O. V. Dolomanov, L. J. Bourhis, R. J. Gildea, J. A. K. Howard, H. Puschmann,. *J. Appl. Cryst.*, **2009,** *42*, 339–341.
